# Single-gate, multipartite entanglement on a room-temperature quantum register

**DOI:** 10.1038/s41565-026-02254-6

**Published:** 2026-09-14

**Authors:** Joseph D. Minnella, Mathieu Ouellet, Amelia R. Klein, Lee C. Bassett

**Affiliations:** 1 Quantum Engineering Laboratory, Department of Electrical and Systems Engineering, University of Pennsylvania, Philadelphia, PA USA; 2 Department of Physics and Astronomy, University of Pennsylvania, Philadelphia, PA USA; 3 Present Address: School of Applied and Engineering Physics, Cornell University, Ithaca, NY USA

**Keywords:** Quantum information, Electrical and electronic engineering, Semiconductors

## Abstract

Multipartite entanglement is an essential aspect of quantum systems, needed to execute quantum algorithms, implement error correction and achieve quantum-enhanced sensing. In solid-state quantum registers such as nitrogen-vacancy centres in diamond, entangled states are typically created using sequential, pairwise gates between the central electron and individual nuclear qubits. This sequential approach is slow and suffers from crosstalk errors. Here we demonstrate a parallelized multi-qubit entangling gate to generate a four-qubit Greenberger–Horne–Zeilinger state using a room-temperature nitrogen-vacancy centre in only 14.8 μs—ten times faster than that using sequences of two-qubit gates and close to the fundamental limit set by the hyperfine coupling frequencies. Parallel three-qubit gates are also realized with all-nuclear-qubit subsets. The entangled states are verified by measuring multiple quantum coherences. The four-qubit parallel gate has a fidelity of 0.92(4), whereas the sequential four-qubit gate fidelity is only 0.69(3). The approach is generalizable to other solid-state platforms, and it lays the foundation for scalable generation and control of entanglement in practical devices.

## Abstract (web summary)

This work demonstrates a parallelized four-qubit entangling gate on a room-temperature spin register, and it operates ten times faster than sequences of two-qubit gates with reduced errors.

## Main

Quantum registers in solid-state materials are the basis for quantum networking^1–7^, quantum information processing^8–19^ and quantum sensing^20–27^. The nitrogen-vacancy (NV) centre in a diamond is the most well-established solid-state quantum register, and it is especially notable for featuring room-temperature spin coherence. The central electron spin, initialized and measured optically, serves as an interface for detecting and controlling surrounding spins, including ^13^C nuclei^28–30^. Owing to their small gyromagnetic ratios, nuclear spins interact weakly with their environment and have long coherence times, making them attractive memory qubits. Generating high-fidelity entanglement between the electron and multiple nuclei is essential for error correction^11,29^ and enhanced sensing beyond the standard quantum limit^25–27^.

To extend the electron-spin coherence time to be comparable with that of nuclear qubits, it must be decoupled from its environment; this is achieved using dynamical decoupling (DD) control sequences. Crucially, DD sequences can be designed to initialize, control and read-out individual nuclear qubits, all while decoupling the electron from other noise sources^8,29^. At cryogenic temperatures, where coherent optical transitions facilitate high-fidelity read-out and spin lifetimes extend beyond 1 min (refs. ^10,31^), DD sequences have facilitated the realization of quantum networking nodes^1–4^ and preliminary fault tolerance using nuclear quantum registers^11^. At room temperature, where read-out is less efficient^32^ and coherence times are shorter, DD extends coherence times and enables entanglement-assisted enhanced sensing protocols suitable for practical use cases^27,33^. The performance of such networking, computing and sensing schemes remains limited by the speed and fidelity of generating multipartite entangled states.

Using DD sequences to control multipartite entanglement presents two critical challenges. First, each DD sequence is calibrated to generate bipartite entanglement with a single nucleus, thereby requiring long, sequential gates to entangle multiple nuclear qubits. Second, due to the always-on spin–spin interactions within the quantum register, executing sequences of nuclear gates leads to unwanted rotations of all other qubits (that is, crosstalk). Parallelized entangling gates address both these issues. Recent approaches in trapped-ion and neutral-atom platforms include specialized multi-qubit gates^34–36^ and the simultaneous implementation of multiple two-qubit gates^37–39^. For solid-state registers operating in the strong-coupling regime (that is, with hyperfine coupling frequencies larger than the inhomogeneous linewidth of electron spin), multi-qubit entangling gates can be achieved using frequency-selective conditional electron pulses^16–19^. However, strongly coupled registers are rare in nature; therefore, they either need to be engineered^17–19^ or identified through screening^16^. Their scalability is limited by the need for strong, widely spaced couplings for individual addressability. Weakly coupled registers typically offer larger register sizes that enable more robust error correction and networking capabilities^4,10,11^, but the sequential application of DD sequences leads to impractical gate times and errors for multi-qubit control.

**Fig. 1:**
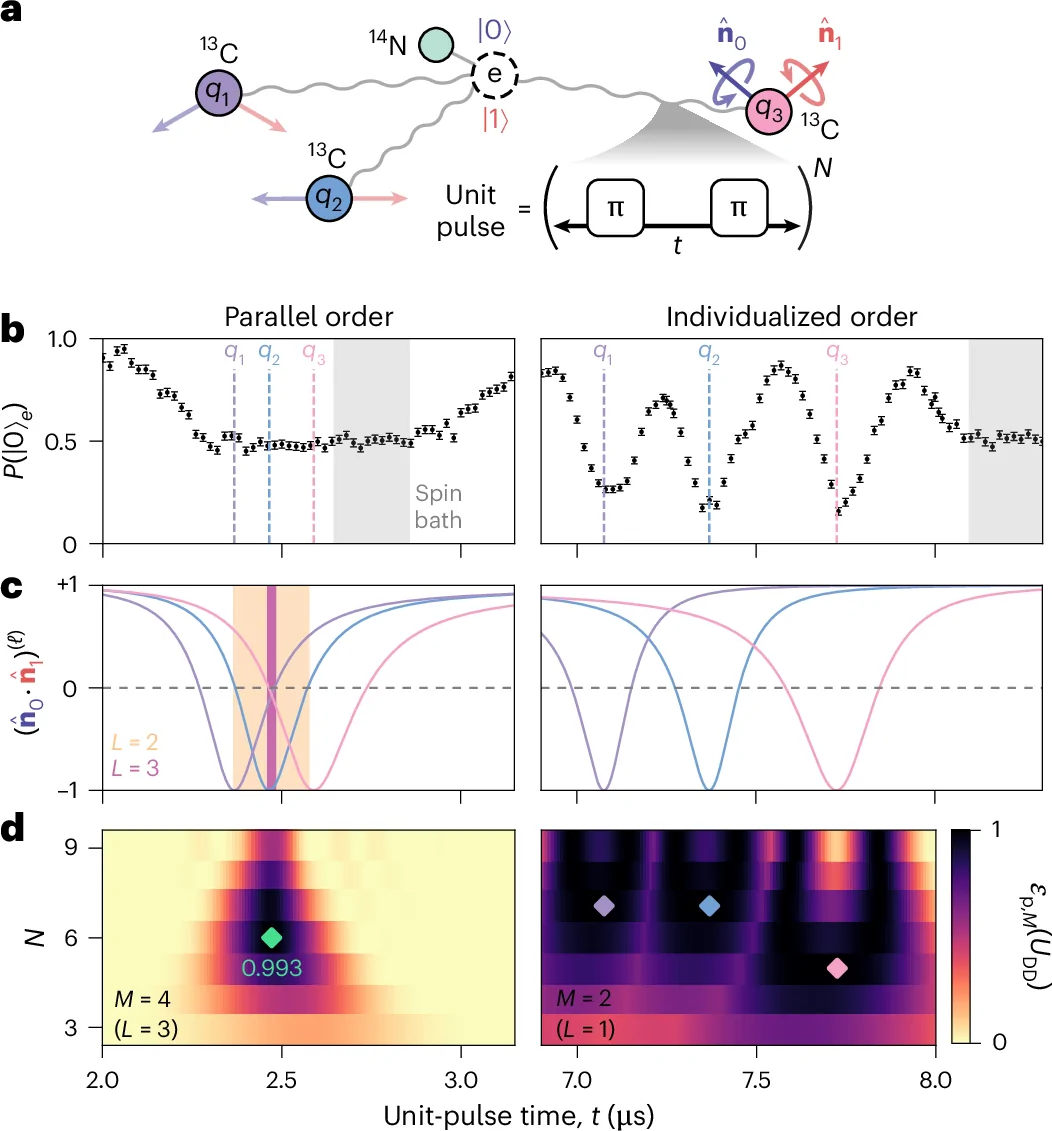
Entanglement through DD in NV quantum registers. **a**, Schematic of the NV centre including nearby ^13^C nuclear qubits *q*_*ℓ*_. Electron–nuclear entangling gates were implemented with XY8 DD, symbolically shown in the inset. Entangling gates rotate nuclear qubits about distinct axes ${\hat{\mathbf{n}}}_{0}$ (dark blue) and ${\hat{\mathbf{n}}}_{1}$ (red), conditioned on the two states of the electron. **b**, DD spectroscopy measurements at *k* = 1, *N* = 6 and *k* = 2, *N* = 12; resonances associated with nuclear qubits *q*_1_, *q*_2_ and *q*_3_ are marked with dashed lines. Data points denote mean values over 10^6^ shots and error bars denote the photon shot noise. Additional spectroscopy data are provided in Supplementary Section IV. **c**, Alignment of the electron-spin-dependent nuclear rotation axes, where −1 (+1) indicates perfectly (un)conditional rotations. The orange- and purple-shaded regions indicate the intersection of unit-pulse times, where ${({\hat{\mathbf{n}}}_{0}\cdot {\hat{\mathbf{n}}}_{1})}^{(\ell )} < 0$ for two and three nuclear qubits, respectively. **d**, *M*-qubit entangling power metric *ε*_p,*M*_ as a function of DD unit-pulse parameters. At the first order, the maximum in *ε*_p,4_ (green diamond) indicates an optimal DD sequence to create maximal four-way entanglement. At the second order, the diamond markers indicate the optimum parameters for two-qubit entangling gates, as the maximum value of each nuclear qubit’s bipartite entangling power, ${\max }_{\ell }({\varepsilon }_{\rm{p},2})$. **b**–**d** share a horizontal axis.

In this work, we design, implement and benchmark a multi-qubit gate using a single DD sequence that facilitates the efficient generation of multipartite entanglement, even at room temperature. We use the entanglement metric framework developed in refs. ^40,41^ to design DD sequences that address multiple weakly coupled ^13^C nuclear qubits surrounding an NV centre (Fig. 1a). Essentially, this approach leverages the crosstalk inherent in DD sequences to simultaneously create conditional rotations of multiple nuclear qubits in which each rotation is locally equivalent to a CNOT gate. We experimentally generate four-qubit GHZ entangled states that include the electron and three weakly coupled ^13^C nuclear qubits, and we verify them by measuring multiple quantum coherences (MQCs). The largest parallel gates are an order of magnitude faster and have substantially higher fidelity than their sequential counterparts.

## Multipartite entanglement with DD

DD control sequences consist of π-pulses applied to the electron, interleaved with precisely timed free evolution periods. Typical DD protocols are constructed using unit sequences in which an even number of π-pulses are spaced by an equal delay, *t*/2, and the unit is repeated *N* times (Fig. 1a). Due to the weak hyperfine interactions, individual nuclear qubits can be coherently controlled through the calibration of *t* and *N* (refs. ^8,29^). Conceptually, *t* sets the period of electron-state flips; when chosen to be in resonance with a target nuclear qubit, the DD sequence creates *X*-axis nuclear rotations. The resonance order *k* corresponds to the number of nuclear precession rotations in each period. As *k* increases, resonances separate due to differences in their hyperfine coupling^28^. Depending on the type of resonance, the nuclear rotations can be conditioned on the electron’s spin state, allowing for two-qubit entangling gates, or they can be unconditional, allowing for local gates in the register.

DD sequences can also be used to sense nearby nuclear qubits^21–23,28^. In DD spectroscopy, the electron is initialized to an equal superposition state, and sequences with varying *t* are applied to identify resonances associated with conditional nuclear rotations. The equation used to determine these resonance times is provided in the ‘Dynamical decoupling’ section. This is typically performed at *k* ≥ 2 so that individual resonances are resolved and can be fit to extract hyperfine couplings (Fig. 1b (right), corresponding to *k* = 2). At the first order (Fig. 1b (left), corresponding to *k* = 1), the nuclear qubits’ resonances generally overlap with each other and with the spin bath. For these reasons, first-order resonances are usually overlooked, yet this region is ideal for realizing parallelized entangling gates.

The nature of DD spectroscopy resonances, and correspondingly the associated DD entangling gates, is captured by the relative orientation of the electron-state-dependent nuclear rotation axes, $${\hat{\mathbf{n}}}_{{m}_{s}}^{(\ell )}$$. Traditionally, entangling gates are achieved by tuning *t* to a *k*th-order resonance at which the axes for the *ℓ*th nuclear qubit are antiparallel, or $${({\hat{\mathbf{n}}}_{0}\cdot {\hat{\mathbf{n}}}_{1})}^{(\ell )}=-1$$ (Fig. 1c). With *N* chosen to set the rotation angle to π/2, the two-qubit entangling gate takes the form $${C}_{e}{X}_{\ell }(\pm \pi /2)=$$$$|0\rangle {\langle 0|}_{e}\otimes {X}_{\ell }(\pi /2)+$$$$|1\rangle {\langle 1|}_{e}\otimes {X}_{\ell }(-\pi /2)$$, hereafter denoted as *CX*_π__/2_. Similarly, unconditional *X* gates occur when $${({\hat{\mathbf{n}}}_{0}\cdot {\hat{\mathbf{n}}}_{1})}^{(\ell )}=+1$$. Other nuclear qubits that are not in resonance for a given *t* experience unconditional rotations about *Z*. This crosstalk is an inherent characteristic of quantum register control using DD sequences and must be considered in quantum circuit design. Additional details regarding DD are provided in the ‘Dynamical decoupling’ section.

Generalizing the traditional approach, refs. ^40,41^ showed that certain near-resonance DD sequences can still act as maximal entangling gates, as long as ${({\hat{\mathbf{n}}}_{0}\cdot {\hat{\mathbf{n}}}_{1})}^{(\ell )} < 0$. Crucially, if this condition is satisfied for multiple nuclear qubits for the same *t*, then a single DD sequence can act as a maximal, multipartite entangler. The multipartite entangling ability of a DD sequence is quantified by the *M*-qubit entangling power *ε*_p,*M*_. Here *M* includes the electron qubit together with *L* nuclear qubits; therefore, *M* = *L* + 1. As shown in Fig. 1c, at parallel order, the entangling condition is simultaneously satisfied for three ^13^C qubits (*q*_1_, *q*_2_ and *q*_3_) proximal to the NV centre (*e*) in our experiment, and the 4-qubit entangling power reaches a maximum at *ε*_p,4_ = 0.993 (Fig. 1d). Additional details regarding the entanglement metrics are provided in the ‘Entanglement metrics’ section.

## Verifying entanglement with MQCs

In general, the characterization of arbitrary multi-qubit states requires complete state tomography, with measurement requirements that scale exponentially with the number of qubits. Moreover, full tomography involves the measurements of correlated observables, which demands the high-fidelity read-out of all qubits. In the room-temperature NV-quantum-register system, where limited-fidelity read-out is only available for the electron, tomography quickly becomes impractical.

**Fig. 2:**
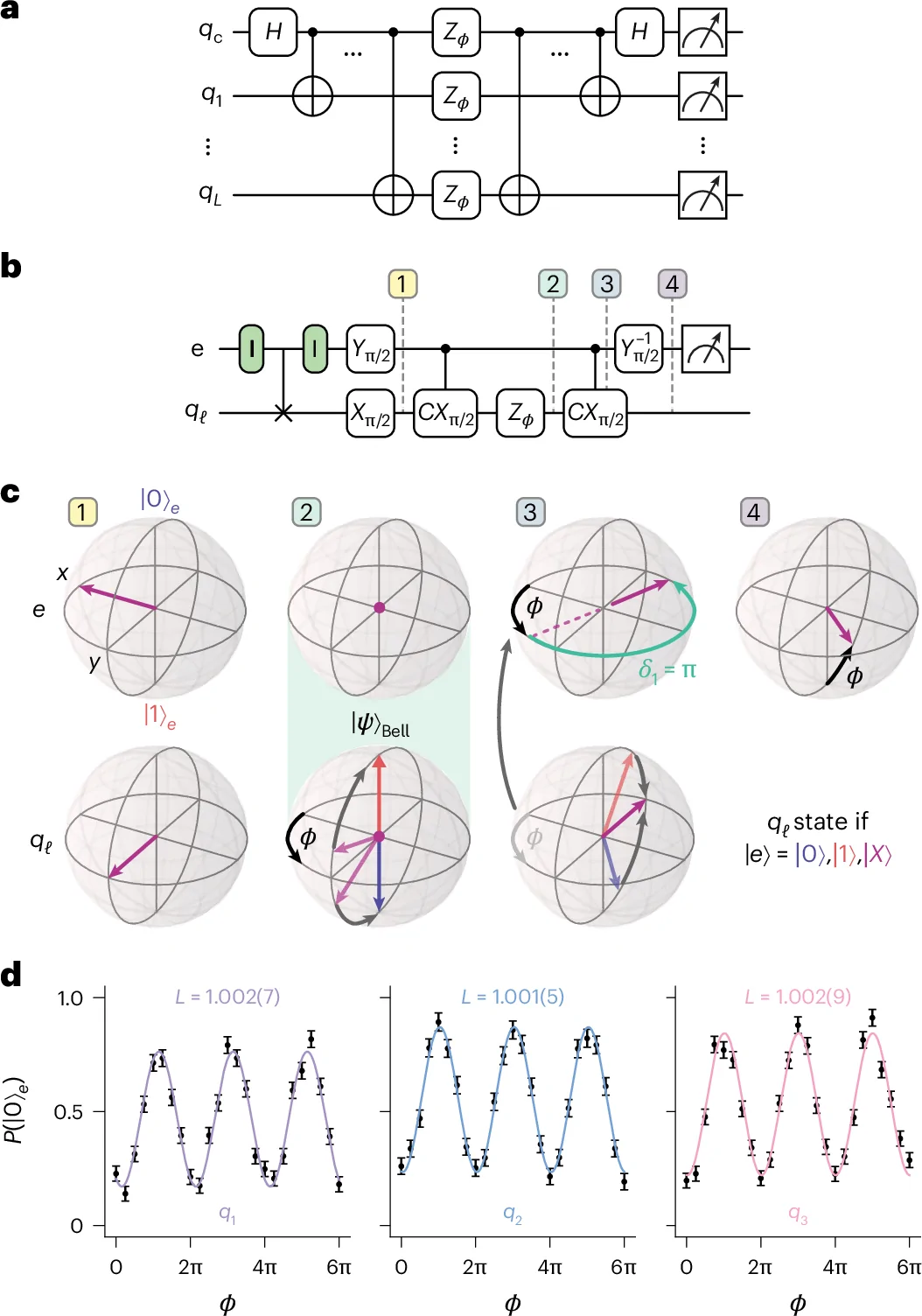
Verifying entangled states using MQCs. **a**, Quantum circuit to efficiently characterize entangled states using the MQC method^43^. **b**, DD implementation of the MQC method used in this work (shown here for bipartite entanglement), in which only the nuclear qubits are subject to *Z*_*ϕ*_ gates. **c**, Bloch-sphere visualization of the stages labelled in **b**. First, both spins are initialized to $\left\vert 0\right\rangle$ and rotated to equatorial superposition states (step 1). The conditional rotation that follows creates a Bell state, and a variable phase *ϕ* is added to the nuclear qubit (step 2). When the qubits are disentangled, *ϕ* is added to the electron phase (step 3) and the final electron rotation maps *ϕ* to an angle from $\hat{z}$, which is then measured through spin-dependent fluorescence (step 4). **d**, Results of MQC experiments for bipartite entanglement (*M* = 2 total qubits, *L* = 1 nuclear qubit) with each nuclear qubit, *q*_*ℓ*_. Data points denote mean values over 10^6^ shots and error bars denote the photon shot noise. Uncertainties on *L* are 68% confidence intervals from from fits to equation (2) with a variable amplitude, midpoint and phase.

Entanglement verification using MQC offers an alternative approach (Fig. 2a). MQC verification leverages the symmetry of GHZ entangled states to amplify each qubit’s phase accumulation onto a single qubit, which then carries information about the number of qubits in the entangled state^42,43^. When *M* entangled qubits each acquire a phase *ϕ*, the resulting MQC signal is given by

1 $$P\left({\left\vert 0\right\rangle }^{\otimes M}\right)=\frac{1}{2}(1+\cos (M\phi )).$$

Additional details are provided in the ‘Multiple quantum coherences’ section.

### Bipartite entanglement

As an example of this approach, we adapt the MQC circuit to use the DD gates for the creation and verification of two-qubit Bell states (Fig. 2b). In contrast to the original implementation^43^, we measure only the electron qubit; this is sufficient to quantify the size of the entangled state, although not its fidelity. The calibration of all DD sequences, including local and entangling gates as well as nuclear initialization via electron–nuclear swaps^29^, was experimentally verified using single-qubit nuclear state tomography (Supplementary Section V). Figure 2c highlights the key steps of the DD-gate MQC process. Given the form of the *CX*_π/2_ entangling gate, local gates are required to prepare a *Z*-basis Bell state, $\left\vert {{{\varPsi }}}^{+}\right\rangle =\frac{1}{\sqrt{2}}(\left\vert 01\right\rangle +i\left\vert 10\right\rangle )$. The local gates affect the relative phase of the entangled state and shift the phase of the MQC signal in equation (1) without affecting the frequency. Additionally, we only apply the variable phase gate *Z*_*ϕ*_ to the nuclear qubits. As a result, when the electron is entangled with *L* nuclear qubits, we expect to measure a signal of the form

2 $$P\left({\left\vert 0\right\rangle }_{e}\right)=\frac{1}{2}(1+\cos (L\phi -{\delta }_{L})),$$

where *δ*_*L*_ is the phase shift that depends on local gates; for the bipartite case, *δ*_1_ = π. As shown in Fig. 2d, the results of this experiment are consistent with the generation of bipartite entanglement between qubit *e* and each of *q*_1_, *q*_2_ and *q*_3_. In particular, the single-frequency MQC signal verifies the existence of bipartite (*L* = 1) entanglement and precludes the unintentional presence of additional entangled qubits. This is expected from resolved DD resonances at the individualized order (Fig. 1).

### Multipartite entanglement

**Fig. 3:**
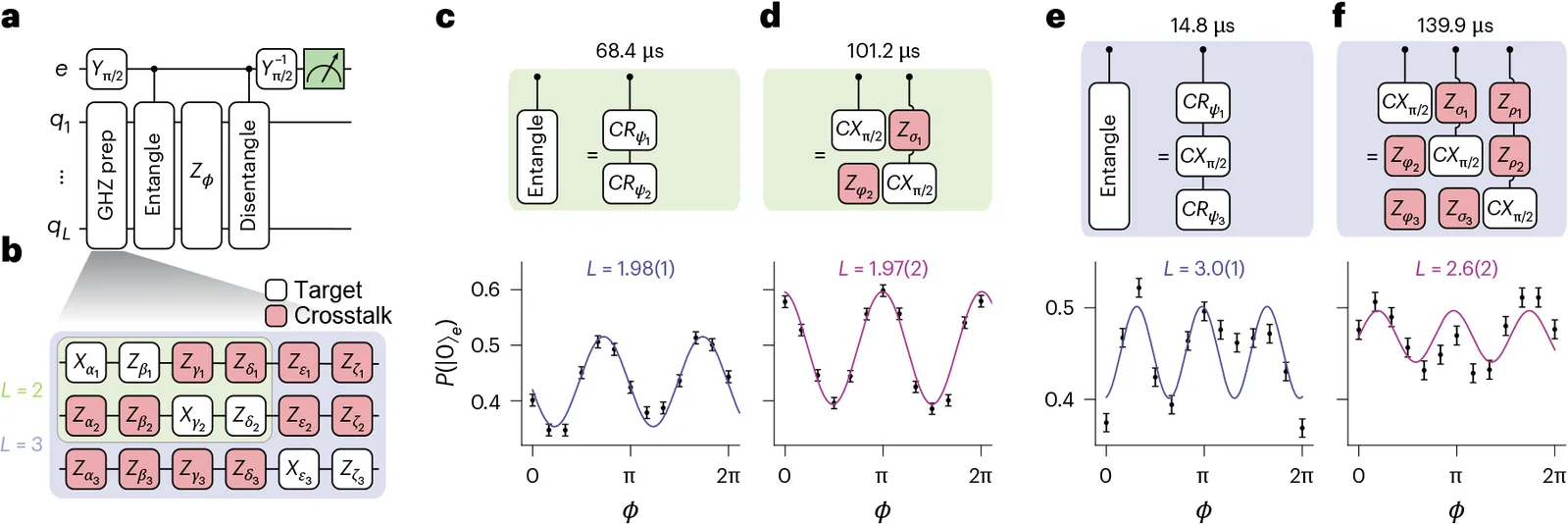
Generation and verification of multipartite GHZ states. **a**, Block-diagram quantum circuit for entanglement verification using multiple nuclear qubits. Qubits are initialized sequentially, as shown in Fig. 2b. **b**, Local gates used to prepare for the GHZ state generation for *L* = 2 or 3 nuclear qubits (green- and blue-shaded regions, respectively). The red-shaded gates represent unwanted crosstalk effects, which manifest as *Z*-axis rotations on each nuclear qubit in the register that is not targeted with a white gate. The angles of the local gates (associated with the unit-pulse repeats) were determined using numerical optimization, with the gate axes (associated with unit-pulse times) remaining fixed, as shown. **c**,**d**, Results of three-qubit (*L* = 2) MQC verification experiments using nuclear qubits *q*_1_ and *q*_2_ (data points are shown in the bottom panels) corresponding to the parallel (**c**) and sequential (**d**) entangling gates shown in the top panel. The corresponding disentangling gates in **a** are identical. The red (parallel) and blue (sequential) curves are sinusoidal fits to measure the number of entangled nuclear qubits *L*. Supplementary Section IX shows the results corresponding to all other combinations of qubits. **e**,**f**, Results of four-qubit (*L* = 3) MQC verification experiments for parallel (**e**) and sequential (**f**) entangling gates. The parallel-gate unit-pulse time is effectively on resonance for *q*_2_, creating a *CX*_π/2_ rotation. The data points in **c**–**f** denote mean values over 10^6^ shots and the error bars denote the photon shot noise. Uncertainties on *L* are 68% confidence intervals from from fits to equation (2).

We consider and compare two methods to generate multipartite (*L* ≥ 2) entangled states: through sequential one- and two-qubit gates at individualized order; and through a single, parallel gate at first order. Although the approach to verifying multipartite entanglement is similar to the bipartite setting, quantum circuits become more complicated (Fig. 3a). Neither gate takes the form $C{X}_{\uppi /2}^{\otimes L}$ on the register. In the parallel case, this is because the rotation geometry is unique for each qubit, with some nuclear qubits conditionally rotated off-resonance according to $C{R}_{\theta }=\left\vert 0\right\rangle {\left\langle 0\right\vert }_{e}\otimes {R}_{{\hat{\mathbf{n}}}_{0}}(\theta )+\left\vert 1\right\rangle {\left\langle 1\right\vert }_{e}\otimes {R}_{{\hat{\mathbf{n}}}_{1}}(\theta )$. In the sequential case, this is because each individualized DD sequence creates crosstalk errors on the other qubits.

In either case, it is necessary to apply local gates before entanglement to prepare the desired GHZ state (Fig. 3b). These gates set the basis of each qubit, compensating for the crosstalk effects of the sequential protocol or matching the parallel entangling gate’s geometry. Although the necessary local gates can, in principle, be predicted based on analytical theory, it is more effective, in practice, to optimize them numerically, especially since the local gates themselves suffer from crosstalk. Supplementary Section IX shows the optimization details and the ‘Experimental MQC considerations’ section provides additional experimental considerations.

Starting with *L* = 2 (two nuclear qubits), we can design a parallel three-qubit entangling gate with any subset of (*q*_1_, *q*_2_, *q*_3_). Figure 3c shows the example for *e*, *q*_1_ and *q*_2_. Since any residual entanglement with the spectator nuclear qubit will lower the entangling-gate fidelity, the *L* = 2 gate parameters must be selected such that crosstalk on the spectator qubit is unconditional (Supplementary Section V). The MQC method is also sensitive to such effects; residual entanglement manifests as multi-frequency beating in the signal (Supplementary Section IX provides theoretical analysis and example data). The single-frequency oscillation observed in Fig. 3c, with *L* = 2, confirms that the gate creates a tripartite entangled state, as desired. The sequential approach (with optimized crosstalk corrections) also generates a tripartite state with the expected MQC signal (Fig. 3d); however, the sequential gate duration is almost 50% longer than the parallel gate.

Figure 3e,f shows the results of MQC verification experiments for 4-qubit GHZ states (*L* = 3). The advantages of parallel gates increase with the system size; the parallel *L* = 3 gate is almost ten times faster than the corresponding sequential gate. Moreover, we observe the expected MQC frequency at *L* = 3 from the parallel gate, whereas the sequential approach results in a shifted frequency of *L* = 2.6(2). This discrepancy reflects the lower fidelity of the sequential gate (shown in the next section); essentially, the electron accumulates unwanted phase contributions from crosstalk as each nuclear qubit is sequentially entangled and disentangled in the MQC protocol.

## Entangling-gate fidelities

**Fig. 4:**
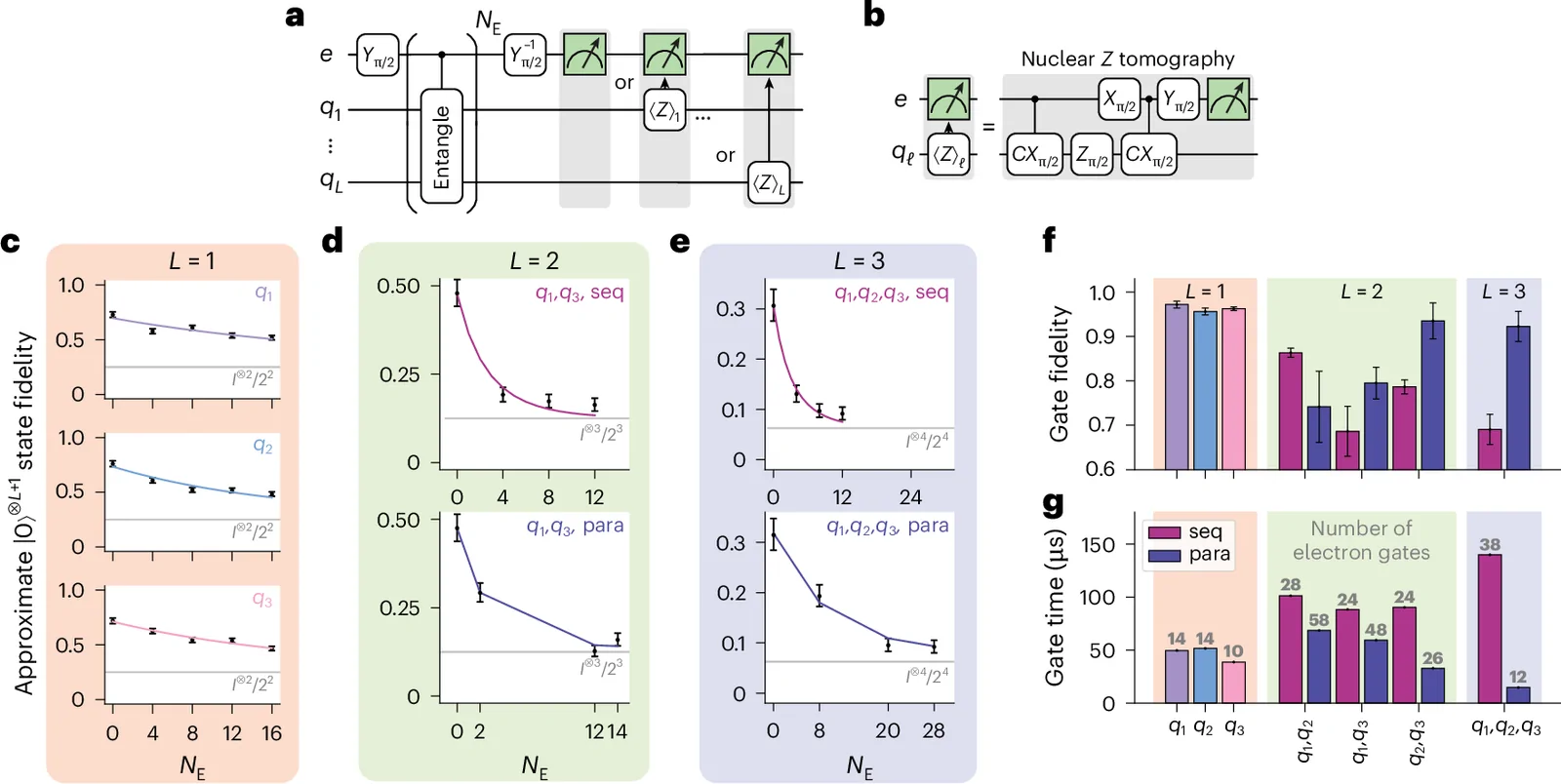
Entangling-gate fidelities. **a**, Quantum circuit to efficiently measure the entangling-gate fidelity. After repeating the entangling gate *N*_E_ times, the *Z* projection of either the electron or an individual nuclear qubit is measured, and the combined results are used to approximate the ${\left\vert 0\right\rangle }^{\otimes M}$ state fidelity. **b**, Sub-circuit to measure the nuclear-qubit *Z* projections. **c**, Experimental results (data points) for bipartite entangling gates associated with each nuclear qubit. The solid curves are fits using a noisy quantum channel model to extract the entangling-gate fidelity. **d**,**e**, Experimental results (data points) for three-way and four-way entanglement generated either sequentially (seq; top) or using a parallel (para) gate (bottom). Repeating the parallel gate does not generally return exactly ${\left\vert 0\right\rangle }^{\otimes M}$ due to its more complicated geometry; values of *N*_E_ were chosen to maximize the overlap with ${\left\vert 0\right\rangle }^{\otimes M}$. Fits to the data (solid curves) account for this effect along with gate errors and are used to determine the fidelity of each gate. Data points in **c**–**e** denote the mean values over 10^6^ shots and the error bars are propagated from each measurement’s photon shot noise. **f**, Entangling-gate fidelities determined from fits to the data in **c**–**e**. The error bars denote 68% confidence intervals from fits to the noisy quantum channel model. **g**, Entangling-gate durations. The number of electron pulses (equivalent to 2*N*) are printed above each bar.

To quantify the entangling-gate fidelity, it is necessary to distinguish the errors associated with the entangling gates from those due to state preparation and measurement (SPAM). We adapt the method presented in ref. ^36^ for this purpose. The key idea is to repeatedly apply the entangling gate and measure the state fidelity after each iteration. Gate errors accumulate with each repetition and decrease the state fidelity, whereas SPAM errors remain constant. In our case, repeating the entangling gate by certain multiples (*N*_E_) ideally results in a disentangled state of the form ${\left\vert 0\right\rangle }^{\otimes M}$, the fidelity of which can be quantified using only a small number of measurements (Fig. 4a). If one further makes the approximation that each final experimental state is separable, for example, $\langle {Z}_{e}\otimes {Z}_{{q}_{\ell }}\rangle \approx \langle {Z}_{e}\rangle \langle {Z}_{{q}_{\ell }}\rangle$ in the bipartite case, then the state fidelity can be approximated from independent measurements of each qubit’s *Z* projection. The electron is measured directly, and the nuclear qubits are measured using *Z*-axis state tomography^29^ (Fig. 4b).

In the bipartite case, applying the *CX*_π/2_ gate in multiples of four returns the system to its initial state of $\left\vert 00\right\rangle$, up to gate errors. Figure 4c shows the results of this experiment using each of *q*_1_, *q*_2_ and *q*_3_, where the state fidelity steadily decays towards the classically mixed asymptote. The multipartite sequential entangling gates have the analogous property of returning to the ideal state ${\left\vert 0\right\rangle }^{\otimes M}$ when *N*_E_ is a multiple of four (Fig. 4d,e, top). In the parallel approach, the gates’ complicated geometry makes the analysis more difficult. We simulate the application of repeated parallel gates to identify which *N*_E_ values most closely approximate ${\left\vert 0\right\rangle }^{\otimes M}$ (Supplementary Section X). The simulated density matrices for each *N*_E_ were then used as starting points in our fitting model, as described below.

The state-fidelity data shown in Fig. 4c–e were fit using a quantum depolarizing channel model to calculate the constant SPAM error, *ε*_SPAM_, and entangling-gate error, *ε*_gate_. Supplementary Section X provides details and additional *L* = 2 datasets. The entangling-gate fidelity, ${G}_{M}={(1-{\varepsilon }_{{\rm{gate}}})}^{M}$, is shown in Fig. 4f. We observe an average (standard deviation) bipartite gate fidelity of ${G}_{2}^{{\rm{avg}}}=0.96(1)$. The three-qubit gate fidelities vary substantially: the parallel-gate fidelities are anti-correlated with how many electron operations are needed (Fig. 4g), whereas the sequential gates are further limited by their intrinsic crosstalk. Nonetheless, the average fidelity for the parallel gates, ${G}_{3}^{{\rm{para}}}=0.83(9)$, exceeds that of the sequential approach, ${G}_{3}^{\,\mathrm{seq}\,}=0.78(8)$, and the parallel gates are almost two times faster. For *M* = 4, the advantages of the parallel approach become dramatically apparent. The four-qubit parallel gate is the fastest gate overall, having a similar number of pulses as the two-qubit gates but a shorter *t*; this contributes to its high fidelity of ${G}_{4}^{{\rm{para}}}=0.92(4)$, despite the large number of qubits involved. The sequential gate, on the other hand, exhibits a low fidelity of ${G}_{4}^{\,\mathrm{seq}\,}=0.69(3)$, due to the combined impact of crosstalk, its longer duration and a large number of electron pulses.

## Generality and extensions

**Fig. 5:**
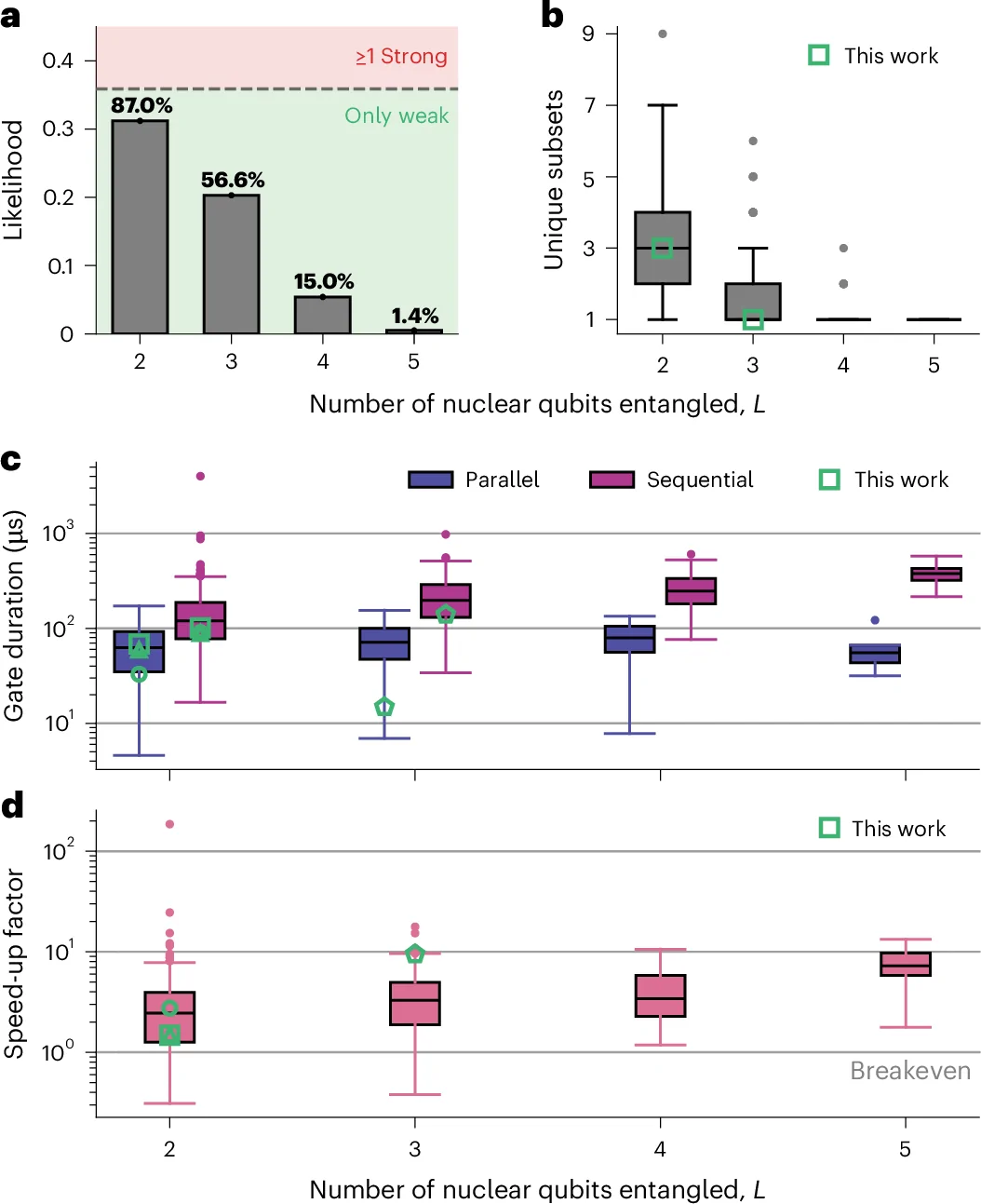
Generality of parallel entanglement in NV-nuclear registers. **a**, Likelihood of a register supporting parallel entanglement. In this analysis, we exclude registers that contain at least one strongly coupled nuclear qubit (red region, corresponding to 64% of configurations). The percentages listed above each bar correspond to the likelihood of weakly coupled registers (green region) supporting parallel entanglement with at least one set of *L* nuclear qubits. On average, the weakly coupled registers contain 5.7 (2.5) individually addressable nuclear qubits in total (Supplementary Section VI). **b**, Number of unique subsets of *L* nuclear qubits in registers hosting parallel entanglement. The middle black line represents the median, the box spans the interquartile range (IQR; 25th–75th percentile), the whiskers extend to 1.5× IQR and points indicate outliers. **c**, Parallel and *k* = 2 sequential entangling-gate durations. **d**, Parallel entanglement speed-up factor.

To contextualize these results, we simulated 500 random, weakly coupled NV-nuclear-qubit registers to evaluate their capacity for parallel entanglement. We excluded configurations with one or more strongly coupled qubits, although such mixed systems could potentially be addressed, too. For each remaining configuration, we determined which combinations of nuclear qubits could be entangled in parallel, and we calculated the corresponding gate parameters for both parallel and sequential operations. The results, summarized in Fig. 5, illustrate that *L* = 2 and *L* = 3 parallel entanglement gates are available in the majority of configurations (Fig. 5a). In some cases, entangled states of four or even five nuclear qubits can be created. Furthermore, the registers generally support the parallel entanglement of multiple unique qubit combinations (Fig. 5b). The parallel-gate duration remains approximately constant as *L* increases, whereas the sequential gates steadily increase (Fig. 5c). Thus, the speed-up of the parallel approach becomes more pronounced with increasing *L* (Fig. 5d). Further simulation details are provided in the ‘Generality and extensions’ section, with additional simulations provided in Supplementary Section VI.

The experimental results reported here fall within the simulated distributions. In future work, registers that enable *L* = 4 or *L* = 5 parallel entangling gates can be reasonably identified. The *L* = 5 case is of particular importance for fault-tolerant operation^11^; Supplementary Section XI provides further discussion and simulations of quantum error correction.

## Conclusions

In demonstrating a method for generating large entangled states quickly, efficiently and with high fidelity, we have addressed a principal challenge of quantum control in central spin systems like diamond NV centres. The always-on nature of qubit-coupling interactions in such systems leads to unavoidable crosstalk, and operations composed only of pairwise gates become impractically long. Rather, our approach directly harnesses the star topology of the central spin system to achieve parallelized entangling gates that are an order of magnitude faster than their sequential counterparts, and more immune to crosstalk. In particular, and in contrast to approaches developed for strongly coupled registers^16–19^, the parallel-gate speed is not limited by the need to resolve differences in parallel hyperfine couplings, *A*_∥_. In fact, the four-qubit gate duration of 15 μs is near the fundamental interaction limit given by the inverse of the perpendicular hyperfine couplings (*A*_⊥_ ≈ 60 kHz for all three qubits).

The parallel entanglement framework and experimental methods presented here are readily applicable to other defects in diamond^3,5,7,14,15,44^ and other host materials like silicon carbide^12,13^ or silicon^17–19^. The density of nuclear spins can also be tuned using isotopic engineering^45^ to optimize the distribution of similarly coupled nuclear qubits to be entangled in parallel.

With a fidelity of 0.92(4), the four-qubit parallel entangling gate is comparable with the state of the art in highly engineered quantum computing systems such as trapped ions^35^ and neutral atoms^36^. The entangled-state fidelities are primarily limited by SPAM errors associated with the NV centre’s room-temperature properties. These can be solved by working at cryogenic temperatures^46^. Alternatively, the room-temperature SPAM errors can be reduced using repetitive read-out at high magnetic fields^47^, spin-to-charge conversion^48^ or dynamical nuclear polarization methods^49^.

These results directly impact entanglement-enhanced quantum sensing protocols, which generally rely on the generation of high-fidelity GHZ states to reach the Heisenberg sensing limit^26,33^. The potential of generating such states at room temperature will facilitate their use in practical applications such as nano-nuclear magnetic resonance and scanning magnetometry. Additionally, the dramatic speed-up from paralleled entangling gates opens the door to new quantum error correcting possibilities^11,40^, to the point that fault tolerance could be achieved at room temperature. In the low-temperature regime in which entanglement can be distributed between quantum registers using photons^2^ and preliminary fault tolerance has been demonstrated^11^, replacing sequences of two-qubit gates with parallelized multi-qubit gates will increase the circuit efficiency and enable the implementation of larger quantum codes.

## Methods

### Experimental system

The sample used in this work is a type-IIa electronic-grade synthetic diamond (Element Six) with a natural abundance of ^13^C impurities. The NV centre is at the focus of a solid immersion lens encircled by an antenna for microwave (mw) frequency control. All experiments are performed at room temperature in ambient conditions. A permanent magnet was aligned to the NV symmetry axis using pulsed electron-spin resonance experiments and positioned to create a magnetic-field strength of 338 G. The magnetic-field strength was chosen to minimize nuclear-qubit gate durations and angular errors. Further details of the field alignment and simulations to determine the field strength are provided in Supplementary Section VII.

Green (532-nm) laser pulses of 2 μs were used to (re)initialize the electron spin and charge state through optical pumping, and shorter 300-ns pulses were used to measure the spin-state photoluminescence contrast. The synchronization of the optical and mw signals was achieved using two different configurations. The first used two arbitrary waveform generators, one (Tektronix AWG520) dedicated to optical control and the other (Tektronix AWG7102), for mw control. The second configuration used a Swabian Instruments PulseStreamer 8/2 for both optical and mw control. Additional details are provided in Supplementary Section I. Electron gate errors were quantified using bootstrap tomography of pulses^50^ (Supplementary Section II).

### DD

The Hamiltonian governing the central spin electron interacting with *L* nuclear qubits is given by

3 $$H={\mathbb{1}}\otimes \frac{{\omega }_{{\rm{Lar}}}}{2}\mathop{\sum }\limits_{\ell =1}^{L}{\sigma }_{z}^{(\ell )}+\frac{{Z}_{e}}{2}\otimes \mathop{\sum }\limits_{\ell =1}^{L}({A}_{| | }^{(\ell )}{\sigma }_{z}^{(\ell )}+{A}_{\perp }^{(\ell )}{\sigma }_{x}^{(\ell )}),$$

where *ω*_Lar_ is the nuclear Larmor frequency; ${Z}_{e}={s}_{0}\left\vert 0\right\rangle \left\langle 0\right\vert +{s}_{1}\left\vert 1\right\rangle \left\langle 1\right\vert$ is the electron-spin operator, where *s*_*j*_ are the two electron-spin projections chosen as the computational basis (*s*_0_ = 0 and *s*_1_ = −1 for this work); and ${A}_{\!\parallel,\!\perp }^{(\ell )}$ are the parallel and perpendicular hyperfine couplings between the electron and the *ℓ*th nuclear qubit. This can be rewritten as^40^

4 $$H=\sum _{j\in \{0,1\}}\left\vert j\right\rangle {\left\langle j\right\vert }_{e}\otimes \mathop{\sum }\limits_{\ell }^{L}{H}_{j}^{(\ell )},$$

where each ${H}_{j}^{(\ell )}$ is given by

5 $${H}_{j}^{(\ell )}=\frac{{\omega }_{L}+{s}_{\!j}{A}_{| | }^{(\ell )}}{2}{\sigma }_{z}^{(\ell )}+\frac{{s}_{\!j}{A}_{\perp }^{(\ell )}}{2}{\sigma }_{x}^{(\ell )}.$$

The notation ${\sigma }_{i}^{(\ell )}$ in equation (5) means the *i*th Pauli matrix on the *ℓ*th component of the *L*-nuclear-qubit Hilbert space and the identity on all other components. This form of the Hamiltonian highlights how the electron-state conditions are different with unique dynamics for each nuclear qubit. This is further made apparent by the free evolution operator *U*_f_(*t*) for the system:

6 $${U}_{f}(t)=\sum _{j\in \{0,1\}}\left\vert j\right\rangle {\left\langle j\right\vert }_{e}\mathop{\bigotimes }\limits_{\ell }^{L}\exp \left(-{\rm{i}}t{H}_{j}^{(\ell )}\right),$$

from which each $\exp (-{\rm{i}}t{H}_{j}^{(\ell )})$ term can be viewed as a rotation operator acting on the *ℓ*th nuclear qubit. Note a subtle shift in notation from equation (5) to (6), where each index *ℓ* no longer implies identity operators on the other qubits, and each two-dimensional ${H}_{j}^{(\ell )}$ can be viewed as acting on a distinct subspace. Additional details and derivations are provided in Supplementary Section III.

The free evolution periods of DD sequences leverage equation (6) to control the rotational effects of each nuclear qubit, as well as extend the electron coherence time. The net unitary operator *U*_DD_ from performing a time-symmetric DD sequence of unit-pulse time *t* and with *N* repeats is given by

7 $${U}_{{\rm{DD}}}=\sum _{j\in \{0,1\}}\left\vert j\right\rangle {\langle j\vert }_{e}\mathop{\bigotimes }\limits_{\ell }^{L}{R}_{{\hat{\mathbf{n}}}_{j}^{(\ell )}(t)}(N{\phi }^{(\ell )}(t)),$$

where *R* is a spin-1/2 rotation operator about the axis ${\hat{\mathbf{n}}}_{j}^{(\ell )}$ and by an angle of *Nϕ*^(*ℓ*)^ for the *ℓ*th nuclear qubit. Supplementary Section V provides details in calculating each rotation operator based on the hyperfine couplings of the register. This formulation highlights the conditional nature of each nuclear qubit’s rotation depending on the electron state ${\left\vert j\right\rangle }_{e}$.

Resonant *X*-axis control of a target nuclear qubit is achieved with the proper choice of unit-pulse time *t*_*m*_ that creates ${({\hat{\mathbf{n}}}_{0}\cdot {\hat{\mathbf{n}}}_{1})}^{(\ell )}({t}_{m})=\pm 1$. Such a choice of *t*_*m*_ occurs periodically and is given by

8 $${t}_{m}^{(\ell )}=\frac{4\uppi m}{{\omega }_{0}^{(\ell )}+{\omega }_{1}^{(\ell )}},$$

for $$m\in {{\mathbb{Z}}}^{+}$$ and $${\omega }_{j}^{(\ell )}=\sqrt{{({s}_{\!j}{A}_{\perp }^{(\ell )})}^{2}+{({\omega }_{L}+{s}_{\!j}{A}_{\parallel }^{(\ell )})}^{2}}$$ (ref. ^40^). For odd *m* = 2*k* + 1, $${({\hat{\mathbf{n}}}_{0}\cdot {\hat{\mathbf{n}}}_{1})}^{(\ell )}=-1$$ and for even *m* = 2*k*, $${({\hat{\mathbf{n}}}_{0}\cdot {\hat{\mathbf{n}}}_{1})}^{(\ell )}=+1$$. Here the integer *k* specifies the DD order, as discussed earlier. When the electron-state-dependent nuclear rotation axes are maximally anti-aligned, or $${({\hat{\mathbf{n}}}_{0}\cdot {\hat{\mathbf{n}}}_{1})}^{(\ell )}=-1$$, the nuclear rotations are maximally dependent on the state of the electron. With *N* set to create the correct rotation angle, the net gate is $${C}_{e}{X}_{\ell }(\pm \pi /2)=$$$$|0\rangle {\langle 0|}_{e}\otimes {X}_{\ell }(\pi /2)+$$$$|1\rangle {\langle 1|}_{e}\otimes {X}_{\ell }(-\pi /2)$$ between the electron and target nuclear qubit *q*_*ℓ*_. For all other spins, the choice of *t* is off-resonance, and the resulting rotation is unconditional and about the *Z* axis. Similarly, when the unit-pulse time is on resonance and $${({\hat{\mathbf{n}}}_{0}\cdot {\hat{\mathbf{n}}}_{1})}^{(\ell )}=+1$$, the resulting *ℓ*th nuclear qubit’s rotation is an unconditional *X*-axis rotation, with all other nuclear rotations being off-resonance and about the *Z* axis.

For example, when attempting to rotate the first nuclear qubit unconditionally about the *X* axis by π/2, the net unitary acting on the register would take the form $U={I}_{e}\otimes {X}_{\uppi /2}\otimes {Z}_{{\theta }_{(2)}}\ldots \otimes {Z}_{{\theta }_{(L)}}$, where each crosstalk rotation angle *θ*_(*ℓ*)_ depends on the choice of *t* and *N* that were used to achieve the desired *X*_π/2_ rotation of *q*_1_ and the specific hyperfine couplings of the *ℓ*th nuclear qubit. The goal of the parallelized entangling gate is to leverage this crosstalk in such a way that each nuclear qubit can be maximally entangled for a single choice of *t* and *N*. Further information on the *t* and *N* parameter choices for each nuclear qubit’s gate, together with their experimental verification, is provided in Supplementary Section VIII.

### Entanglement metrics

To quantify the bipartite entangling ability of a DD sequence with a particular nuclear qubit *q*_*ℓ*_, one can calculate the first Makhlin invariant, which takes the form

9 $${G}_{1}^{(\ell )}={\left({\cos }^{2}\frac{N{\phi }^{(\ell )}}{2}+{({\hat{\mathbf{n}}}_{0}\cdot {\hat{\mathbf{n}}}_{1})}^{(\ell )}{\sin }^{2}\frac{N{\phi }^{(\ell )}}{2}\right)}^{2},$$

for time-symmetric DD sequences such as XY8 (ref. ^40^). This entanglement metric (bounded from 0 to 1) is minimal when bipartite entanglement is maximal. Using this form of ${G}_{1}^{(\ell )}$, it was shown that with the proper choice of *N*, ${G}_{1}^{(\ell )}=0$ if ${({\hat{\mathbf{n}}}_{0}\cdot {\hat{\mathbf{n}}}_{1})}^{(\ell )} < 0$. Finding the unit-pulse times that satisfies this condition for each target nuclear qubit is the first step in calibrating the parallel entangling gate. Furthermore, to quantify the multipartite entangling ability of a DD sequence with *L* target nuclear qubits, one can use the *M*-qubit entangling power:

10 $${\varepsilon }_{{\rm{p}},M}({U}_{\rm{DD}})={\left(\frac{d}{d+1}\right)}^{M}\mathop{\prod }\limits_{\ell }^{L}(1-{G}_{1}^{(\ell )}),$$

where *M* = *L* + 1 is the total number of qubits targeted for entangling, including the electron, and *d* = 2 is the dimension of the qubit subspace^41^. Often, as shown in Fig. 1, the normalized version of this metric is the most useful, without the constant coefficient in front of the product. The normalized metric ranges from 0 (the DD sequence creates no entanglement) to 1 (the DD sequence is a maximal multipartite entangler). Owing to the central spin nature of solid-state defect systems, *ε*_p,*M*_(*U*_DD_) depends only on each bipartite entanglement invariant ${G}_{1}^{(\ell )}$. Calculating *ε*_p,*M*_(*U*_DD_) with each of the target nuclear qubits in the range of unit-pulse times that satisfy ${({\hat{\mathbf{n}}}_{0}\cdot {\hat{\mathbf{n}}}_{1})}^{(\ell )} < 0$ reveals the optimum (*t*, *N*) combination to generate maximal multipartite entanglement.

We utilize the non-unitary entangling power to account for the impact of residual entanglement generated with non-targeted nuclear spins^41^. This entanglement metric is derived using the partial trace quantum channel ${\mathcal{E}}$ over non-targeted nuclear qubits. The set of all nuclear qubits is partitioned into a subset that is targeted (size *L*) and the rest that are not targeted (size, *L*_total_ − *L*). A simple approximate form for this entanglement metric is given by

11 $${\varepsilon }_{{\rm{p}},M}({\mathcal{E}})=\frac{{\varepsilon }_{{\rm{p}},M}({U}_{\rm{DD}})}{2}\left(1+\mathop{\prod }\limits_{{{\ell \in \,{\mathrm{not}}}\atop {\mathrm{targeted}}}}^{{L}_{{\rm{total}}}-L}{G}_{1}^{(\ell )}\right).$$

The non-unitary entangling power is bounded above by the unitary entangling power; ${\varepsilon }_{{\rm{p}},M}({\mathcal{E}})\le {\varepsilon }_{{\rm{p}},M}({U}_{\rm{DD}})$, with equality holding when no residual entanglement is generated (${G}_{1}^{(\ell )}=1$ for all non-targeted nuclear qubits)^41^. Therefore, any residual entanglement generated leads to a Makhlin invariant less than 1, lowering this entanglement metric. This metric is essential when designing parallel entangling gates with subsets of known nuclear qubits, for example, in the case of *L* = 2 parallel entangling gates in this work. Additional details regarding how these metrics were used to calibrate each parallel entangling gate are provided in Supplementary Section V.

### MQCs

In the original MQC circuit proposed in ref. ^43^ (Fig. 2a), the *M*-qubit register is first initialized to ${\left\vert 0\right\rangle }^{\otimes M}$. The control (top) qubit *q*_c_ is then placed into an equal superposition state so that the following CNOT gates create a GHZ state. Once entangled, each qubit’s relative phase is shifted by an equal amount *ϕ*, yielding $\left\vert {\,\mathrm{GHZ}\,}_{\phi }^{M}\right\rangle =\frac{1}{\sqrt{2}}({\left\vert 0\right\rangle }^{\otimes M}+{e}^{-iM\phi }{\left\vert 1\right\rangle }^{\otimes M})$. The system is then disentangled back to the original state by reversing the first half of the circuit. The result (before the last Hadamard that projects the control qubit phase onto the measurement axis) is that the control qubit’s phase is amplified based on how many qubits it was entangled with:

12 $$\left\vert {\psi }_{f}\right\rangle =\frac{1}{\sqrt{2}}(\left\vert 0\right\rangle +{e}^{-{\rm{i}}M\phi }\left\vert 1\right\rangle )\otimes {\left\vert 0\right\rangle }^{\otimes M-1}.$$

Thus, the final probability of the entire system returning to the initial state is given by

13 $$P\left({\left\vert 0\right\rangle }^{\otimes M}\right)=\frac{1}{2}(1+\cos (M\phi )),$$

which crucially carries a frequency equal to the number of qubits in the entangled state.

### Experimental MQC considerations

Nuclear phase gates *Z*_*ϕ*_ were implemented using off-resonant DD sequences. Since such gates are realized for any off-resonant *t*, the optimum parameters can be chosen strategically. The off-resonance region before the first-order resonances not only offers fast pulse times (*t* < 1 μs) but also can parallelize the phase gate, turning the sequence into an unconditional *M*-qubit gate. Experimentally, the finite pulse duration of electron gates sets a lower limit on *t*; this restriction, in turn, sets a lower bound on the angular resolution Δ*ϕ* = *ϕ*_*N*=1_ of the phase gate. With Δ*ϕ* specified, *t* was optimized to minimize the angular error for each nuclear qubit in the register. Then, to increase the phase, the unit pulse was repeated *N* times, leading to *ϕ* = *N*Δ*ϕ*. The simulated four-qubit process fidelities for a parallelized gate of the form ${I}_{e}\otimes {Z}_{\phi }^{\otimes 3}$ were ~99% for *ϕ* = π/2. Further details and a table of pulse parameters are provided in Supplementary Section V.

### Entangling-gate fidelities

*M*-qubit state fidelities are calculated according to the trace overlap of the quantum state *ρ* with the target state *ρ*_target_; *F*_*M*_ = tr(*ρ* × *ρ*_target_). On the basis of the form of the bipartite and sequential entangling gates, when *N*_E_ is a multiple of four, the target state is ${\left\vert 0\right\rangle }^{\otimes M}$—same as the initial state. The repeats of the parallel gate were chosen to maximize the overlap with ${\left\vert 0\right\rangle }^{\otimes M}$ as a target state. This simplifies the state fidelities to be given by only a single component of *ρ*; ${F}_{M}={\left\langle 0\right\vert }^{\otimes M}\rho {\left\vert 0\right\rangle }^{\otimes M}$. The fidelity of this separable target state can be further approximated by independent *Z*-axis measurements of each qubit:

14 $${F}_{M}\approx \frac{1}{{2}^{M}}\mathop{\prod }\limits_{\ell =1}^{M}(1+\langle {Z}_{\ell }\rangle ).$$

This approximation ignores correlations between qubits, which is reasonable since the initial and final states are separable with vanishing pairwise covariances and cumulants. Experimentally, the electron *Z*-axis projection is measured directly using spin-dependent fluorescence, whereas nuclear qubits are measured using *Z*-axis tomography (Fig. 3b). Supplementary Section X provides a derivation of equation (14) and additional details regarding the fidelity measurements.

### Generality and extensions

Random NV-nuclear-qubit registers were generated by uniformly sampling nuclear spin positions within a spherical volume surrounding an NV centre. The radius of this sphere was set to 2.3 nm, which encapsulates approximately 100 nuclear spins at natural abundance (1.1%). Nuclei at the surface of this sphere contribute to the spin bath. From the location of each nuclear qubit, the hyperfine matrix was calculated using the dipole–dipole interaction. The boundary between the strongly and weakly coupled qubits was set by the inhomogeneous linewidth of the electron-spin transitions, $\sqrt{2}/\pi {T}_{2}^{* }\approx 200$ kHz in this work. If a register contained any strongly coupled nuclear qubits, the case was not considered further (Fig. 5a, red region). Approximately 64% of the randomly generated registers contained at least one nuclear qubit with a hyperfine component larger than this cut-off. The remaining 36% of registers (Fig. 5a) were evaluated for parallel entanglement. We further applied lower bounds to separate addressable nuclei from the spin bath: ∣*A*_∥_∣ > 15 kHz (based on the location of the spin-bath resonance) and *A*_⊥_ > 10 kHz (so that a sufficiently small *N* can address the qubit). With these cut-offs, the average number of weakly coupled, addressable nuclear qubits per register is 5.7, with a standard deviation of 2.5 at natural ^13^C concentration.

For each of these registers, we searched for parallel entangling gates following the algorithm in ref. ^41^. Additional details are provided in Supplementary Sections V and VI. Because a large number of registers were generated to improve statistical significance, a conservative parallel entangling gate search was used. Specifically, a maximum of *N* ≤ 50 and a minimum non-unitary entangling power of ${\varepsilon }_{{\rm{p}},M}({\mathcal{E}})\ge 0.8$ were imposed. Hence, these results represent a lower bound on the available gates. Supplementary Section VI provides additional simulations of gate durations, comparisons with *k* = 2 and *k* = 3 sequential two-qubit gates, and the infidelity arising from residual entanglement.

## Online content

Any methods, additional references, Nature Portfolio reporting summaries, source data, extended data, supplementary information, acknowledgements, peer review information; details of author contributions and competing interests; and statements of data and code availability are available at https://doi.org/10.1038/s41565-026-02254-6.

## Supplementary information

Supplementary Information Supplementary Sections I–XI, Figs. 1–26, Tables 1–11 and Discussion.

Peer Review File

## Data Availability

The data associated with this study, including the raw and processed data in the main-text figures, are available via Zenodo at https://doi.org/10.5281/zenodo.20969058 (ref. ^51^).

## Code availability

The code used to process and plot the data in this study is available via Zenodo at https://doi.org/10.5281/zenodo.20970922 (ref. ^52^) and via GitHub at https://github.com/penn-qel/parallel_entanglement.
