# Supplementary material for: Single-gate, multipartite entanglement on a room-temperature quantum register
Source: Nat Nanotechnol. 2026 Sep 14;21(10):1383–9. doi: 10.1038/s41565-026-02254-6 (PMC13661294; doi:10.1038/s41565-026-02254-6)
Supplement: Supplementary file 1 [file 41565_2026_2254_MOESM1_ESM.pdf]

nature nanotechnology

Supplementary information https://doi.org/10.1038/s41565-026-02254-6

Single-gate, multipartite entanglement on a room-temperature quantum register

In the format provided by the authors and unedited

CONTENTS

I. Experimental Control 2

II. Electronic qubit 2
A. Control 2
B. Pulse bootstrap tomography 3
C. Photoluminescence calibration 4

III. System Hamiltonian 4
A. Dynamical decoupling 7

IV. Detection of nearest ^{13}C qubits 9

V. Nuclear gates 10
A. Calibration 10
B. Parallel Z gates 11
C. Parallel entanglement 12

VI. Generality and extensions 13

VII. Magnetic field optimization 18
A. Alignment 18
B. Field strength 18

VIII. Individual nuclear qubit control circuits 19
A. Nuclear qubit initialization and tomography 19
B. Experimental nuclear gate calibration 20

IX. Multiple Quantum Coherences 20
A. Idealized theory 20
B. Circuit optimization 23
C. Residual entanglement effects on MQC signal 25
D. Additional data 27

X. Entangling gate fidelities 27
A. State fidelity expression 27
B. Entangling gate repeat sampling 30
1. Bipartite and sequential gates 30
2. Parallel gates 30
C. Kraus operator error model 31

XI. Applications to quantum error correction 32

References 34

I. EXPERIMENTAL CONTROL

Optical and MW control synchronization was handled using two distinct configurations, each of which provided data for this work. In the first configuration (AWG configuration of Fig. I), two arbitrary waveform generators (AWG) were used to handle the MW and optical control separately, coordinating control stages via handshaking. The lead AWG (AWG520, Tektronix) controlled an acousto-optic modulator (AOM) (1250C-848, ISOMET) to modulate a green laser, routed photon counts from an avalanche photodiode (APD) (COUNT-T100 SPCM, Laser Components) to the data acquisition (DAQ) unit (PCIe-6323, National Instruments) using a switch (ZYSWA-2-50DR, Mini-Circuits), and acted as an external clock to the DAQ. After electron initialization, the higher time resolution (typically 1ns sampling) MW control AWG (AWG7102, Tektronix) was triggered. The two analog outputs of this AWG controlled the IQ modulation of a signal generator (SG384, Stanford Research Systems, 750-4050MHz LO bandwidth) for phase control of the MWs. The signal generator output fed into a high bandwidth mixer (ZX05-63LH+, Mini-Circuits) for fast amplitude modulation (controlled by an AWG digital channel). For high isolation during periods without MWs, the signal then fed into a high isolation switch (ZASWA-2-50DRA+, Mini-Circuits) to prevent leakage. From there, a fixed amplifier (ZHL-15W-422-S+, Mini-Circuits) and variable attenuator (Rudat 6000-60, Mini-Circuits) were used to control the power of the MW signal before reaching the sample. Signals reached the NV using a custom SMA-connected PCB with wire bond traces to the antenna around the solid immersion lens. At the end of MW control, the AWG7102 triggered the AWG520 to perform optical readout of, or reinitialize, the electron.

In the second control configuration (Pulse Streamer configuration of Fig. I), rather than using two AWGs to separately control optical and MW stages, a single AWG (Pulse Streamer 8/2, Swabian) was used to control all non-AWG equipment discussed above. The Pulse Streamer's analog channels have lower bandwidth (125MHz) than the AWG7102, so to prevent ringing when using square pulses, Gaussian rising and falling edges were optimized and applied before and after setting a constant voltage output. Once the constant voltage level was reached, the mixer was used to amplitude modulate the output of the signal generator, providing clean rising and falling edges to the pulse. Digital channels of the Pulse Streamer can be modulated with 1ns resolution, comparable to the sampling of the AWG7102 used.

The AWG configuration provided the data shown in Fig. 1 and 2 of the main text, while the Pulse Streamer configuration provided data shown in Fig. 3 and 4. All of the data shown in this supplement was taken with the AWG configuration, except for Sec. II A, IX and X.

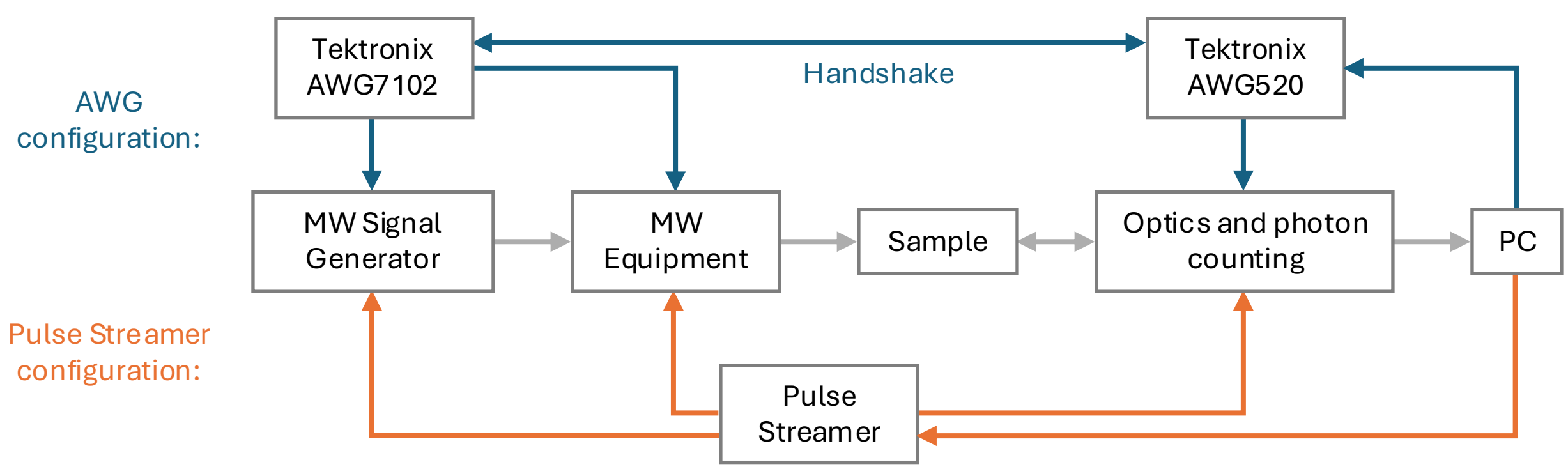

FIG. 1. **Experimental control configurations** Equipment in the middle is common to both configurations. In both configurations, the two analog channels of either the AWG7102 or the Pulse Streamer were connected to the IQ modulation of the MW signal generator. The "MW equipment" block contains the mixer, high isolation switch, amplifier and attenuator. The "Optics and photon counting" block contains the APD, switch and DAQ. The PC block contains a very tired PhD student.

II. ELECTRONIC QUBIT

A. Control

The logical states of the spin 1 electronic qubit were chosen to be $|0\rangle = |m_s = 0\rangle$ and $|1\rangle = |m_s = -1\rangle$. To calibrate the transition frequency between these spin states, a pulsed electron spin resonance (ESR) experiment (Fig. 2a) was used. The frequency range swept was first estimated by the Zeeman splitting of the permanent magnet relative to the NV zero field splitting of ≈ 2.8GHz. The carrier frequency of the signal generator was detuned using IQ modulation

where a low-power 1μs MW pulse was supplied at each frequency. The sample data set in Fig. 2a shows the expected three resonances for each electron spin transition due to the strong 2.2MHz hyperfine interaction with the unpolarized, nuclear spin 1, Nitrogen 14 neighbor.

From the resonance locations fitted in pulsed ESR, the center one (Nitrogen spin state $|m_s = 0\rangle$) is used as the carrier frequency in all subsequent experiments. To calibrate pulse duration, standard resonant Rabi oscillations of the electron were measured using higher power MWs (Fig. 2b). Typical Rabi frequencies used were around 150MHz for the AWG configuration and 100MHz for the Pulse Streamer configuration. From the Rabi fitting results, the pulse durations needed for a $\pi/2$ and π rotation were calculated and used for further experiments. Due to the high power MWs used, direct time-dependent simulations showed nearly no effect from the unpolarized Nitrogen 14 spin states, which can act as further frequency detuning during Rabi oscillations. In lower power regimes, this detuning can prevent the electron from making full oscillations about its Bloch sphere and thus affect the gate fidelities achieved based on the pulse durations calibrated.

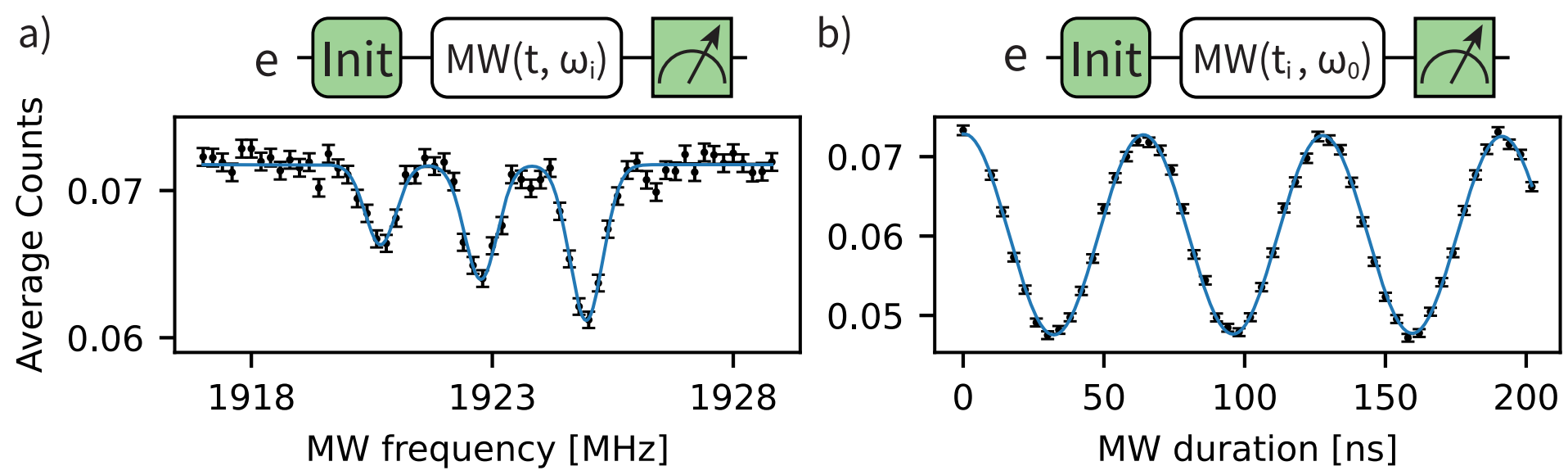

FIG. 2. **Standard electron control calibration (a)** Pulsed electron spin resonance experiment diagram for frequency calibration and example results. Splittings between the resonances match the expected Nitrogen 14 interaction strength of 2.2MHz. **(b)** Rabi oscillation experiment diagram for gate duration calibration and example results. Very short pulse durations (less than 10ns) were omitted due to the limited rise and fall times of the hardware. Both of these experiments were executed periodically to update electron gate calibration and mitigate drift over time. Data points denote mean values taken of $3 \cdot 10^5$ and 10^5 shots, respectively, and error bars denote photon shot noise. Solid lines denote fits to extract resonant frequencies and Rabi frequency.

B. Pulse bootstrap tomography

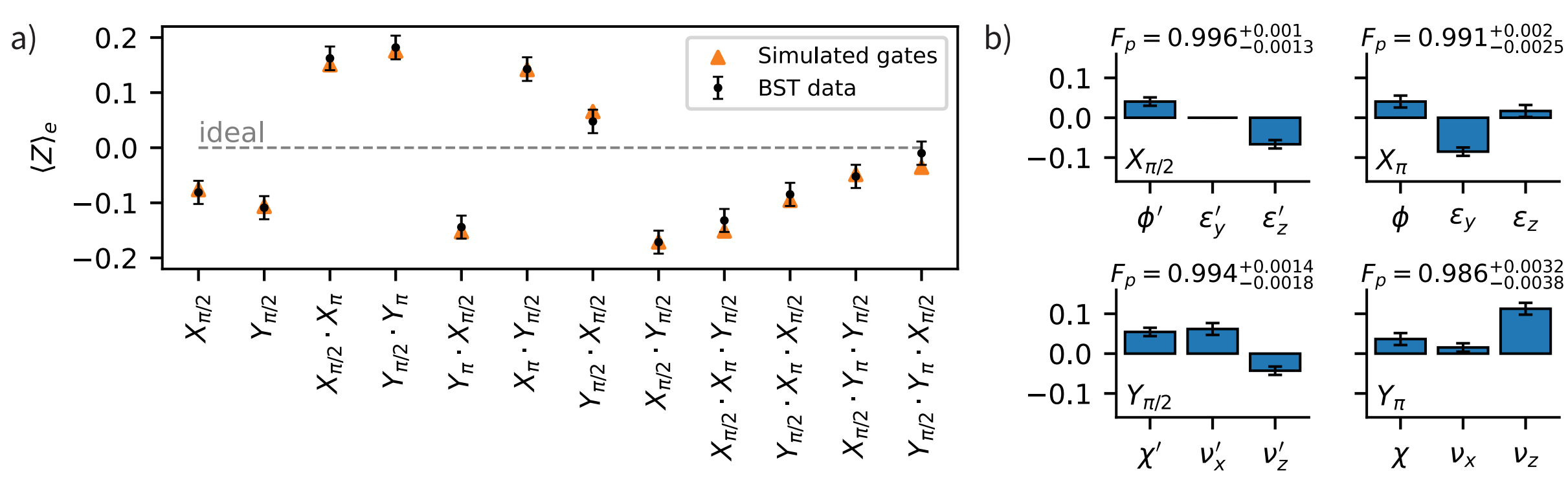

FIG. 3. **Electron control pulse errors (a)** Results of each pulse sequence needed for error parameter calculations. Each sequence provides a linear equation for the error parameters based on the gates used, which can then be solved to extract them. Data points denote mean values taken over 10^6 shots and error bars denote photon shot noise. **(b)** Gate errors measured from sequences in (a). Variable convention follows [1] where ϕ, χ are the angular errors and ε_i, ν_i are the off-axis errors. Each gate's process fidelity F_p is shown in the title. Based on errors extracted, each sequence was then simulated (orange triangular points in (a)) to confirm analysis, as shown by the agreement with the original data in (a). Error bars and uncertainties denote propagated errors from (a).

Pulse bootstrap tomography (BST) was used to quantify coherent errors in the electron control pulses [1]. This protocol quantifies the angular and off-axis errors present in all electron pulses used in this work: $X_{\pi/2}, X_\pi, Y_{\pi/2}$ and

Y_π. Gate errors are extracted by using multiple sequences of pulses, each of which creates a linear combination of the error parameters. The sequences (x axis of Fig. 3a) together generate a system of equations that yield each gate's error parameters. The results (black points with error bars) of each pulse sequence required for this method are shown in Fig. 3a and the calculated gate error parameters are shown in Fig. 3b, following the parametrization in [1]. For example, the imperfect X_π gate is parameterized as:

$$X_\pi = \exp\big(- i\hat{\varepsilon}\cdot\vec{\sigma}(\pi+2\phi)/2\big), \tag{1}$$

$$\hat{\varepsilon} = \big(\sqrt{1-\varepsilon_y^2-\varepsilon_z^2}, \varepsilon_y, \varepsilon_z\big). \tag{2}$$

Based on the results of Fig. 3b, imperfect unitary operators were constructed and the sequences of Fig. 3a were simulated (orange triangular points) to verify correct analysis of Fig. 3a results. Additionally, each gate's process fidelity was calculated using these imperfect gates (Fig. 3b). Most importantly, these imperfect gates were used in all subsequent gate-based simulations shown. Despite all pulses showing approximately 99% fidelity, one could further mitigate errors through various technique like fine-tuning the pulse voltage for each of the four pulse types to individually adjust the Rabi frequency, or pulse shaping [2]. However, the small errors present were enough to achieve all desired experiments with XY8 dynamical decoupling sequences further mitigating errors (see Sec. III A).

C. Photoluminescence calibration

In order to calibrate the photoluminescence (PL) levels of each electron spin state, two calibration AWG lines were used with every repeat of a PL-calibrated experiment. The first line used no MW control, thus measuring the PL of the initialized $|0\rangle$ state, and the second line applied a calibrated π pulse to measure the PL level of $|1\rangle$. Adiabatic passage of the electron from $|0\rangle \rightarrow |1\rangle$ in the presence of the Nitrogen 14 detuning could have been used as well [3]. However, at high MW powers, this influence was minimal as discussed in Sec. II A and also in the supplement of [1].

We observed an unexpected increase in PL during $\approx$10μs and greater experimental durations. To better understand this behavior, we added a variable delay before measurement of each calibration line (no pulse and π-pulse) (Fig. 4a,b). The reference PL levels increase approximately equally for both spin states, with the steady state being roughly 15% larger than immediate calibration (no delay) depending on the laser power used. This unexpected behavior presented issues with longer experiments involving nuclear qubits that were greater than $\approx$10μs — the PL from no-delay calibration would consistently indicate larger than 1 probabilities. Therefore, all PL calibrations used in this work were delayed by 20μs to be approximately within the steady state region without considerably increasing overall experimental durations. These issues also complicated the use of adiabatic passage, since that method involves longer experimental sequences and the exponentially flat region used to calibrate $|1\rangle$ began to increase, affecting calibration values. A similar delay could be added prior to an adiabatic passage in future work, but the high power π pulse was accurate enough for our purposes.

III. SYSTEM HAMILTONIAN

Consider a central electronic spin surrounded by L nuclear spins in the presence of a static magnetic field B in the z direction. Inter-nuclear spin-spin interactions are neglected here due to their much weaker strength than electron-nuclear interactions. Furthermore, neglecting non-secular terms of the spin-spin interaction tensor, and in the Larmor frame of the electron, the Hamiltonian governing this spin system is given by:

$$H = \mathbb{1}\otimes\frac{\omega_{\rm Lar}}{2}\sum_{\ell=1}^{L}\sigma_z^{(\ell)} + \frac{Z_e}{2}\otimes\sum_{\ell=1}^{L}(A_{||}^{(\ell)}\sigma_z^{(\ell)} + A_{\perp}^{(\ell)}\sigma_x^{(\ell)}), \tag{3}$$

where $\omega_{\rm Lar} = \gamma_{\rm nuc}B$ is the nuclear spin Larmor frequency, $A_{||,\perp}^{(\ell)}$ are the parallel and perpendicular components of the hyperfine spin-spin tensor of the ℓth nuclear spin, and Z_e is the spin operator acting on the electronic two-level subsystem. It is defined as:

$$Z_e = s_0\,|0\rangle\langle 0| + s_1\,|1\rangle\langle 1|\,, \tag{4}$$

where the logical qubit states $|0\rangle$ and $|1\rangle$ are chosen from the spin-1 triplet as $|0\rangle = |m_s = 0\rangle$ and $|1\rangle = |m_s = -1\rangle$, as is common in NV center systems. This choice set $s_0 = 0$ and $s_1 = -1$. Each Pauli operator $\sigma_i^{(\ell)}$ acts only on the

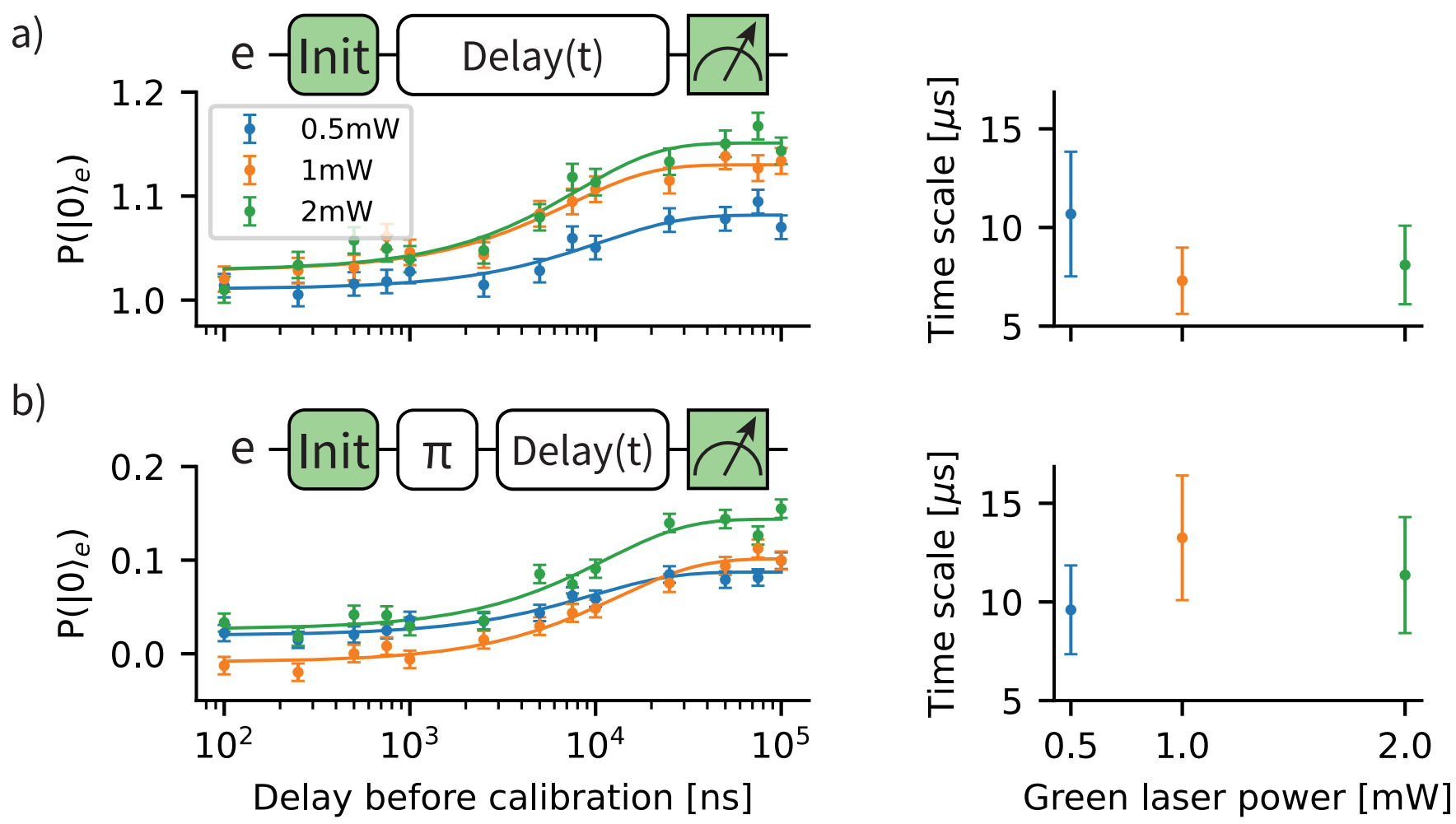

FIG. 4. **Delayed PL calibration (a,b)** Pulse sequence and results of measuring the time scale of the PL increase when calibrating the $|0\rangle$, $|1\rangle$ states of the electron, respectively. Laser power slightly affects the steady state reached, but not the fitted time scale of the increase. Data points on left plots denote mean values taken over 10^6 shots and error bars denote photon shot noise. Error bars on right plots denote 68% confidence interval from fits.

ℓth nuclear spin and is defined as the tensor product of identity operators with a single σ_i on the ℓth factor:

$$\sigma_i^{(\ell)} = \mathbb{1} \otimes \mathbb{1} \otimes \underbrace{...\sigma_i...}_{\substack{\ell\text{th}\\ \text{factor}}} \otimes \mathbb{1}. \tag{5}$$

In principle, the Nitrogen 14 spin can be considered in this Hamiltonian. However in this work, we restrict our attention to the L surrounding Carbon 13 spins. The Nitrogen 14 spin strongly couples to the electron with $A_{||} \approx 2.2$ MHz, while the nearest Carbon 13 spins weakly couple, with both parallel and perpendicular components in the range 10 to 100 kHz. No strongly coupled Carbon 13 spins were located around the NV studied. Since our focus is on the dynamics of the Carbon 13 spins using dynamical decoupling, we can safely assume that the electron is effectively decoupled from the Nitrogen 14 spin in the parameter regions of interest. Therefore, L will refer to the number of Carbon 13 spins, referred to as the "nuclear qubits" from now on, being considered in the register.

The Hamiltonian of equation (3) can be simplified to the form [4]:

$$H = \sum_{j\in\{0,1\}} |j\rangle\langle j| \otimes \sum_{\ell}^{L} H_j^{(\ell)}, \tag{6}$$

where each $H_j^{(\ell)}$ is given by:

$$H_j^{(\ell)} = \frac{\omega_{\text{Lar}} + s_j A_{||}^{(\ell)}}{2} \sigma_z^{(\ell)} + \frac{s_j A_{\perp}^{(\ell)}}{2} \sigma_x^{(\ell)}. \tag{7}$$

This form of the Hamiltonian facilitates the derivation of the free evolution operator $U_f(t)$:

$$U_f(t) = \exp\Bigg(-it \sum_{j\in\{0,1\}} |j\rangle\langle j| \otimes \sum_{\ell}^{L} H_j^{(\ell)} \Bigg). \tag{8}$$

The two terms in the sum over electronic spin states $|j\rangle$ commute with one another, and thus the exponential factorizes:

$$U_f(t) = \exp\Bigg(-it\,|0\rangle\langle 0| \otimes \sum_{\ell}^{L} H_0^{(\ell)} \Bigg) \exp\Bigg(-it\,|1\rangle\langle 1| \otimes \sum_{\ell}^{L} H_1^{(\ell)} \Bigg). \tag{9}$$

For any finite dimensional Hermitian operator A, the operator $\exp(-it\,|j\rangle\langle j| \otimes A)$ has a alternative form given by:

$$\exp(-it\,|j\rangle\langle j| \otimes A) = |\bar{j}\rangle\langle\bar{j}| \otimes \mathbb{1} + |j\rangle\langle j| \otimes \exp(-itA), \tag{10}$$

where the barred projection operator is defined as $|\bar{j}\rangle\langle\bar{j}| = \mathbb{1} - |j\rangle\langle j|$. In the two-dimensional case, these are just $|\bar{0}\rangle\langle\bar{0}| = |1\rangle\langle 1|$ and $|\bar{1}\rangle\langle\bar{1}| = |0\rangle\langle 0|$. The form of equation (10) is more convenient to work with because the tensor product between the electron and the nuclear qubits is outside of the exponential. Applying equation (10) to equation (9) yields:

$$\begin{aligned} U_f(t) &= \left(|1\rangle\langle 1| \otimes \mathbb{1} + |0\rangle\langle 0| \otimes \exp\left(-it\sum_{\ell}^{L} H_0^{(\ell)}\right)\right)\left(|0\rangle\langle 0| \otimes \mathbb{1} + |1\rangle\langle 1| \otimes \exp\left(-it\sum_{\ell}^{L} H_1^{(\ell)}\right)\right) \\ &= |0\rangle\langle 0| \otimes \exp\left(-it\sum_{\ell}^{L} H_0^{(\ell)}\right) + |1\rangle\langle 1| \otimes \exp\left(-it\sum_{\ell}^{L} H_1^{(\ell)}\right) \\ &= \sum_{j\in\{0,1\}} |j\rangle\langle j| \otimes \exp\left(-it\sum_{\ell}^{L} H_j^{(\ell)}\right), \end{aligned} \tag{11}$$

which shows that the electron projection operators factor out of the exponential in equation (8). Furthermore, each of the nuclear qubit sub-Hamiltonians $H_j^{(\ell)}$ commute for different nuclear qubit subspaces:

$$[H_j^{(\ell)}, H_j^{(k)}] = 0, \tag{12}$$

which again allows the exponential in equation (11) to factorize:

$$U_f(t) = \sum_{j\in\{0,1\}} |j\rangle\langle j| \otimes \prod_{\ell}^{L} \exp\left(-itH_j^{(\ell)}\right). \tag{13}$$

Recall that $H_j^{(\ell)}$ is defined as the $2^L \times 2^L$-dimensional operator with the identity in all components except the ℓth. Because of this simple form, the identities factor down from the exponential and we are left with only an exponential of a 2×2 operator $H_j^{(\ell)}$ (no identities implied) for each nuclear qubit. Thus at this point, the index notation of ℓ will simply denote each nuclear qubit's 2×2 sub-Hamiltonian, without implied identity operators. In this shift in notation and the simplification described, we are left with:

$$U_f(t) = \sum_{j\in\{0,1\}} |j\rangle\langle j| \bigotimes_{\ell}^{L} \exp\left(-itH_j^{(\ell)}\right), \tag{14}$$

showing how each ℓth subspace of the Hilbert space transforms individually based on the electron state $|j\rangle$.

To better understand the nuclear qubit transformations, define two normalized vectors $\hat{p}_j^{(\ell)}$ for each nuclear qubit dotted into the Pauli vector $\vec{\sigma} = (\sigma_x, \sigma_y, \sigma_z)$ based on the form of $H_j^{(\ell)}$ in equation (7). This can be done for general electron spin projections and then later specified to the logical basis often used:

$$\hat{p}_j^{(\ell)} = (s_j A_\perp^{(\ell)}, 0, \omega_{\text{Lar}} + s_j A_{||}^{(\ell)})/\omega_j^{(\ell)}, \tag{15}$$

where the normalization factor $\omega_j^{(\ell)}$ is the magnitude of the vector $\vec{p}_j^{\,(\ell)}$:

$$\omega_j^{(\ell)} \equiv |\vec{p}_j^{\,(\ell)}| = \sqrt{(s_j A_\perp^{(\ell)})^2 + (\omega_{\text{Lar}} + s_j A_{||}^{(\ell)})^2}. \tag{16}$$

With this definition, each $H_j^{(\ell)}$ can be expressed as:

$$H_j^{(\ell)} = \omega_j^{(\ell)} \hat{p}_j^{(\ell)} \cdot \vec{\sigma}/2. \tag{17}$$

Thus, each nuclear qubit operator in equation (14) is given by:

$$\begin{aligned} \exp\left(-itH_j^{(\ell)}\right) &= \exp\left(-i(t\omega_j^{(\ell)})\hat{p}_j^{(\ell)} \cdot \vec{\sigma}/2\right) \\ &= R_{\hat{p}_j^{(\ell)}}(\theta_j^{(\ell)}), \end{aligned} \tag{18}$$

which is a spin 1/2 rotation operation with rotation axis given by equation (15) and angle $\theta_j^{(\ell)} = t\omega_j^{(\ell)}$. Substituting this into equation (14) gives the most intuitive form of free evolution for this system:

$$U_f(t) = \sum_{j\in\{0,1\}} |j\rangle\langle j| \bigotimes_{\ell}^{L} R_{\hat{p}_j^{(\ell)}}(\theta_j^{(\ell)}). \tag{19}$$

Equation (19) shows that the spin state of the electron $|j\rangle$ conditions how each nuclear qubit evolves around its respective Bloch sphere. Now consider the common choice of electron spin states $|0\rangle = |m_s = 0\rangle$ and $|1\rangle = |m_s = -1\rangle$. If the electron is in state $|0\rangle$, nuclear qubit evolution simply obeys Larmor precession about the $\hat{z}$ axis of the magnetic field. However, if the electron spin is in state $|1\rangle$, it acts as a small change in the local magnetic field of each nuclear qubit. This leads to a unique precession axis and rate for each nuclear qubit based on equations (15) and (16). In the case of free evolution, $\hat{p}_0^{(\ell)}$ and $\hat{p}_1^{(\ell)}$ are nearly aligned and importantly fixed by the strength of the hyperfine coupling of the ℓth nuclear qubit. Yet the non-zero angle between $\hat{p}_0^{(\ell)}$ and $\hat{p}_1^{(\ell)}$ allows for dynamical decoupling of the electron to engineer controllable rotations of the nuclear qubits about desired axes, as discussed in the following section.

A. Dynamical decoupling

Dynamical decoupling (DD) refers to the process of switching the electron state periodically to achieve two main outcomes. First, DD can be used to engineer controllable interactions with nuclear qubits (*i.e.*, implement nuclear gates). Second, the process of repeatedly flipping the electronic state averages out its interactions with the larger spin environment, thereby decoupling it from unwanted noise [5]. This technique is essential for extending the electron coherence time beyond its short dephasing T_2^* time. At room temperature, this is often a difference of three orders of magnitude from μs to ms. In what follows, we focus our attention on dynamical decoupling as a tool of sensing and controlling nuclear qubits. Between electron flips, the system undergoes free evolution according to $U_f(t)$ in equation (19). By carefully controlling the time between flips and the total amount of them, one can realize universal nuclear qubit control.

The simplest DD sequence is commonly known as CPMG (Carr-Purcell-Meiboom-Gill), with a unit pulse sequence consisting of two π-pulses with even spaced delays and about the same axis. The timing conventions used throughout this work is that the total CPMG unit pulse takes a duration t. This unit pulse is then repeated a total of N times to achieve a particular outcome with the nuclear qubits. Schematically, the pulse sequence is given by:

$$\text{CPMG} = \left(\frac{t}{4} - \pi - \frac{t}{2} - \pi - \frac{t}{4}\right)^N, \tag{20}$$

where each π denotes a π-pulse about either the X or Y axis.

First, consider the effect of a single unit pulse ($N = 1$) with π-pulses about the X axis. Using equation (19), the total unitary operator is given by:

$$U_{\text{CPMG}} = U_f(t/4)\cdot X_\pi \cdot U_f(t/2)\cdot X_\pi \cdot U_f(t/4). \tag{21}$$

We only need to consider how $X_\pi U_f(t/2) X_\pi$ transforms the electronic subspace since it acts as the identity on all nuclear qubits. This transformation simply swaps the projectors:

$$\begin{aligned} X_\pi \cdot U_f(t/2)\cdot X_\pi &= |1\rangle\langle 1| \bigotimes_{\ell}^{L} R_{\hat{p}_0^{(\ell)}}(\theta_0^{(\ell)}(t/2)) + |0\rangle\langle 0| \bigotimes_{\ell}^{L} R_{\hat{p}_1^{(\ell)}}(\theta_1^{(\ell)}(t/2)) \\ &\equiv \bar{U}_f(t/2). \end{aligned} \tag{22}$$

This simplifies which terms appear in the product of $U_{CPMG} = U_f(t/4)\bar{U}_f(t/2)U_f(t/4)$. The resulting expression, which matches the analysis of [4], leads to a net unitary of very similar form to free evolution:

$$U_{\text{CPMG}}(t) = \sum_{j\in\{0,1\}} |j\rangle\langle j| \bigotimes_{\ell}^{L} R_{\hat{n}_j^{(\ell)}}(\phi^{(\ell)}), \tag{23}$$

however, these rotation operators are different than the ones given in the free evolution analysis. Using the shorthand $R_{\hat{p}_j^{(\ell)}}(\theta_j^{(\ell)}(t)) = R_j^{(\ell)}(t)$ for the free evolution rotations, the net DD rotations are given by:

$$R_{\hat{n}_j^{(\ell)}}(\phi^{(\ell)}) = \begin{cases} R_0^{(\ell)}(t/4)R_1^{(\ell)}(t/2)R_0^{(\ell)}(t/4) \text{ for } j = 0, \\ R_1^{(\ell)}(t/4)R_0^{(\ell)}(t/2)R_1^{(\ell)}(t/4) \text{ for } j = 1 \end{cases} \tag{24}$$

The DD nuclear rotation angles $\phi^{(\ell)}$ are electron spin state independent, unlike the case of free evolution [4]. This allows for both the unit pulse times and repeats (next paragraph) to be calibrated independent of the electron state.

Repeating the unit pulse N times can now be analyzed directly. Using induction, one can show that the Nth power of equation (23) increases the nuclear rotations angles linearly:

$$U_{\text{CPMG}}^{N}(t) = \sum_{j\in\{0,1\}} |j\rangle\langle j| \bigotimes_{\ell}^{L} R_{\hat{n}_j^{(\ell)}}(N\phi^{(\ell)}). \tag{25}$$

Therefore, the choice of N sets the net nuclear rotation angle. On the other hand, the choice of unit pulse time sets the rotation axes and the angular resolution $\Delta\phi = \phi^{(\ell)}$. Most often, the unit pulse time is chosen to be on resonance with a particular nuclear qubit's free precession. In doing so, the two electron spin dependent rotation axes either take a parallel $(\hat{n}_0 \cdot \hat{n}_1)_\ell = 1$ or antiparallel $(\hat{n}_0 \cdot \hat{n}_1)_\ell = -1$ alignment. This is in stark contrast to the small, but nonzero angle between free evolution rotation axes $\hat{p}_j^{(\ell)}$, which is fixed by the physical parameters of the system. Parallel rotation axes correspond to nuclear rotations that are independent of the electron state (unconditional gate), while anti-parallel axes generate operations that are maximally dependent on the electron state. In the anti-parallel case, this leads to an entangling gate between the qubits, which can be maximally entangling with the proper choice of N. Details of these different types of rotations will be discussed more in Sec. V A. Together, they enable dynamical decoupling to be a form of universal control for nuclear qubits [6].

Using the form of equation (23), [7] derived a closed form of $(\hat{n}_0 \cdot \hat{n}_1)_\ell$ for the common choice of spin projections in the NV system, which was recently generalized in [4]. In either case, analyzing $(\hat{n}_0 \cdot \hat{n}_1)_\ell$ as a function of unit pulse time allows one to derive an expression for which unit pulse times give rise to these resonant, (anti)parallel axis geometries. However, the resulting equation is transcendental and cannot be solved analytically [7]. Under the approximation of a strong magnetic field relative to the hyperfine couplings ($\omega_{\text{Lar}} \gg A_{||,\perp}^{(\ell)}$), an approximate resonance condition equation can be derived:

$$t_m^{(\ell)} = \frac{4\pi m}{\omega_0^{(\ell)} + \omega_1^{(\ell)}} \text{ for } m \in \mathbb{Z}, \tag{26}$$

where $\omega_j^{(\ell)}$ is defined in equation (16). The parity of m gives rise to parallel or antiparallel nuclear rotation axes:

$$(\hat{n}_0 \cdot \hat{n}_1)_\ell(t_m^{(\ell)}) = \begin{cases} -1 \text{ for odd } m \to m = 2k-1, \\ +1 \text{ for even } m \to m = 2k \end{cases} \tag{27}$$

because of this, the choice of resonance will always be quoted as a value of k starting at 1 and the context of the resonance (conditional or unconditional) can be determined from the context.

Although the analysis considered thus far has analyzed CPMG DD, this form of DD is often not viable experimentally. Due to small pulse errors in the electron π-pulses, the error accumulates after many applications. Alternative DD sequences have been developed to cancel these pulse errors to leading order, preserving the state of the electron [8]. To this end, XY8 DD was predominantly used throughout this work. The unit pulse for this sequence has four times as many π-pulses, of which the axes of each rotation is chosen symmetrically:

$$\text{XY8} = \frac{t}{4} - X_\pi - \frac{t}{2} - Y_\pi - \frac{t}{2} - X_\pi - \frac{t}{2} - Y_\pi - \frac{t}{2} - Y_\pi - \frac{t}{2} - X_\pi - \frac{t}{2} - Y_\pi - \frac{t}{2} - X_\pi - \frac{t}{4}. \tag{28}$$

The convention of timing chosen for this work defines the XY8 unit pulse to be four times longer than the CPMG unit pulse. Therefore, all unit pulse times always have the meaning of $t/2$ between successive π-pulses, except for the first and last delay period of $t/4$, and N unit pulse repeats corresponds to $2N$ π-pulses in total. Importantly, all results derived for CPMG apply equally to XY8.

When using only the XY8 unit pulse, it can be difficult to achieve particular nuclear qubit rotations with high accuracy due to the discrete nature of angle accumulation through unit pulse repeats. Therefore, in order to strike a balance between electron and nuclear rotation errors, multiple schemes of DD were applied using as much XY8 as possible. To interpolate between multiples of four repeats, DD schemes with smaller unit pulses were used. This included CPMG, XY4 and XY6 in the order of $N \equiv 1, 2, 3 (\text{mod} 4)$. Symmetrization beyond XY8 did not show an improvement in preserving the electron state. Interpolating with these smaller unit pulses allowed for more precise nuclear qubit rotation operators to be achieved, as discussed more in Sec. V A.

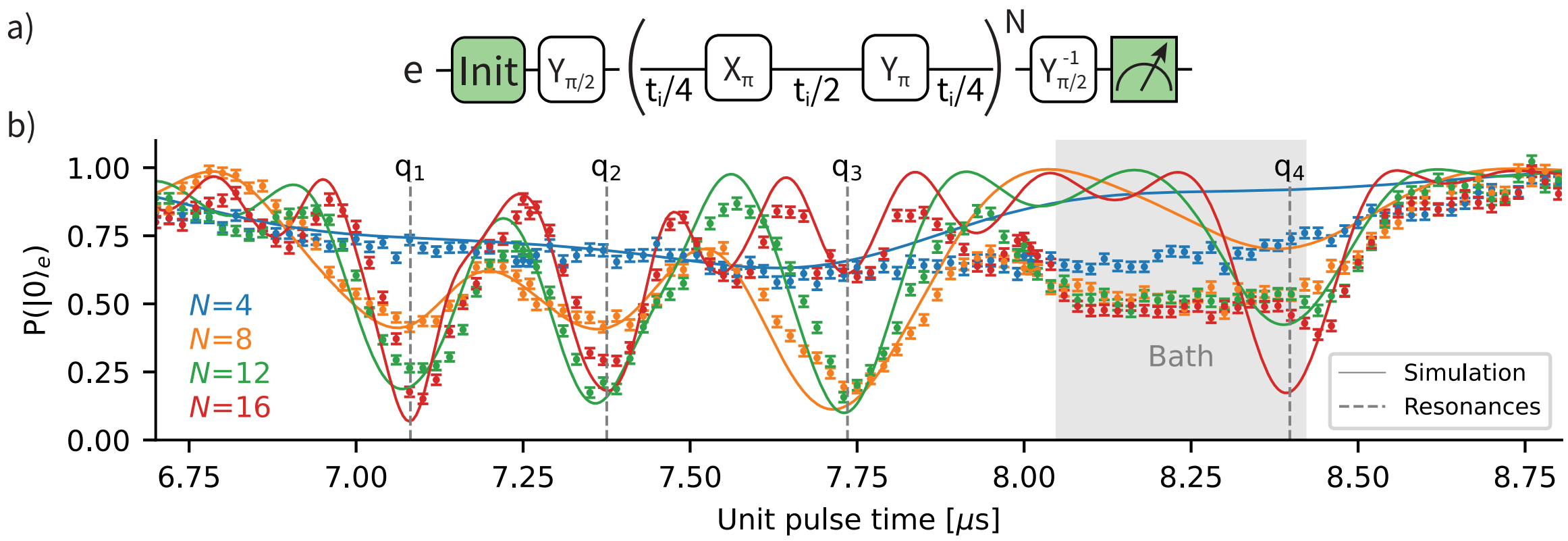

FIG. 5. **Second order** ($k = 2$) **DD spectroscopy (a)** Schematic of the pulse sequence used, with the XY8 unit pulse abbreviated. Placing the electron in an equatorial state leads the following variable DD sequence to entangle nearby nuclear qubits in their thermal states. This entanglement creates off diagonal elements of the electron density matrix, which are mapped onto the final state with the final $Y^{-1}_{\pi/2}$ pulse. **(b)** Second order resonance region of four distinct nuclear qubits. Dashed lines denote resonance unit pulse times based on equation (26). Note, the simulation is not fit to the data. Data points denote mean values taken over 10^6 shots and error bars denote photon shot noise.

IV. DETECTION OF NEAREST ^{13}C QUBITS

DD spectroscopy [7] was used to sense the spin environment and determine the hyperfine parameters $A_{||,\perp}$ of nearby ^{13}C nuclear qubits (Fig. 5). This experiment works by entangling the electron with the nuclear qubits in uninitialized thermal states. This entanglement reduces the probability of finding the electron in its initial state, producing a resonance when entanglement is achieved. The unit pulse times that lead to these resonances are given by equation (26) for odd m. This probability as a function of unit pulse time can be derived by considering an initial density matrix of the form:

$$\begin{aligned} \rho_0 &= |X\rangle\langle X| \otimes \left(\frac{1}{2}I\right)^{\otimes L} \\ &= \frac{1}{2^{L+1}}\Big(|0\rangle\langle 0| \otimes I^{\otimes L} + |1\rangle\langle 1| \otimes I^{\otimes L} + |0\rangle\langle 1| \otimes I^{\otimes L} + \text{h.c.}\Big) \end{aligned} \tag{29}$$

after the first $\pi/2$ pulse is applied to the electron. Using the unitary operator of equation (23), applying the DD sequence to the system results in:

$$U_{DD}\rho_0 U_{DD}^{\dagger} = \frac{1}{2^{L+1}}\Big(|0\rangle\langle 0| \otimes I^{\otimes L} + |1\rangle\langle 1| \otimes I^{\otimes L} + |0\rangle\langle 1| \bigotimes_{\ell}^{L} R_{\hat{n}_0^{(\ell)}} R^{\dagger}_{\hat{n}_1^{(\ell)}} + \text{h.c.}\Big), \tag{30}$$

which has created a variable off-diagonal element for the electron that depends on the conditional nature of the nuclear qubit rotation operators $R_{\hat{n}_0^{(\ell)}}$ and $R_{\hat{n}_1^{(\ell)}}$. When these are unconditional, or non-entangling, we have $R_{\hat{n}_0^{(\ell)}} R^{\dagger}_{\hat{n}_1^{(\ell)}} = \mathbb{1}$ for each ℓ Therefore, the off diagonal element is unchanged and the probability of the electron remaining in the state $|X\rangle$ is just 1. However, for conditional operators, tracing out the nuclear qubits and measuring in the X basis (equivalent to applying the final $\pi/2$-pulse) leads to:

$$\begin{aligned} \rho_e &= \text{tr}_{\ell}\, \rho \\ &= \frac{1}{2}\Big(|0\rangle\langle 0| + |1\rangle\langle 1| + |0\rangle\langle 1| \frac{1}{2^L} \prod_{\ell}^{L} \text{tr}(R_{\hat{n}_0^{(\ell)}} R^{\dagger}_{\hat{n}_1^{(\ell)}}) + \text{h.c.}\Big), \end{aligned} \tag{31}$$

and

$$\begin{aligned} P_e(|X\rangle) &= \text{tr}(|X\rangle\langle X|\, \rho_e) \\ &= \frac{1}{2}\Big(1 + \frac{1}{2^L}\text{Re}\big(\prod_{\ell}^{L} \text{tr}(R_{\hat{n}_0^{(\ell)}} R^{\dagger}_{\hat{n}_1^{(\ell)}})\big)\Big). \end{aligned} \tag{32}$$

Again, applying the final $\pi/2$-pulse makes equation (32) equivalent to measuring $P_e(|0\rangle)$, which is what is done experimentally. Note, the rotation angles implicitly depend both on unit pulse time and repeats, and the axes depends on unit pulse time, as explained in Sec. III A. In addition to the nearest nuclear qubits, this experiment also senses the larger weak spin bath, which shows up as a much broader resonance indicating entanglement with the environment (Fig. 5). As discussed in the main text, the set of first order resonances ($k = 1$) are not resolvable due to overlap with the bath. At higher orders, as low as the second order ($k = 2$) as shown in Fig. 5, stronger coupled nuclear qubits separate from the bath and become individually resolvable. In addition to the three nuclear qubits considered in the main text, one can also start to see the next strongest nuclear qubit on the right side (positive parallel hyperfine coupling) of the bath, which becomes resolved at the third ($k = 3$) order. Note, the simulation shown is not a fit to the data, but a prediction based on previously measured hyperfine couplings (Table I). The couplings' initial measurement did involve the fitting of a similar simulation to this experiment, but this was not repeated here.

Nuclear qubit	$A_{\|\|}$ [kHz]	$A_{\perp}$ [kHz]
q_1	-118.8	68.4
q_2	-86.1	58.3
q_3	-46.4	67.7
q_4	10.1	25.4

TABLE I. **Four strongest nuclear qubits** No resonances other than the ones observed in Fig. 5 have been observed for this NV up to third order, indicating these are the four strongest coupled nuclear qubits. The similar couplings, particularly the sign of the parallel components, of the first three qubits are of central importance for their ability to be entangled in parallel (more information in Sec. V C). All simulations included these four nuclear qubits, even when an experiment did not intend to control some of them.

V. NUCLEAR GATES

A. Calibration

Each nuclear gate is calibrated by first setting the unit pulse time t to select the rotation axis and whether the rotation is conditional or unconditional, and second, setting the unit pulse repeats N to achieve a desired rotation angle. Based on the choice of coordinates for the Hamiltonian in equation (3), the unit pulse time can be chosen to rotate nuclear qubits about X and Z axes. As discussed in Sec. III A, X rotations occur resonantly, or for particular unit pulse times, given by equation (26). The direction of these can be conditioned on the state of the electron (CX gate) or independent of it (unconditional X gate). At all other times outside of these resonances, the rotation is unconditionally about the Z axis. Each of these gates can be visualized (Fig. 6) by considering each component of the rotation axes using the following identities on the nuclear rotation operators in equation (24):

$$\mathrm{tr}(R_{\hat{n}}(\phi)) = 2\cos(\phi/2) \rightarrow \phi = 2\arccos\left(\frac{1}{2}\,\mathrm{tr}(R_{\hat{n}}(\phi))\right), \tag{33}$$

$$\mathrm{tr}(\sigma_k R_{\hat{n}}(\phi)) = -2i\sin(\phi/2)n_k \rightarrow n_k = \frac{\mathrm{tr}(\sigma_k R_{\hat{n}}(\phi))}{2i\sin(\phi/2)} \tag{34}$$

Except in the case of parallel entanglement, all CX and X unit pulse times were chosen to be at the second order resonance. This provides well-individualized control on each nuclear qubit, with the crosstalk on other spins being approximately a Z gate. The flexibility in the choice of unit pulse time for Z gates allowed for quick unit pulse times to be used, of which the exact values were set based on the angular calibration. See table II for the unit pulse times used for all the gates.

Once the unit pulse times set the nuclear rotation axis, the net rotation angle can be calibrated. The choice of unit pulse time also affects the angular resolution $\Delta\phi = \phi^{(\ell)}$ of each rotation operator. Since the unit pulse time is often set to a resonance time for the rotation axes, $\phi^{(\ell)}$ is also fixed, up to which order resonances are used. The unit pulse can then be repeated N times to achieve a rotation angle $N\phi^{(\ell)}$, as highlighted in equation (25). Thus, once the $N = 1$ rotation angle $\phi^{(\ell)}$ is calculated, either using equation (33) numerically or closed form expressions as in [4], the repeats needed $N^{(\ell)}_{\mathrm{cal}}$ for a desired rotation angle $\phi^{(\ell)}_{\mathrm{target}}$ on the ℓth nuclear qubit can be calibrated according to:

$$N^{(\ell)}_{\mathrm{cal}} = \mathrm{round}(\phi^{(\ell)}_{\mathrm{target}}/\phi^{(\ell)}). \tag{35}$$

The discrete nature of angle calibration does introduces a finite angular error for each gate, but often this is small and can be reduced by using multiple types of DD unit pulses (see end of Sec. III A) or strategic choice of the magnetic field strength (see Sec. VII for details). For Z gate calibration, the unit pulse time was specifically chosen so that $N = 4$ (single XY8 unit pulse with minimal error) repeats created a $\pi/2$ rotation for each nuclear qubit. Almost all nuclear gates used in this work were rotations by $\pi/2$. The number of repetitions needed to achieve this net angle, as well as the absolute difference from $\pi/2$, is listed in table II for each nuclear qubit.

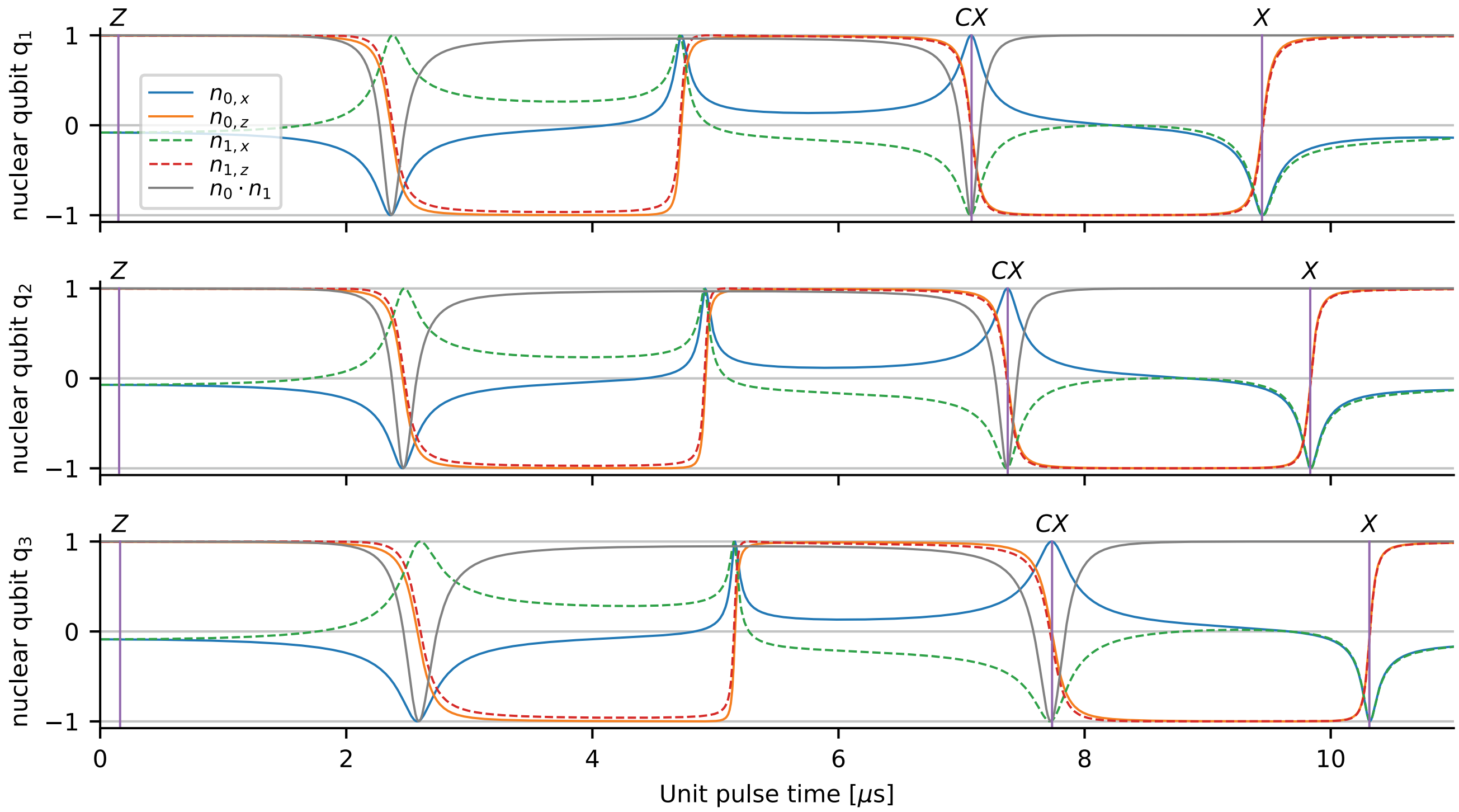

FIG. 6. **Nuclear qubit rotation axis components** Individual components of each nuclear qubit rotation axis $\hat{n}_j^{(\ell)}$ for each electron spin state $|j\rangle$ based on equation (34). Y components are 0 based on Hamiltonian frame. Unit pulse times used for each gate (table II) are marked with purple lines.

Gate	Nuclear qubit	Unit pulse time [μs]	Repeats	Gate time [μs]	|Angular error| [rad]
$CX_{\pi/2}$	q_1	7.081	7	49.6	0.042
	q_2	7.375	7	51.6	0.001
	q_3	7.736	5	38.7	0.009
$X_{\pi/2}$	q_1	9.442	7	66.1	0.000
	q_2	9.834	9	88.5	0.081
	q_3	10.314	12	123.8	0.040
$Z_{\pi/2}$	q_1	0.148	4	0.6	0.001
	q_2	0.154	4	0.7	0.000
	q_3	0.162	4	0.6	0.003

TABLE II. **Simulated nuclear gate calibration** DD unit pulse parameters used for each gate for each nuclear qubit. Simulation results verified experimentally in Sec. VIII B.

B. Parallel Z gates

The unit pulse times for Z gates were also chosen to lie in a region where they could be accurately parallelized in multi-nuclear qubit experiments. Prior to the first order resonances, each nuclear qubit's rotation axes are nearly identical and aligned along Z. Furthermore, a single unit pulse time can be chosen (averaged based on individual times in table II) to achieve approximately equal rotation angles on each nuclear qubit, resulting in a multi-qubit gate

of the form:

$$Z_L(\phi) = I_e \otimes Z_\phi^{(1)} \otimes ... \otimes Z_\phi^{(L)} \tag{36}$$

on L nuclear qubits. These gates are useful in general, but will be particularly useful in Sec. IX for the multiple quantum coherences method. Calculating the process fidelity F_p of the DD sequences against equation (36) with and without electron pulse error (table V B) shows high (≈99%) fidelity for each subset of qubits.

Nuclear qubit subsets	Unit pulse time [μs]	Unit pulse repeats	F_p without pulse error	F_p with pulse error
q_1, q_2	0.151	4	0.994	0.993
q_1, q_3	0.155	4	0.990	0.990
q_2, q_3	0.158	4	0.993	0.993
q_1, q_2, q_3	0.155	4	0.988	0.987

TABLE III. **Parallelized Z gate parameters and process fidelities** Other parallelized angles were used in this work, but $\pi/2$ is shown here as a representative example.

C. Parallel entanglement

In order to calibrate a parallel entangling gate, multiple steps must be taken to ensure high fidelity operation. Foremost, a maximal parallel entangling gate is possible when there exists a unit pulse time such that $(\hat{n}_0 \cdot \hat{n}_1)_\ell < 0$ for each targeted ℓth nuclear qubit [4]. Considering this quantity at first and second order (Fig. 7) for the four strongest nuclear qubits foremost shows there is an intersection (grey shaded region of Fig. 7a) between the three strongest nuclear qubits (q_1, q_2 and q_3), and subsets of them, at first order ($k = 1$) (Fig. 7a). At second order ($k = 2$), and therefore all subsequent orders, there does not exist any intersection for these nuclear qubits. Fig. 7 additionally shows that the fourth strongest nuclear qubit q_4 does not satisfy this requirement with any other nuclear qubits at either order. q_4 is only included in Fig. 7 and this analysis to highlight its spectator role in these experiments and to quantify any non-maximal entanglement generated with it. Thus, for these specific hyperfine couplings, at most three nuclear qubits could be entangled with a single gate with the correct choice of N.

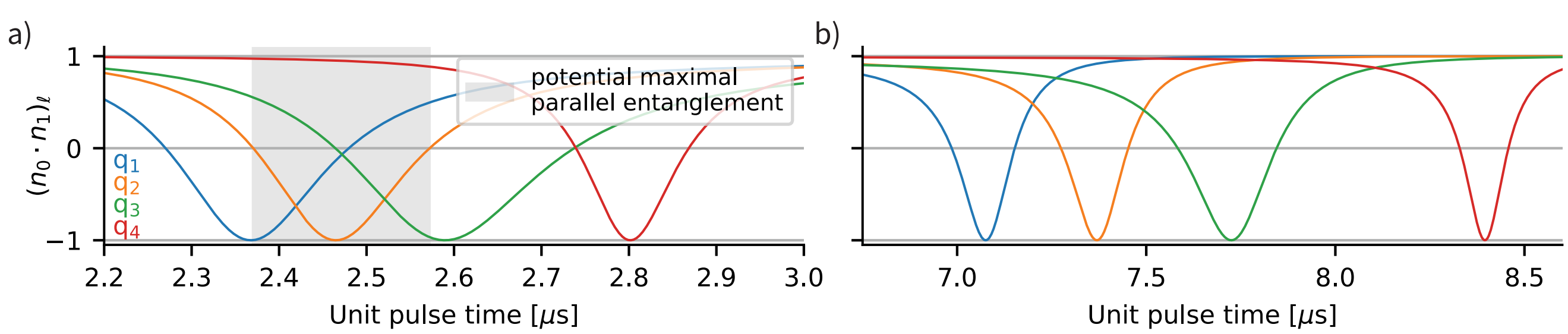

FIG. 7. **Conditional rotation axis alignment (a)** First order, referred to in main text as "parallel order", resonances. The grey shaded regions is set based on the intersection of at least two of the $(\hat{n}_0 \cdot \hat{n}_1)_\ell$ curves that are negative and thus indicates when one DD gate can entangle multiple nuclear qubits. **(b)** Second order, or "individual order", resonances. Only maximal bipartite entangling gates are possible at this, and all subsequent, order for these four nuclear qubits.

Once it has been determined that a parallel entangling gate is possible, the precise calibration of the DD parameters can be determined using the algorithm of Takou et. al. [9]. The key details of how this algorithm was used in this work are summarized here. For each subset of nuclear spins containing at least two nuclear spins, *i.e.* $\{\{q_1, q_2\}, \{q_1, q_3\}, \{q_2, q_3\}, \{q_1, q_2, q_3\}\}$ in this work, scan the parameter space of (t, N) pairs and calculate the M-way non-unitary entangling power $\varepsilon_{p,M}(\mathcal{E})$. Then, save which (t, N) pairs generate a $\varepsilon_{p,M}(\mathcal{E})$ greater than a set threshold. M refers to the total number of qubits in the system, including the electron and L_{total} will refer to the number of nuclear qubits, $M = L_{\text{total}} + 1$. L_{total} is in principle fixed for a given NV-nuclear register based on the number of weakly coupled nuclear qubits resolvable from the spin bath, in this work $L_{\text{total}} = 4$. The number of targeted nuclear qubits is given by L, which partitions L_{total} according to $L_{\text{total}} = L + L_{\text{not targeted}}$. L changes based on the number of nuclear qubits targeted for entanglement in a given experiment. With these counting definitions, the non-unitary

entanglement metric is (approximately) given by

$$\varepsilon_{p,M}(\mathcal{E}) \approx \frac{\varepsilon_{p,M}(U_{DD})}{2}\left(1+\prod_{\substack{\ell\in\text{not}\\ \text{targeted}}}^{L_{\text{total}}-L} G_1^{(\ell)}\right). \tag{37}$$

where $\varepsilon_{p,M}(U_{DD})$ is the unitary entangling power calculated over the targeted nuclear qubits in the subset, given by

$$\varepsilon_{p,M}(U_{DD}) = \left(\frac{d}{d+1}\right)^M \prod_{\substack{\ell\in\text{targeted}\\ \text{subset}}}^{L} (1-G_1^{(\ell)}), \tag{38}$$

and $G_1^{(\ell)}$ is the first Makhlin invariant given by

$$G_1^{(\ell)} = \left(\cos^2\frac{N\phi^{(\ell)}}{2} + (\hat{n}_0\cdot\hat{n}_1)^{(\ell)}\sin^2\frac{N\phi^{(\ell)}}{2}\right)^2. \tag{39}$$

This approximation is very accurate; for a comparison between the more complicated exact form of $\varepsilon_{p,M}(\mathcal{E})$ with equation 37, see [9]. $G_1^{(\ell)}$ ranges from 0 (maximal entanglement between the electron and nuclear qubit q_ℓ) and 1 (no entanglement). Ignoring the constant factor of $(d/(d+1))^M$ in equation 38 (convention adopted throughout this work), leads $\varepsilon_{p,M}(U_{DD})$ to take values between 0 (no entanglement between electron and targeted nuclear qubits) to 1 (maximal entanglement between electron and targeted nuclear qubits). If any residual entanglement is generated with non-targeted nuclear qubits, their Makhlin invariant will be less than 1, thus lowering the non-unitary entangling power $\varepsilon_{p,M}(\mathcal{E})$. The non-unitary entangling power is bounded from above by the unitary entangling power: $\varepsilon_{p,M}(\mathcal{E}) \leq \varepsilon_{p,M}(U_{DD})$, with equality occurring when no residual entanglement is generated [9]. Therefore, the use of the non-unitary entangling power over the unitary version is crucial when considering subsets of targeted nuclear qubits so that residual entanglement with non-targeted nuclear qubits is properly taken into account.

In applying this algorithm to identify optimal parallel entangling gates, the range of t values can be set based on the intersection of $\hat{n}_0\cdot\hat{n}_1$ ranges described above (shaded region of Fig. 7a). If no result is found for some subset of nuclear qubits, one can consider a larger range of N values until a valid case is returned, or lower the entangling power threshold value. The former is would be preferred to generate higher fidelity gates, while the later may be preferred to generate faster gates. Applying this algorithm with a threshold of $\varepsilon_{p,M}(\mathcal{E}) > 0.8$ to the specific hyperfine couplings used in this work leads to the cases presented in Fig. 8 and summarized in Table V C. Importantly, this procedure shows that it is possible to entangle all three nuclear qubits at once, as well as entangle all three subsets of nuclear qubits, without inducing considerable residual entanglement.

Target nuclear qubits	Non-targeted nuclear qubits	Unit pulse time [μs]	Unit pulse repeats	Gate time [μs]	Target $\epsilon_{p,M}(U_{DD})$	Non-unitary $\epsilon_{p,M}(\mathcal{E})$
q_1, q_2, q_3	q_4	2.472	6	14.8	0.9940	0.9635
q_1, q_2	q_3, q_4	2.363	29	68.4	0.9630	0.8411
q_1, q_3	q_2, q_4	2.474	24	59.4	0.9997	0.9842
q_2, q_3	q_1, q_4	2.532	13	32.9	0.9884	0.8872

TABLE IV. **Optimal parallel entanglement gate parameters from Fig. 8**

.

In addition to these optimized cases, we also consider the effects of parallel entangling gates that generate considerable residual entanglement with non-targeted nuclear qubits. For these gates, parameters are chosen more arbitrarily and will serve as examples showing the experimental effects of residual entanglement. Targeting entanglement with the subset of nuclear qubits q_1, q_2 and q_2, q_3, the unit pulse times were chosen at the intersection of each respective $(\hat{n}_0\cdot\hat{n}_1)_\ell$ curve and N was set to 6. This value of N leads to considerable residual entanglement with the non-targeted nuclear qubits, as quantified by significantly reduced non-unitary entangling power (Table V C). Note these faulty gates, and the experiments using them, are included only to highlight how residual entanglement can manifest in measurable signals (see Sec. IX C for additional details).

VI. GENERALITY AND EXTENSIONS

This section contextualizes the results of this work by simulating and averaging over many nuclear-qubit configurations to determine the probability that a given configuration can be entangled in parallel, along with the associated

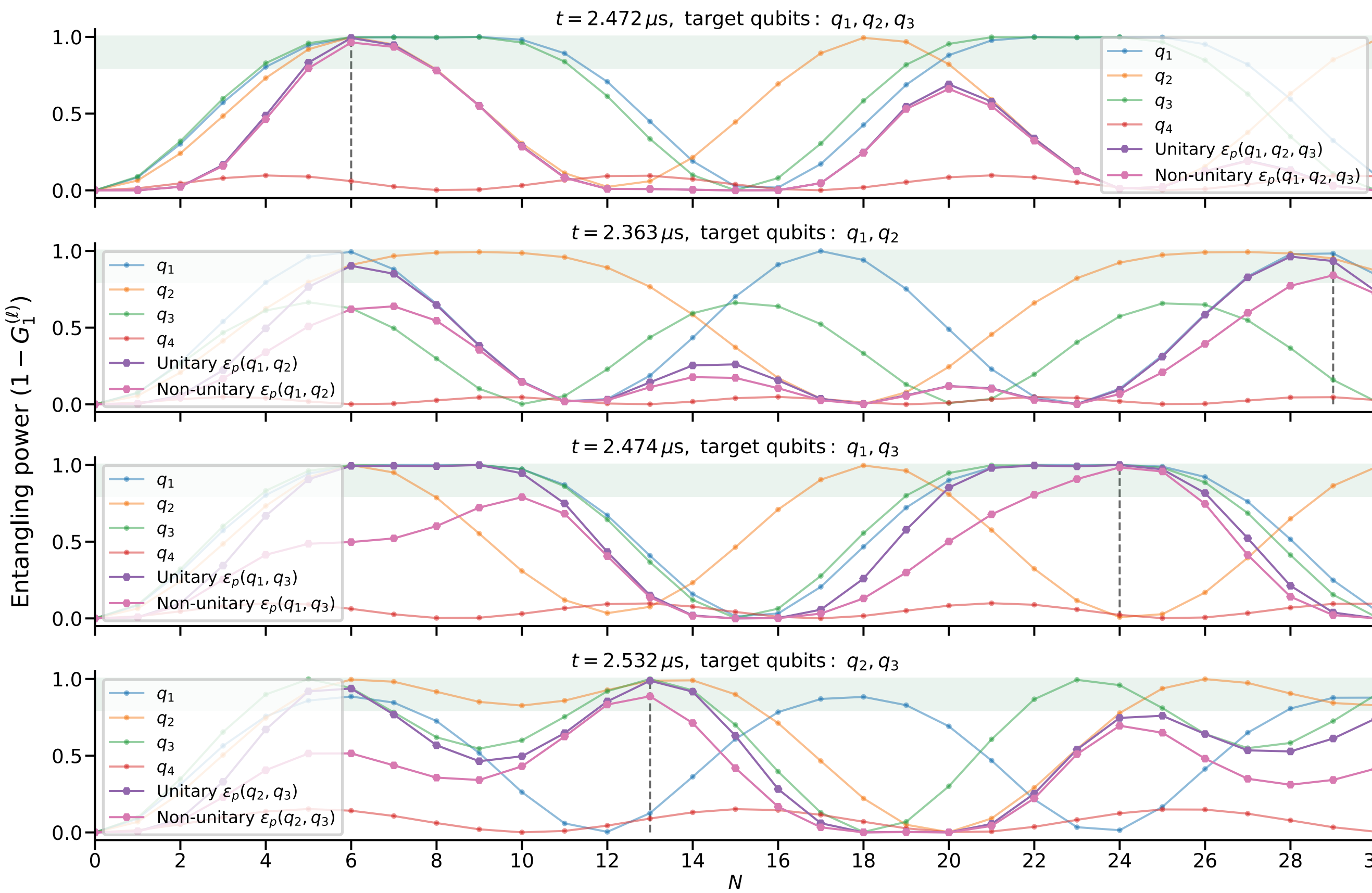

FIG. 8. **Optimal parallel entanglement cases** Green shaded region denotes the threshold of $\varepsilon_{p,M}(\mathcal{E}) > 0.8$ set for the gate parameter search. Grey dashed lines denote optimal N determined. At these values, the entangling power of the non-targeted nuclear qubits is near zero.

Target nuclear qubits	Non-targeted nuclear qubits	Unit pulse repeats	Gate time [μs]	Target $\epsilon_{p,M}(U_{DD})$	Non-unitary $\epsilon_{p,M}(\mathcal{E})$
q_1, q_2	q_3, q_4	2.418	6	0.9789	0.5429
q_1, q_3	q_2, q_4	2.520	6	0.8878	0.4690

TABLE V. **Parallel entanglement gate parameters that generate residual entanglement** Note how it is possible that the targeted entangling power to be large (near 1) but the non-unitary entangling power to be much smaller due to residual entanglement. This effect is due to the next strongest nuclear qubit that was not targeted (q_3 in the case of targeting q_1 and q_2), while q_4 only slightly lowered the metric.

gate times and fidelities. To do so, we randomly generate other spatial distributions of ^{13}C nuclear qubits around a NV center. To simplify the model, nuclear qubits are placed at continuous locations uniformly randomly sampled within fixed sphere of radius R_c. The radius of the cut-off sphere is fixed based on the atomic density of diamond, the ^{13}C abundance and the total number of nuclear qubits to be considered. For example, at the natural abundance of 1.1%, the radius of the cut-off sphere needed to encapsulate $N_{\text{nuc}} = 100$ nuclear qubits is $R_c \approx 2.3$nm. Then, N_{nuc} random locations with distances less than R_c are uniformly sampled within the sphere. For each configuration of nuclear qubits, the hyperfine coupling to the electron is calculated based on the physical location relative to the NV center at the origin. Since our focus is on weakly coupled nuclear qubits—those farther from the NV center—the influence of discrete lattice site distances on the hyperfine coupling is negligible.

Far fewer than the total number of nuclear qubits N_{nuc} included in the initial model are considered part of the NV-nuclear register. Only nuclear qubits with hyperfine parameters between a certain, physically motivated, range are considered. First, each configuration is pre-filtered so that no strongly coupled nuclear qubits are present. Only configurations where all hyperfine components (parallel and perpendicular) are less than 200 kHz are considered further. This cutoff is based on the electron spin transition inhomogeneous linewidth of $\sqrt{2}/\pi T_2^* \approx 200$kHz. This pre-filter process passes about 1 in every 3 configurations ($\approx$1400 configurations were examined to generate 500 passable

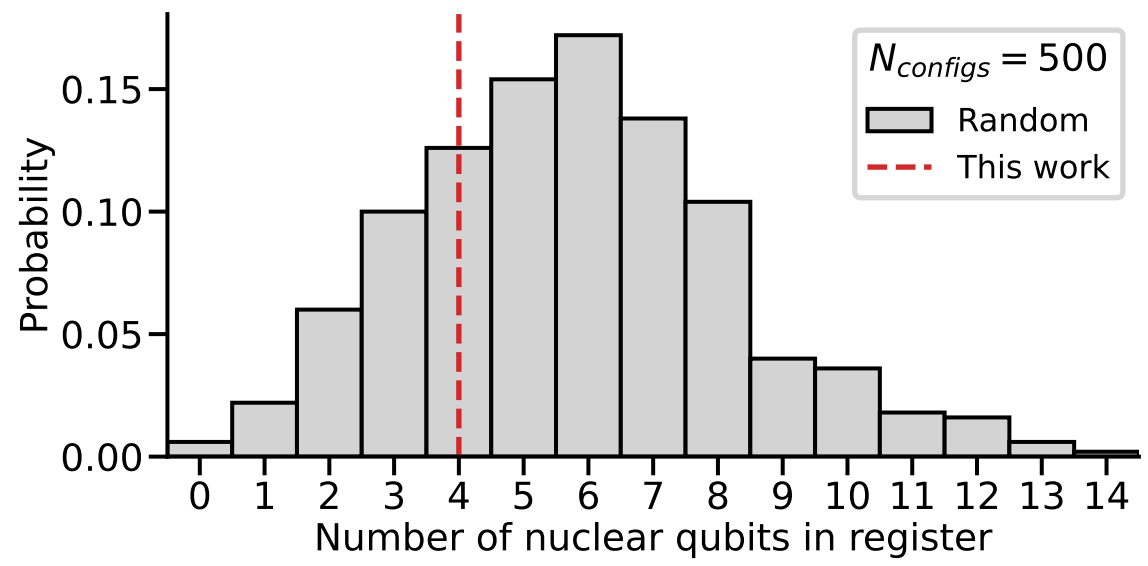

FIG. 9. **Sampled distribution of number of weakly coupled nuclear qubits in register** Each configuration sampled here already passed the pre-filter of containing no strongly coupled nuclear qubits. The number of register nuclear qubits L_{total} is therefore based on the number of weakly coupled nuclear qubits stronger than $|A_{\|}| > 15$kHz and $A_{\perp} > 10$kHz, ensuring they could be resolved from the spin bath.

configurations, hence ≈36% pass). Configurations that include one or more strongly coupled nuclear qubits are more difficult to entangle in parallel since DD is primarily intended to control weakly coupled nuclear qubits. After passing this pre-filter, the configuration is included in the averaging analysis that follows. To ensure register nuclear qubits are not part of the spin bath of very weakly and similarly coupled spins, only parallel hyperfine couplings greater than 15 kHz are considered for parallel entanglement. While the parallel component largely determines separation from the spin bath, a lower bound on the perpendicular component was also placed at 10 kHz to ensure the nuclear qubits included can be rotated sufficient fast (reasonable N). Together, this ensures that only the nuclear qubits that could reasonably have their hyperfine couplings measured with DD spectroscopy are included. In other words, nuclear qubits that would contribute to the spin bath are not considered because they could not be experimentally measured using DD spectroscopy. Note, techniques such as DD-radio frequency can be employed to also utilize nuclear qubits with effectively no perpendicular hyperfine coupling, extending the register size further [10].

At natural abundance, these bounds lead to on average $\bar{L}_{\text{total}} \approx 6$ nuclear qubits (standard deviation of $\sigma_{L_{\text{total}}} \approx 2.5$) in the register and therefore considered for parallel entanglement. This was averaged over 500 pre-filtered configurations and is the sample size for all simulations that follow, unless stated otherwise. The exact distribution of register sizes sampled is shown in Fig. 9. Foremost, this indicates that the nuclear qubit configuration studied in this work is on the smaller end of the expected value, minus the spread. For reference, the nuclear register studied in [7] did not contain any nuclear qubits with hyperfine coupling (parallel or perpendicular) greater than even 80kHz and was able to resolve 6 nuclear qubits from the spin bath. The weakest nuclear qubits resolved had components of $|A_{\|}|, A_{\perp} \approx 15$kHz; comparable to the weakest nuclear qubit (q_4) observed for the configuration studied in this work.

Of the configurations that did not contain any strongly coupled nuclear qubits, the register qubits are then run through the parallel entanglement calibration algorithm of Takou *et al.* [9] described in the previous section V C. Note that the parameters of the algorithm (Table VI) used were the same as those used to calibrate the parallel gates of this work. Due to the presence of spin bath experimentally, additional care needs to be taken when sweeping potential unit pulse times. Unit pulse times the lie within the spin bath region will lead to significant residual entanglement with bath spins that are not being included in this model. To account for this, unit pulse times corresponding to the bath region were removed from the calibration algorithm. This avoided region was set based on the resonance times of the lower bound hyperfine coupling used.

For a given subset of nuclear qubits that can be entangled in parallel, there can be multiple DD gate parameters that satisfy the threshold conditions set. However, only one parallel gate for each subset should be saved, which can be chosen a number of ways. Here, we consider picking the parallel entangling gate with the shortest duration and also the gate with the largest non-unitary entangling power, and compare both outcomes. The trade off in choosing the fastest gate may be a loss of fidelity (although the entangling power threshold dictates how much can be lost), while higher fidelity gates may take longer to execute. When considering the parallel entanglement of larger numbers of nuclear qubits, there is often only one possible parallel gate and thus no trade off to be made.

Averaging over the weakly coupled registers reveals gate duration distributions for each type of parallel entangling gate, as well as sequences of two-qubit gates at any order k (Fig. 10a). From this, the speedup of the parallel gate over each the sequential gates can be calculated (Fig. 10b). The only slightly irregular feature of our results is the speed up factor for the $L = 3$ gate, which is on the high end or slightly outside of 1.5× the interquartile range. This is due to the fact that our $L = 3$ parallel gate was able to be performed with high fidelity on the first oscillation of each nuclear qubit (small N value). This placed the absolute duration on the lower end of 1.5× the interquartile range, while the sequential gate durations were near the median. Consequently, the ratio of durations is large, but

Parameter	Value	Primary effect
^{13}C concentration	1.1%	Register size L_{total}
Max hyperfine	$\|A_\parallel\|, A_\perp < 200$kHz	Fraction of usable configurations
Min hyperfine	$\|A_\parallel\| > 15$kHz, $A_\perp > 10$kHz	Register size L_{total}
Unit pulse time t resolution	1ns	Probability of parallel entanglement
Max N	50	Probability of parallel entanglement
Min non-unitary entangling power	0.8	Probability of parallel entanglement

TABLE VI. **Simulation settings for parallel entanglement of random nuclear qubit configurations** In addition to the primary effect listed, there can be other secondary effects. For example, while the ^{13}C concentration primarily effects how large the nuclear qubit register is, the register size can have an effect on the probability of parallel entanglement being possible. Overall, these are conservative parameters — increasing the maximum N or lowering the entangling threshold has a large effect on the probability of a given parallel entangled state to be formed. Assuming a unit pulse times in the range of $\approx$2 to 4μs, an N as large as 500 to 250 may be considered before the entangling operation takes 1ms (roughly the electron T_2 time). Given the computational complexity of needing to average over many configurations here, such large parameter were not explored.

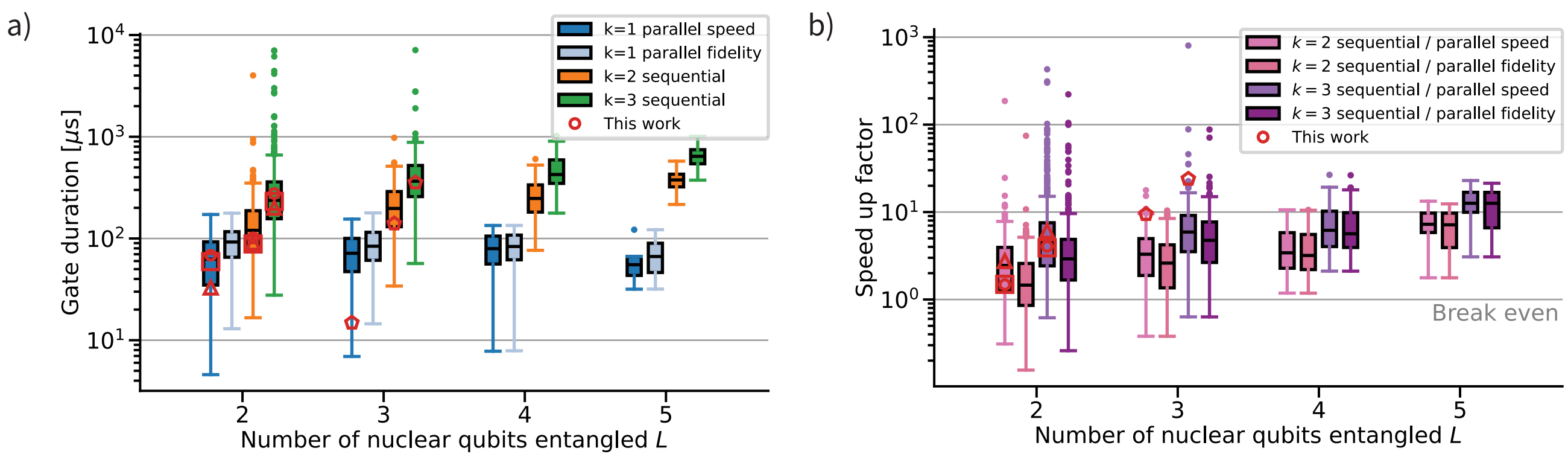

FIG. 10. **Expected parallel entanglement durations (a)** Box plots of the distributions of durations for each entangling gate. "parallel speed" refers to parallel gates calibrated to minimize duration and "parallel fidelity" refers to parallel gates calibrated to maximize non-unitary entangling power. The middle black line represents the median, the box spans the interquartile range (IQR), the whiskers extend to 1.5× the IQR, and points indicate outliers. Red markers of different shapes denote the specific cases observed in this work. **(b)** Parallel entangling gate speedup calculated as the ratio of durations in each specific register.

other cases sampled were still much larger.

While there are many factors that can impact the fidelity of a gate, we analyze one of the predominate effects here — residual entanglement generated with nuclear qubits in mixed states. An expression for the gate fidelity due to residual entanglement was derived in Takou *et al.* [4] and the key details are summarized here. The entangling gate fidelity due to residual entanglement F_r is given by

$$F_r = \frac{1}{2^{L+1}+1}\left(1 + 2^{L-1}\sum_{k}^{2^{L_{\text{total}}-L}}\left|\sum_{j=0,1} c_j^{(k)} p_j^{(k)}\right|^2\right) \tag{40}$$

where the summation over k runs over all possible bit strings of length $L_{\text{total}} - L$ (the number of nuclear qubits in the environment). For each bit string k, let M of the bits be 0 and $P = L_{\text{total}} - L - M$ of the bits be 1. Then, the terms $c_j^{(k)}$ and $p_j^{(k)}$ are calculated based on tracing out the environmental nuclear qubits. Given the known DD rotation operators acting on the environment, these have explicit expressions given by:

$$\begin{aligned} c_j^{(k)} &= \prod_{m\in\{\text{bits}=0\}}^{m_M} \langle 0|R_{\hat{n}_j^{(m)}}(\phi^{(m)})|0\rangle \\ &= \prod_{m\in\{\text{bits}=0\}}^{m_M} \left[\cos\left(\phi^{(m)}/2\right) - i n_{z,j}^{(m)} \sin\left(\phi^{(m)}/2\right)\right] \end{aligned} \tag{41}$$

$$p_j^{(k)} = \prod_{s \in \{\text{bits}=1\}}^{s_P} \langle 1 | R_{\hat{n}_j^{(s)}}(\phi^{(s)}) | 0 \rangle$$
$$= \prod_{s \in \{\text{bits}=1\}}^{s_P} \left[-i \left(n_{x,j}^{(s)} + n_{y,j}^{(s)} \right) \sin\left(\phi^{(s)}/2 \right) \right] \tag{42}$$

where $R_{\hat{n}_j^{(m)}}(\phi^{(m)})$ are the DD rotation operators given in equation 24. Note, one can assume $n_{y,j}^{(s)} = 0$ for the frame of the Hamiltonian used here. In the ideal case of no residual entanglement generated, $c_j^{(k)}$ and $p_j^{(k)}$ are 1 and thus $F_r = 1$.

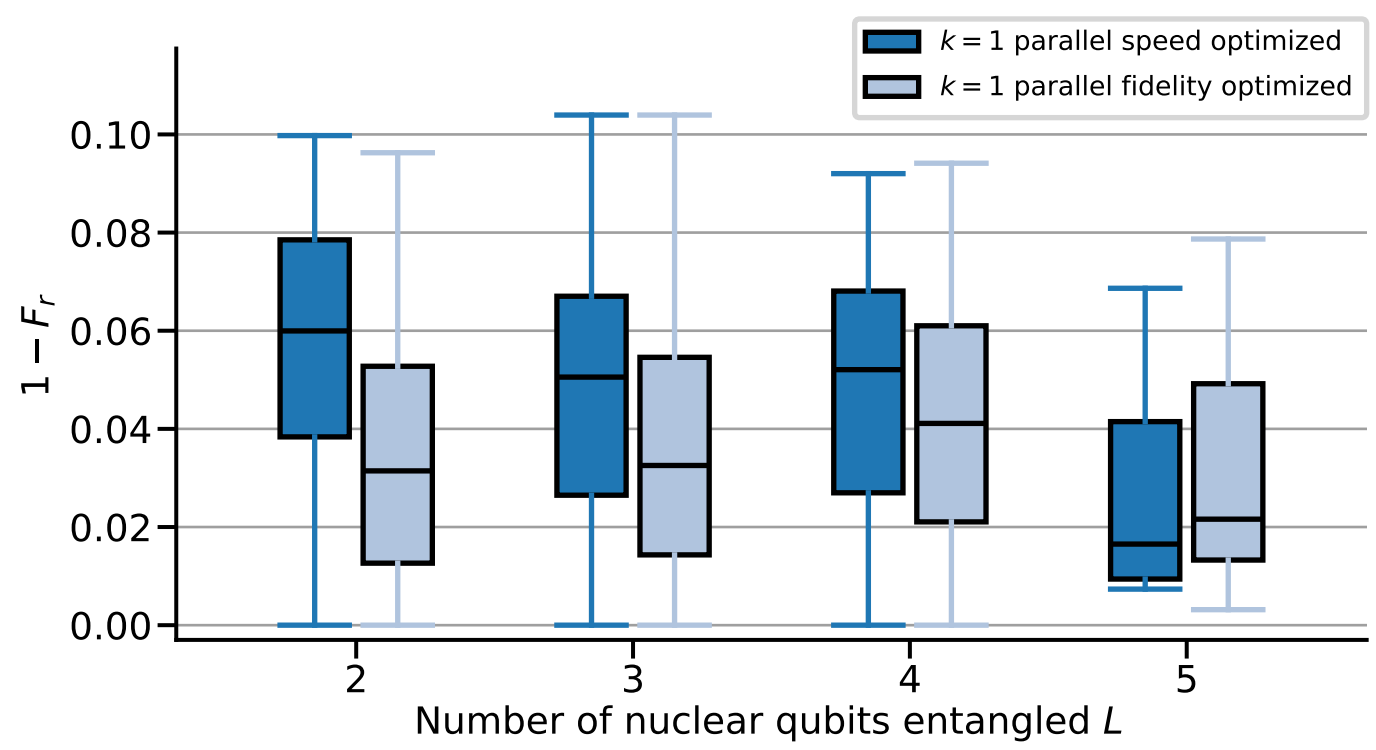

FIG. 11. **Expected parallel entangling gate error rates due to residual entanglement** Error rates $(1 - F_r)$ simulated here are directly related to the non-unitary entangling power threshold set in the calibration process. Stricter (larger) thresholds will lead to lower error rates, but fewer potential parallel entanglement cases. The middle black line represents the median, the box spans the interquartile range (IQR), the whiskers extend to 1.5× the IQR, and points indicate outliers.

Applying equation 40 to the configurations that hosted parallel entanglement highlights low error rates $(1 - F_r)$, all less than ≈ 0.1 (Fig. 11). The excellent performance of these gates is based on the considerably large (near 1) non-unitary entangling power threshold set during the calibration process. As expected, the gates that were chosen for their shorter duration have a slightly higher error rate than the gates chosen for the larger non-unitary entangling power. For additional details on the relationship between the entangling power and gate fidelity, see [4] Note, the cases where the fidelity exactly equals 1 indicates that all of the nuclear qubits in the register were used in the parallel entanglement process (no environment qubits). Experimentally, there are other sources of error in the entangling gate. The leading factor for the loss of fidelity observed in this work likely stems from pulse errors in controlling the electron. This accentuates the dephasing of the electron due to the spin bath, which also lowers fidelity. Additional analysis of these effects could be incorporated, following the results of [9].

One can also consider diamond samples that have different concentrations of ^{13}C nuclei using this framework. On one side, with higher concentrations, there are more weakly coupled nuclear qubits in the register to participate in the parallel entanglement (Fig. 12a), but on the other side, this may lead to increased residual entanglement and in turn lower probabilities of such a parallel gate existing. Similarly, with lower concentrations, the register size decreases but so does the potential residual entanglement. Owing to the computational complexity associated with including more nuclear qubits in a register, smaller sample sizes (200 configurations) and unit pulse time steps (10ns) were used when considering different concentrations. A primary bottleneck in running the parallel entanglement algorithm with a large number configurations, each with a large number of nuclei in the system, is the evaluation of every possible subset of nuclear qubits to target entanglement with. This power set of N_{qubits} nuclear qubits has an exponentially large size of $2^{N_{\text{qubits}}}$ so for very large system sizes nearing ≈ 20 nuclear qubits, there are ≈ 1 million subsets to check over all gate parameters. In this case, pre-filtering for nuclear qubits that have an intersection of $(\hat{n}_0 \cdot \hat{n}_1)_{(\ell)} < 0$ before iterating though all gate parameters would be essential for large scale calculations. At lower concentrations, there tend to be lower likelihoods to form larger parallel entangled states (Fig. 12b), which can be attributed to the smaller register sizes. Similarly, as concentration increases, there tends to be a loss of likelihood. However, in these cases, fewer configurations pass the pre-filtering process for containing a strongly coupled nuclear qubit. If one conditions the likelihood on the register being only weakly coupled (Fig. 12c), then at increased concentrations there is a similar,

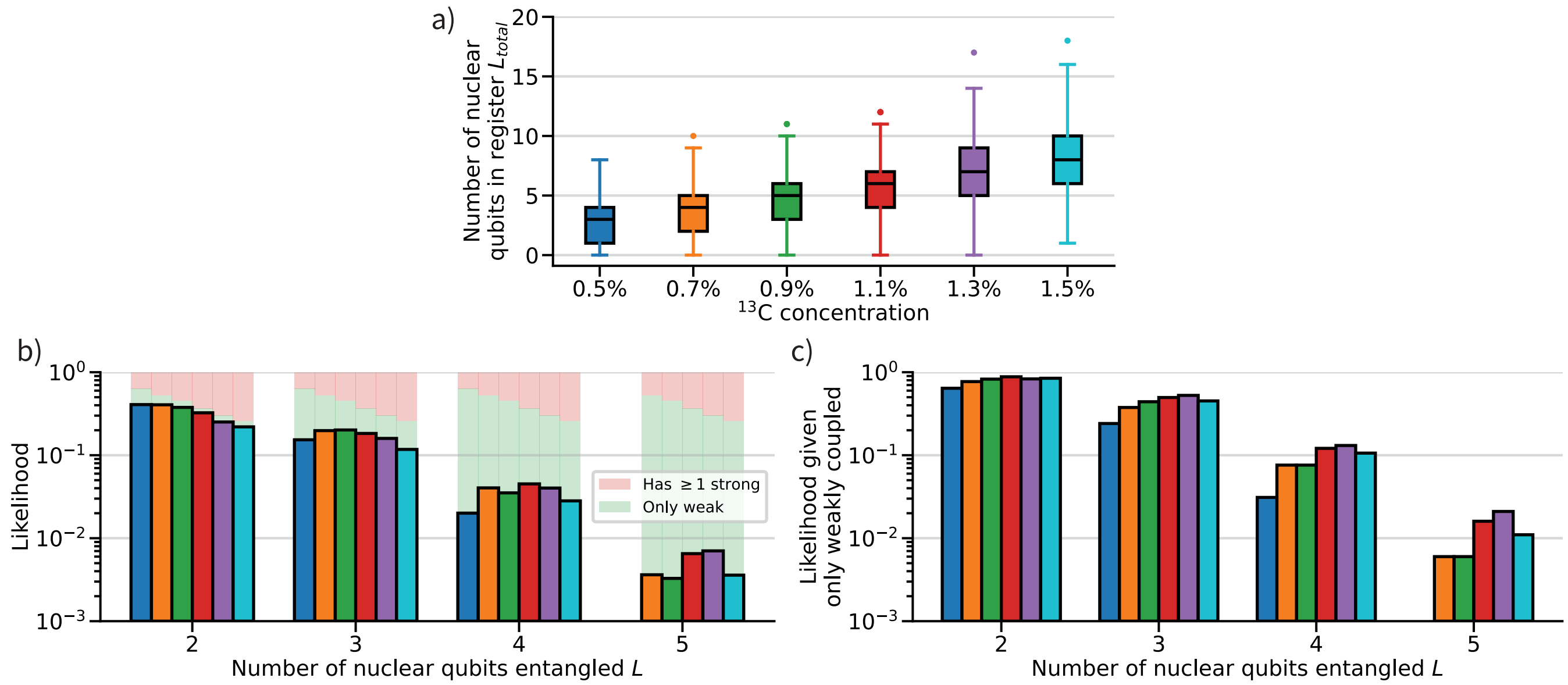

FIG. 12. **Nuclear qubit concentration effects on parallel entanglement (a)** Total number of weakly coupled nuclear qubits in register. **(b)** Probability of any configuration hosting parallel entanglement of different sizes L. The red shaded regions denote the portion of configurations that have at least one strongly coupled nuclear qubit and are not considered for parallel entanglement. The green shaded regions denote the rest of the configurations that are considered for parallel entanglement. As concentration increases, the probability of having at least one strongly coupled nuclear qubit increases. Bar colors correspond to the concentrations in (a). **(c)** Probability of a weakly coupled configuration hosting parallel entanglement.

if not slightly larger likelihood to form larger entangled states in parallel. Note, a finer-grained parameter space of DD gates may further accentuate these changes. Future research taking into these computational complexities can be dedicated to finding an optimal concentration, where optimality can be defined according to likelihood of parallel entanglement, size of the entangled state, duration or fidelity of the gate.

VII. MAGNETIC FIELD OPTIMIZATION

A. Alignment

The external magnetic field was mounted on a set of two micrometer translation stages in the plane normal to the NV axis. One controlled the height from the optical table and the other controlled right-left translation. Translation along the NV symmetry axis to and from the sample was adjusted using a threaded casing around the magnet attached to an optical kinematic mount. The kinematic mount has two angular micrometers to adjust the polar and azimuthal angles of the field.

The alignment and field strength of the magnet in a given position were measured using the resonant frequencies of pulsed electron spin resonance experiments for both $|m_s = 0\rangle \rightarrow |m_s = \pm 1\rangle$ transitions [11]. The translation micrometers were adjusted to optimize field strength, then the angular micrometers were adjusted to optimize field alignment. The process converged after 2-3 rounds of optimization. The final misalignment angle was measured to be $0.4^{\circ}{}^{+1.6}_{-0.4}$, and the field strength was 338.19 ± 0.14G. The alignment and field strength were periodically checked to correct for drift over extended periods of time.

B. Field strength

The particular magnetic field strength of 338G was chosen to minimize the nuclear gate times and errors. Based on the bandwidth of the microwave electronics, and the fact that both electron spin transitions are needed to align the field, the magnetic field strength was limited from above at approximately 700G. A sufficiently large field is also convenient for estimating DD resonance times made under a large field approximation [4, 7].

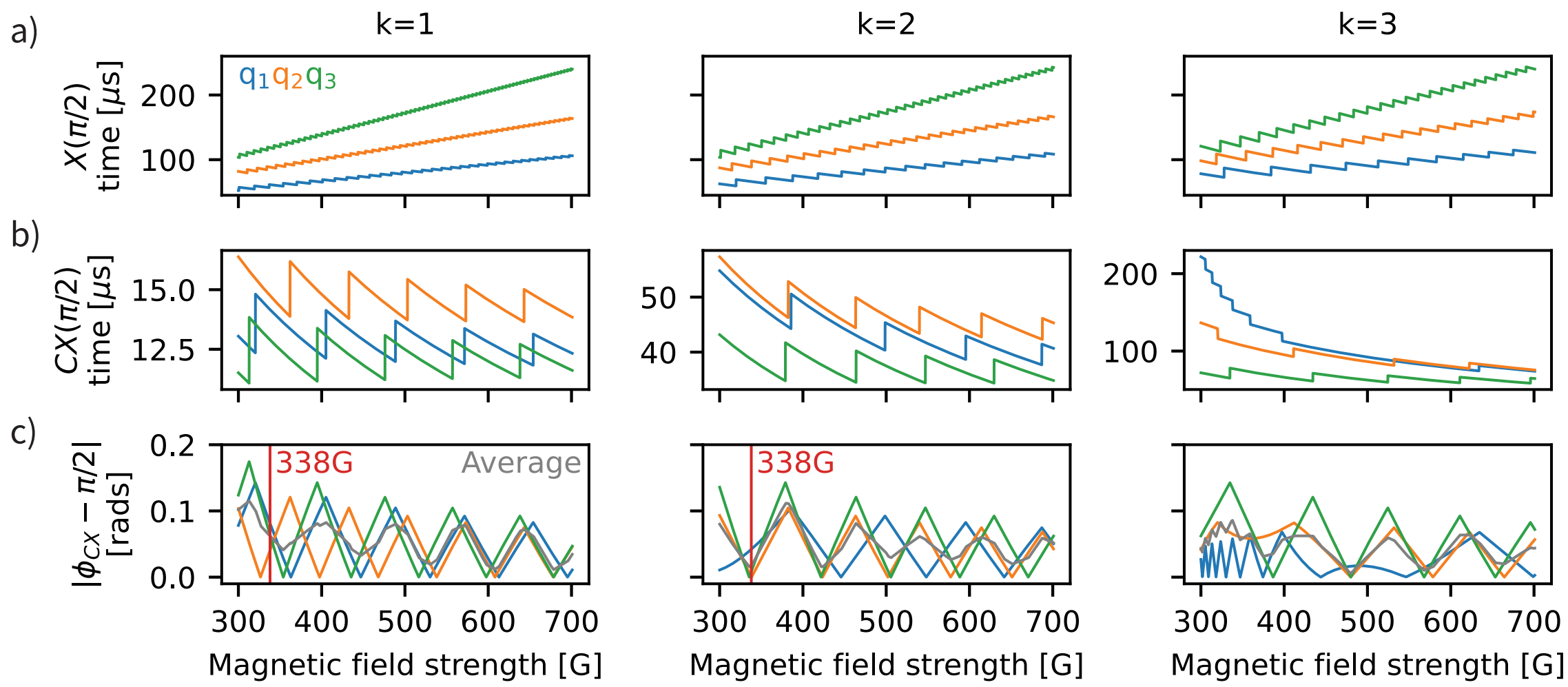

FIG. 13. **Magnetic field effect on nuclear gate times (a)** Total gate time for $X_{\pi/2}$ rotation of each nuclear qubit at first three resonance orders. Although the first and second order gates have nearly identical times, the second order gates use fewer pulse repeats, which means few operations on the electron, so these were used. **(b)** Conditional version of (a). Second order was again used, but this was to save time compared to third order. **(c)** Absolute conditional angular error from $\pi/2$ to select specific field strength.

The magnetic field strength's effect on each nuclear qubit's total gate time was first considered (Fig. 13a,b). While the conditional gate times decrease slightly at larger fields, the unconditional gates increase linearly with field strength. This is due to the relative strengths of each qubit's Larmor frequency set by the field and their fixed hyperfine couplings. At larger fields (higher Larmor frequencies) the free evolution axes for each electron spin state $\hat{p}_j^{(\ell)}$ (Sec. III) become more aligned, thus requiring more DD unit pulse repeats to achieve a particular rotation angle. Therefore, from an efficiency perspective, a smaller field should be used to decrease nuclear gate time.

Next, a particular field strength can be chosen to minimize the angular errors from the discrete nature of DD angular control. Since unconditional X gates typically needed more repetitions than conditional gates, their angular resolution was sufficiently small which already led to small gate errors. On the other hand, at lower field strengths, the conditional gates do not need many repetitions, which could lead to considerable angular error in achieving a desired rotation angle. Since all two-qubit conditional gates used $\pi/2$ rotations, the absolute angular error from this value was considered at the first three resonance orders (Fig. 13c). Each nuclear qubit's conditional gate time increased considerably between orders, which led us to not use any third order gates in this work. Based on the average error for the three strongest nuclear qubits, two field strengths were found that minimized both the first and second order conditional gate angular errors: 338G and 436G, of which the smaller was chosen to minimize unconditional gate time.

VIII. INDIVIDUAL NUCLEAR QUBIT CONTROL CIRCUITS

A. Nuclear qubit initialization and tomography

To initialize nuclear qubits into partially polarized states, polarization from the electron was transferred to each nuclear qubit following the circuit [6] shown in Fig. 14a. In [6], initialization was verified via changes in the electron's T_2^*, but this method is not feasible here due to the shorter $T_2^* \approx 2\mu$s of this sample. Instead, each nuclear qubit's initialization was verified with state tomography experiments (Fig. 14b) as detailed in Sec. VIII B. However, this approach does not yield a direct measurement of the nuclear qubit initialization fidelity because there are also errors in the state tomography process that can not be separated. It is also useful to note that the 2μs green laser pulse used to reinitialize the electron has very little effect on nuclear qubit polarization and coherence times once they've been initialized. The supplement of [6] contains detailed results of nuclear qubit lifetimes under green illumination and such experiments were not repeated here.

Multiple nuclear qubits were initialized using the same sub-circuit sequentially with the electron reinitialized between each iteration. This allowed for selective initialization of the quantum register. Alternative initialization schemes, such as PulsePol [12], could have been used at room temperature, but simulation showed comparable fidelities and

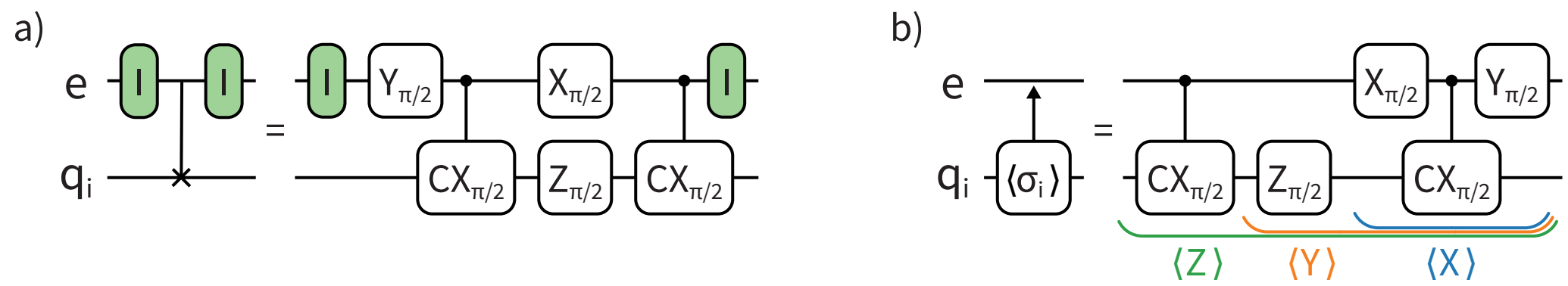

FIG. 14. **Nuclear initialization and tomography circuits (a)** Swap based initialization circuit of individual nuclear qubits. **(b)** State tomography circuit of individual nuclear qubits. Brackets below each subset of gates are color-coded to which nuclear expectation value they swap onto the electron for measurement.

durations. Furthermore, such protocols require many more electron reinitialization steps than the sequential swap approach. Without real-time electron charge state checks [13], this introduces additional experimental noise and is already one of the leading causes of discrepancy between simulation and experimental contrast present in this work.

To readout complete information from each individual nuclear qubit, state tomography can be performed (Fig. 14b). This process maps nuclear qubit expectation values onto the electronic state, which is then measured. While individual nuclear qubit tomography is sufficiently high fidelity, multi-nuclear state tomography used to measure inter-nuclear correlations is much worse due to crosstalk between nuclear gates. Simulations showed a low ($\approx 60\%$) average readout fidelity in the two nuclear qubit case, with the three nuclear qubit case behaving even worse.

B. Experimental nuclear gate calibration

Using nuclear qubit state tomography, a variety of simple circuits (Fig. 15) were developed to verify nuclear initialization and control of each nuclear qubit. In each circuit, the number of unit pulse repeats for a particular gate was varied and partial state tomography along the Y axis was performed. Y axis tomography was chosen because most gates rotate about the X axis, so an orthogonal direction will detect coherent rotations of nuclear qubits, and Y tomography is shorter than Z tomography. Additionally, the state of the electron was varied to observe the (un)conditionality of each gate. To highlight this effect, the electron was either kept in the initialized $|0\rangle$ state, or flipped to $|1\rangle$ prior to gate calibration and back again before tomography began. This highlights that all gates calibrated are indeed (un)conditional as expected. All experiments were run using simulated gate parameters from table II and all of the fitting results for the repetitions needed for a $\pi/2$ (table VII) nearly match the simulation results, with the exception of the CX gate of nuclear qubit q_1. Even so, in that case the average between the results for the two electron states are in agreement for which N creates a $\pi/2$ rotation.

Nuclear qubit	Electron State	Sim CX $N_{\pi/2}$	Exp CX $N_{\pi/2}$	Sim X $N_{\pi/2}$	Exp X $N_{\pi/2}$	Sim Z $N_{\pi/2}$	Exp Z $N_{\pi/2}$
q_1	0	6.7	7.6 ± 0.1	6.6	6.8 ± 0.1	4.0	4.0 ± 0.0
	1	7.6	5.6 ± 0.1	6.5	7.2 ± 0.1	4.0	4.1 ± 0.0
q_2	0	6.8	7.0 ± 0.1	9.2	9.2 ± 0.2	4.0	4.0 ± 0.0
	1	7.0	6.9 ± 0.1	9.2	9.6 ± 0.2	4.0	4.0 ± 0.0
q_3	0	4.8	5.0 ± 0.1	11.1	11.2 ± 0.1	4.0	4.0 ± 0.0
	1	5.3	4.8 ± 0.1	11.1	10.8 ± 0.2	4.0	4.0 ± 0.0

TABLE VII. **Experimental nuclear gate calibration fit results** All values shown have been rounded to one decimal place and when rounded to the nearest integer match the calculated values in simulation table II.

IX. MULTIPLE QUANTUM COHERENCES

A. Idealized theory

Here we derive the multiple quantum coherences (MQC) signal measured for the DD gate implementation of the method. The main idealization made in the following analysis is the absence of nuclear gate crosstalk when considering more than one nuclear qubit. This idealization means that when targeting the rotation of one of the nuclear qubits, others are transformed with the identity operator. As discussed extensively in the main text, this is not the case for DD, but in the single nuclear qubit case this analysis will be exact up to other errors. Even in the multinuclear

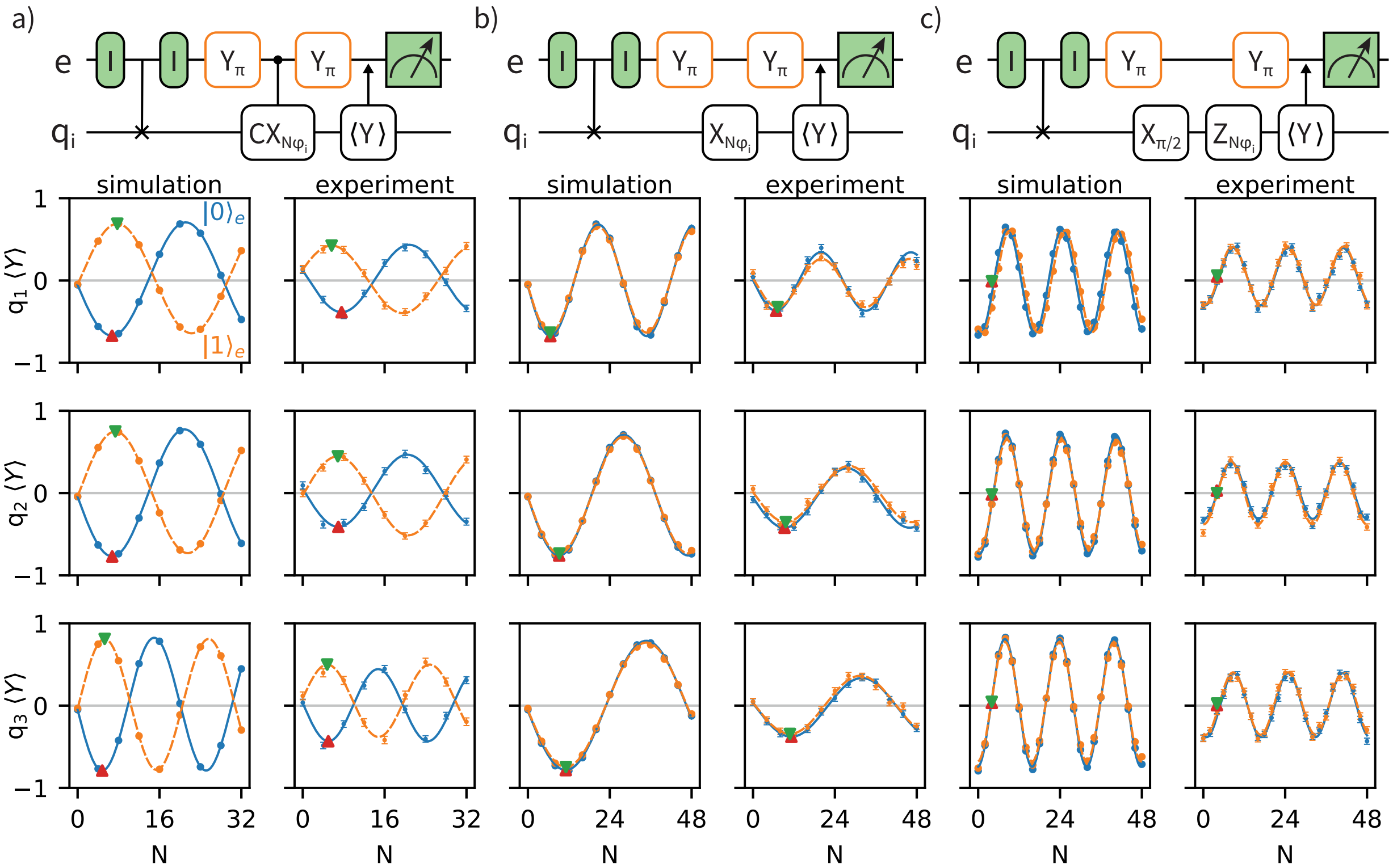

FIG. 15. **Experimental nuclear gate calibration (a,b,c)** CX,X,Z nuclear gate calibration by sweeping each unit pulse repeat (nuclear rotation angle), respectively. Both data (points with error bars) and simulation (points) were fit (solid and dashed curves) to determine which number of repeats corresponds to a $\pi/2$ rotation (results in table VII). Both electron states ($|0\rangle_e$ blue and $|1\rangle_e$ orange) were used prior to sweeping each nuclear gate to highlight conditionality as in (a), or unconditionality in (b) and (c). Red (green) triangles denote $N(\pi/2)$ for the electron state $|0\rangle_e$ ($|1\rangle_e$). Data points in experimental columns denote mean values taken over 10^6 shots and error bars denote photon shot noise.

case, the final result of this analysis holds given that we can correct for the crosstalk by optimizing GHZ state fidelity (more details in Sec. IX B). In light of this, we consider only a single idealized form of the gate that creates multipartite entanglement, temporarily neglecting the complicated geometry of the parallel entanglement gates. This simplification, and its implication will later be addressed in this section.

Consider the electron and L initialized nuclear qubits in the state $|\psi_0\rangle = |0\rangle^{\otimes L+1}$. To prepare for entanglement, rotate the electron with $Y_{\pi/2}$ and each nuclear qubit with $X_{\pi/2}$. This yields:

$$\begin{aligned} |\psi_1\rangle &= |X\rangle \otimes |{-Y}\rangle^{\otimes L} \\ &= \frac{1}{\sqrt{2}}\big(\, |0\rangle \otimes |{-Y}\rangle^{\otimes L} + |1\rangle \otimes |{-Y}\rangle^{\otimes L}\,\big). \end{aligned} \tag{43}$$

Now apply an $L+1$-qubit entangling gate of the form:

$$C_e X_{\pi/2}^{\otimes L} = |0\rangle\langle 0| \otimes X_{\pi/2}^{\otimes L} + |1\rangle\langle 1| \otimes X_{-\pi/2}^{\otimes L}. \tag{44}$$

Sequences of two-qubit gates takes this form in the absence of crosstalk and the parallel gate is locally equivalent to this. Transforming $|\psi_1\rangle$ gives the entangled state of:

$$\begin{aligned} C_e X_{\pi/2}^{\otimes L} |\psi_1\rangle &= \frac{1}{\sqrt{2}}\big(\, |0\rangle \otimes \big(X_{\pi/2} |{-Y}\rangle\,\big)^{\otimes L} + |1\rangle \otimes \big(X_{-\pi/2} |{-Y}\rangle\,\big)^{\otimes L}\big) \\ &= \frac{1}{\sqrt{2}}\big(\, |0\rangle \otimes (-i)^L |1\rangle^{\otimes L} + |1\rangle \otimes |0\rangle^{\otimes L}\,\big) \\ &= \frac{1}{\sqrt{2}}\big(\, |0\rangle \otimes |1\rangle^{\otimes L} + \frac{1}{(-i)^L} |1\rangle \otimes |0\rangle^{\otimes L}\,\big) = |\mathrm{GHZ}\rangle\,, \end{aligned} \tag{45}$$

where in the last line the global phase was adjusted. Let $1/(-i)^L = e^{i\alpha_L}$ be the relative phase of the two states in the entangled state due to the local operations. Now apply an equal phase gate Z_ϕ to each nuclear qubit:

$$\begin{aligned} |\mathrm{GHZ}_\phi\rangle &= \frac{1}{\sqrt{2}}(e^{iL\phi/2}|0\rangle \otimes |1\rangle^{\otimes L} + e^{i\alpha_L}e^{-iL\phi/2}|1\rangle \otimes |0\rangle^{\otimes L}) \\ &= \frac{1}{\sqrt{2}}(|0\rangle \otimes |1\rangle^{\otimes L} + e^{i(\alpha_L - L\phi)}|1\rangle \otimes |0\rangle^{\otimes L}). \end{aligned} \tag{46}$$

Now disentangle the system using the same entangling gate as before:

$$\begin{aligned} |\mathrm{Dis.}\rangle &= \frac{1}{\sqrt{2}}(|0\rangle \otimes (X_{\pi/2}|1\rangle)^{\otimes L} + e^{i(\alpha_L - L\phi)}|1\rangle \otimes (X_{-\pi/2}|0\rangle)^{\otimes L}) \\ &= \frac{1}{\sqrt{2}}(|0\rangle \otimes (-i|Y\rangle)^{\otimes L} + e^{i(\alpha_L - L\phi)}|1\rangle \otimes |Y\rangle^{\otimes L}) \\ &= \frac{1}{\sqrt{2}}(|0\rangle \otimes |Y\rangle^{\otimes L} + e^{i(2\alpha_L - L\phi)}|1\rangle \otimes |Y\rangle^{\otimes L}) \\ &= \frac{1}{\sqrt{2}}(|0\rangle + e^{i(2\alpha_L - L\phi)}|1\rangle) \otimes |Y\rangle^{\otimes L}, \end{aligned} \tag{47}$$

where, as desired, the relative phase of the electron now carries information about the size of the entangled state. One could derive the expression for the electron probability to be in $|0\rangle$ by rotating it back with $Y^{-1}_{\pi/2}$ (which is done experimentally), or assuming a measurement in the $|X\rangle$ basis. Performing the latter here for simplicity, tracing out the nuclear qubits we have:

$$|\psi_e\rangle = \frac{1}{\sqrt{2}}(|0\rangle + e^{i(2\alpha_L - L\phi)}|1\rangle), \tag{48}$$

and therefore:

$$\begin{aligned} P_e(|X\rangle) &= |\langle X|\psi_e\rangle|^2 \\ &= \frac{1}{2}(1 + \cos(L\phi - \delta_L)), \end{aligned} \tag{49}$$

where $\delta_L = 2\alpha_L$ is the overall phase shift defined in the main text. Now let us verify a phase shift of π in the $L = 1$ case. Using $1/(-i)^L = e^{i\alpha_L}$, we see that for $L = 1$ the LHS is i, which has a phase $\alpha_1 = \pi/2$. Thus $\delta_1 = 2\alpha_1 = \pi$ as desired. For larger L, this phase shift could be calculated as well, but because the optimization was able to adjust local operations before entanglement, the exact phase shift may change, and appears to in many of the optimized simulations and experiments. This is a harmless effect that maintains the frequency amplification of each nuclear state's phase onto the electron in the end.

Now let us address the particular form of the entangling gate used in equation (44). For multi-qubit parallel entangling gates, each nuclear qubit rotation operator has a unique geometry, which can be generally written as:

$$U_{\mathrm{para}} = |0\rangle\langle 0| \bigotimes_{\ell}^{L} R_{\hat{n}_0^{(\ell)}}(\phi^{(\ell)}) + |1\rangle\langle 1| \bigotimes_{\ell}^{L} R_{\hat{n}_1^{(\ell)}}(\phi^{(\ell)}). \tag{50}$$

In order to form a multipartite maximally entangled (MME) state, this operator must act on the proper initial separable state. Although finding such states could be analyzed analytically here, this is handled by the optimization of Sec. IX B due to nuclear gate crosstalk making local rotations complicated. For now, consider the action of this gate on an initial state $|X\rangle \otimes |0\rangle^{\otimes L}$, which gives an entangled state of the form:

$$|\mathrm{MME}\rangle = \frac{1}{\sqrt{2}}\left(|0\rangle \bigotimes_{\ell}^{L} \left|R^{(\ell)}_{n_0}\right\rangle + |1\rangle \bigotimes_{\ell}^{L} \left|R^{(\ell)}_{n_1}\right\rangle\right), \tag{51}$$

where $\left|R^{(\ell)}_{n_j}\right\rangle = R_{\hat{n}_j^{(\ell)}}(\phi^{(\ell)})|0\rangle$. In order for this to be a MME state, $\left|R^{(\ell)}_{n_0}\right\rangle$ and $\left|R^{(\ell)}_{n_1}\right\rangle$ need to be anti-parallel, or $\left\langle R^{(\ell)}_{n_1}\middle|R^{(\ell)}_{n_0}\right\rangle = 0$, for each nuclear qubit q_ℓ. This creates a state that can be transformed into a GHZ state with local rotations (*i.e.*, locally equivalent). Equivalently, the optimization process sets these local rotations before

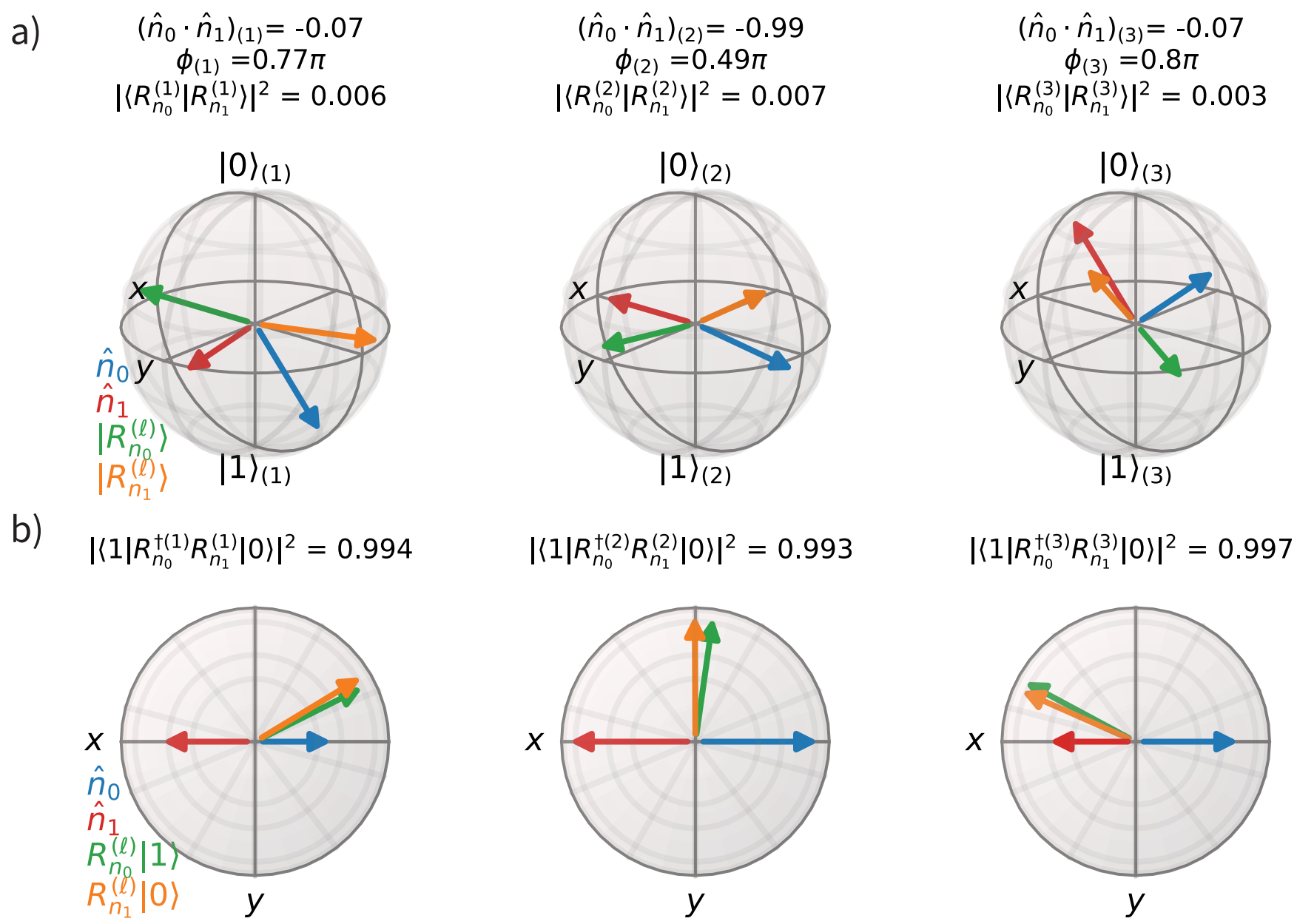

FIG. 16. **Parallel entangling gate geometry (a)** Entangling transformation from an ideal nuclear qubit $|0\rangle$ initial state. Since the $L = 3$ parallel gate unit pulse time was chosen approximately on resonance for nuclear qubit q_2, we expect $(\hat{n}_0 \cdot \hat{n})_{(2)} \approx -1$ and an angle of $\phi_{(2)} \approx \pi/2$. For the other off resonance nuclear qubits, since $\hat{n}_0 \cdot \hat{n}_1 \neq -1$, a large rotation angle is expected to create anti-parallel states after rotation. To quantify this behavior, one can consider the overlap $|\langle R^{(\ell)}_{n0}|R^{(\ell)}_{n_1}\rangle|^2$ of the two quantum states, which should be near zero to generate a MME state from a geometric perspective. **(b)** Disentangling transformations of the same gate, but applied to a GHZ state of equation (45). Here the transformed state overlap should be near one to properly disentangle the MME state.

entanglement so that a GHZ state is formed from the application of the parallel gate. As an example, Fig 16 considers the $L = 3$ parallel entangling gate used in this work. The top panel, Fig. 16a, shows each of the states described in equation 51 for each nuclear qubit, highlighting their orthogonality and thus the MME nature of the state created by the parallel gate.

Furthermore, the disentangling ability of the parallel gate needs to be carefully considered. The relevant transformations to consider now are $R_{\hat{n}_0^{(\ell)}}(\phi^{(\ell)})\,|1\rangle$ and $R_{\hat{n}_1^{(\ell)}}(\phi^{(\ell)})\,|0\rangle$ for each nuclear qubit, based on equation (46). As desired, each of these transformations leads to approximately identical states on each nuclear qubit's Bloch sphere, thus disentangling all of the nuclear qubits from the electron (Fig. 16b). Therefore, we can expect the conclusion (equation (49)) of the MQC circuit analysis to hold given that the optimization handles the creation of a GHZ state, which then allows the parallelized entangling gate to properly disentangle the system.

B. Circuit optimization

As discussed in the main text and the previous section, in multinuclear implementations of the MQC method, the local operations before entanglement were optimized to maximize GHZ state fidelity using simulations. This optimization was performed using SciPy's dual annealing algorithm with equation (45) defining the ideal target state. The exact state fidelity was computed using the QuTiP software package according to:

$$F_s = \left(\mathrm{tr}\sqrt{\sqrt{\rho_1}\rho_2\sqrt{\rho_1}} \right)^2 \tag{52}$$

for two density matrices $\rho_{1,2}$ being compared. During optimization, each nuclear qubit's rotation axis was set to X or Z by fixing the unit pulse times, and the total rotational angles were treated as free parameters set by the number of unit pulse repeats, as depicted in Fig. 3 of the main text. Using a targeted X and Z gate for each nuclear qubit allowed each qubit to be rotated anywhere in its Bloch sphere, with the annealing bounds set to rotations of 2π for each gate. Dual annealing was chosen due to the highly non-convex loss landscape with many local optima, in addition to the fact that nuclear rotation angles can only change in discrete steps. The optimization results that were

used in each simulation and experiment are listed in Tables IX B, IX B, and IX B. The unit pulse times used to set the rotation axis of each gate are the same as in Table II.

Even in cases where a subset of nuclear qubits were not targeted for entanglement, their effects were still included in the simulated dynamics (including the fourth strongest nuclear qubit observed from Sec. IV). This allows any residual entanglement that was generated to manifest itself in the MQC signals. Overall, the only deviation from the expected MQC behavior appeared when using parallel entangling gates that intentionally generated residual entanglement with nuclear qubits in mixed states (Fig. 19). These gates produced a beating pattern that is explained in the following section Sec. IX C. When using the optimal parallel entangling gates that minimize residual entanglement (Fig. 18 and 17), comparisons against the unoptimized rotations show drastic improvements in the MQC frequency mapped onto the electron as well as improvements in contrast, ultimately making this method experimentally viable.

	Parallel		Sequential	
q_ℓ	N_x	N_z	N_x	N_z
q_1	10	6	8	6
q_2	6	8	6	10
q_3	14	1	32	4

TABLE VIII. **Four-qubit entanglement optimization results**

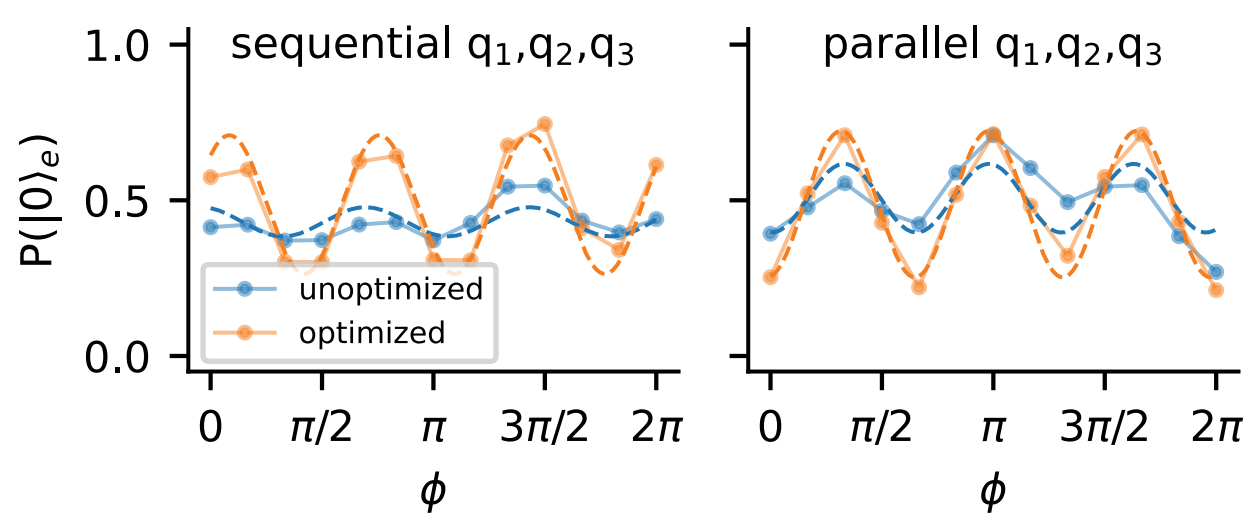

FIG. 17. **Four-qubit entanglement optimization MQC comparison** Dashed lines are fits to simulations for visualization purposes. A resolution of $\pi/6$ was used for the parallel phase gate here, as in experiments.

Parallel									Sequential								
q_ℓ	N_x	N_z	q_ℓ	N_x	N_z	q_ℓ	N_x	N_z	q_ℓ	N_x	N_z	q_ℓ	N_x	N_z	q_ℓ	N_x	N_z
q_1	22	4	q_1	6	0	q_2	9	4	q_1	8	7	q_1	8	11	q_2	9	7
q_2	8	3	q_3	12	2	q_3	9	3	q_2	9	3	q_3	14	3	q_3	12	6

TABLE IX. **Three-qubit entanglement optimization results**

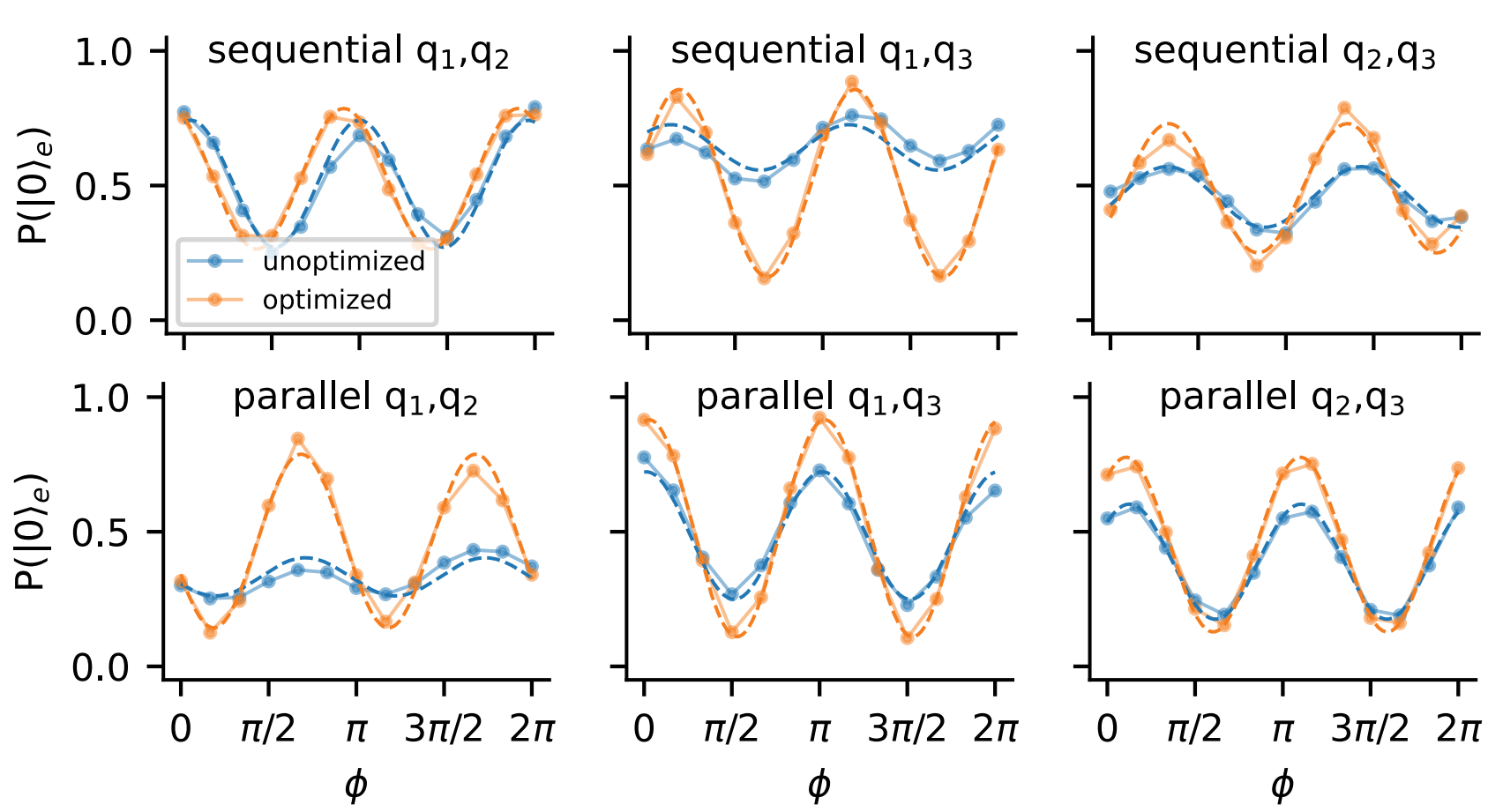

FIG. 18. **Three-qubit entanglement optimization MQC comparison**

Parallel

q_ℓ	N_x	N_z	q_ℓ	N_x	N_z
q_1	8	0	q_2	9	6
q_2	9	5	q_3	12	1

TABLE X. **Three-qubit entanglement, with residual entanglement, optimization results**

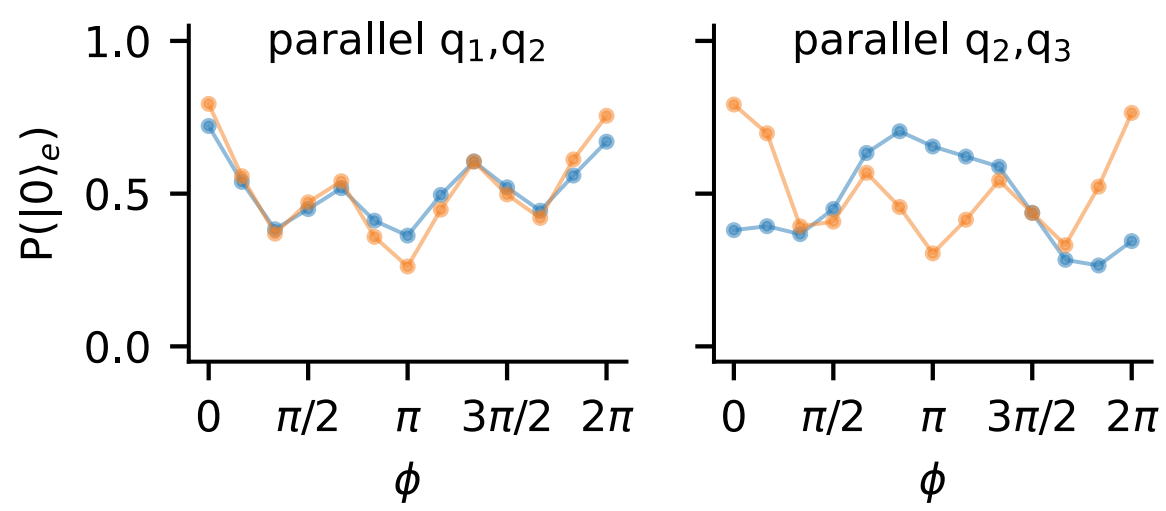

FIG. 19. **Three-qubit entanglement, with residual entanglement, optimization MQC comparison** See Sec. IX C for an explanation of the oscillations.

C. Residual entanglement effects on MQC signal

In this section we prove that entanglement generated with mixed states effects the expected frequency measured in the MQC signal, as displayed in the $L = 2$ simulations (Fig. 19). We begin this analysis similar to the idealized MQC case, but now include an additional mixed state nuclear qubit in the system:

$$\rho_0 = |0\rangle\langle 0|^{\otimes L+1} \otimes \frac{1}{2}\mathbb{1}. \tag{53}$$

Assume no gate crosstalk between the initialized spins for simplicity, but track the crosstalk effects on the mixed state. The net preparation gate U_p on the register is therefore given by:

$$U_p = Y_{\pi/2} \otimes X_{\pi/2}^{\otimes L} \otimes U_m, \tag{54}$$

where U_m is unconditional crosstalk on the mixed state. Therefore:

$$\begin{aligned}\rho_1 &= U_p \rho_0 U_p^\dagger \\ &= |X\rangle\langle X| \otimes |{-Y}\rangle\langle {-Y}|^{\otimes L} \otimes \frac{1}{2} U_m U_m^\dagger \\ &= |X\rangle\langle X| \otimes |{-Y}\rangle\langle {-Y}|^{\otimes L} \otimes \frac{1}{2}\mathbb{1}.\end{aligned} \tag{55}$$

However, the crosstalk during the parallel entangling operation is considerably (although not maximally) conditional on the electron state. Let the conditional rotation operators for the crosstalk on the mixed state be $R_0(\theta)$ and $R_1(\theta)$ giving a full operator of:

$$U_E = |0\rangle\langle 0| \otimes X_{\pi/2}^{\otimes L} \otimes R_0(\theta) + |1\rangle\langle 1| \otimes X_{-\pi/2}^{\otimes L} \otimes R_1(\theta), \tag{56}$$

which is the same as equation (44), but with conditional crosstalk considered. Applying this, we have:

$$\begin{aligned}\rho_E &= U_E \rho_1 U_E^\dagger \\ &= \frac{1}{4}\Big(|0\rangle\langle 0| \otimes |1\rangle\langle 1|^{\otimes L} \otimes \mathbb{1} + |0\rangle\langle 1| \otimes e^{-i\alpha_L} |1\rangle\langle 0|^{\otimes L} \otimes R_0 R_1^\dagger \\ &+ |1\rangle\langle 0| \otimes e^{i\alpha_L} |0\rangle\langle 1|^{\otimes L} \otimes R_1 R_0^\dagger + |1\rangle\langle 1| \otimes |0\rangle\langle 0|^{\otimes L} \otimes \mathbb{1}\Big).\end{aligned} \tag{57}$$

As discussed in Sec. V B, the MQC phase gate applied to the entangled state is naturally parallelized, which includes effects on the mixed state. Now that the system is entangled with the mixed state, the unconditional crosstalk effects of the phase gate no longer vanish like in ρ_1. Therefore, with the form of the phase gate given by:

$$Z_L(\phi) = \mathbb{1}_e \otimes Z_\phi^{\otimes L+1}, \tag{58}$$

we have:

$$\begin{aligned}\rho_{E,\phi} &= Z_L(\phi)\rho_E Z_L^\dagger(\phi)\\ &= \frac{1}{4}\Big(|0\rangle\langle 0| \otimes |1\rangle\langle 1|^{\otimes L} \otimes \mathbb{1} + |0\rangle\langle 1| \otimes e^{i(L\phi-\alpha_L)} |1\rangle\langle 0|^{\otimes L} \otimes Z_\phi R_0 R_1^\dagger Z_\phi^\dagger \\ &+ |1\rangle\langle 0| \otimes e^{-i(L\phi-\alpha_L)} |0\rangle\langle 1|^{\otimes L} \otimes Z_\phi R_1 R_0^\dagger Z_\phi^\dagger + |1\rangle\langle 1| \otimes |0\rangle\langle 0|^{\otimes L} \otimes \mathbb{1}\Big).\end{aligned} \tag{59}$$

Now disentangle the system with the same entangling operator as before like usual:

$$\begin{aligned}\rho_{\rm dis} = \frac{1}{4}\Big(&|0\rangle\langle 0| \otimes |Y\rangle\langle Y|^{\otimes L} \otimes \mathbb{1} + |0\rangle\langle 1| \otimes e^{i(L\phi-2\alpha_L)} |Y\rangle\langle Y|^{\otimes L} \otimes R_0 Z_\phi R_0 R_1^\dagger Z_\phi^\dagger R_1^\dagger \\ &+ |1\rangle\langle 0| \otimes e^{-i(L\phi-2\alpha_L)} |Y\rangle\langle Y|^{\otimes L} \otimes R_1 Z_\phi R_1 R_0^\dagger Z_\phi^\dagger R_0^\dagger + |1\rangle\langle 1| \otimes |Y\rangle\langle Y|^{\otimes L} \otimes \mathbb{1}\Big).\end{aligned} \tag{60}$$

Let the ϕ dependent operator appearing in the mixed state component be $A(\phi,\theta) = R_0 Z_\phi R_0 R_1^\dagger Z_\phi^\dagger R_1^\dagger$. Tracing out all nuclear qubits for just the electron state then gives:

$$\begin{aligned}\rho_e &= {\rm tr}_\ell\, \rho_{\rm dis}\\ &= \frac{1}{2}\Big(|0\rangle\langle 0| + |1\rangle\langle 1| + |0\rangle\langle 1| e^{i(L\phi-2\alpha_L)}\, {\rm tr}[A(\phi,\theta)] + {\rm h.c.}\Big).\end{aligned} \tag{61}$$

Thus neglecting the last electronic rotation and assuming a measurement in the $|X\rangle$ basis, we have:

$$P_e(|X\rangle) = \frac{1}{2}\Big(1 + \frac{1}{2}{\rm Re}\big(e^{i(L\phi-2\alpha_L)}\, {\rm tr}[A(\phi,\theta)]\big)\Big), \tag{62}$$

which in the limit of no entangling crosstalk, $A = \mathbb{1}$, returns equation (49) as expected.

Now let us assume the a generic form of the conditional crosstalk rotation operators $R_j(\theta)$. Let the rotation axes be $\hat{n}_0 = (x_0, 0, z_0)$ and $\hat{n}_1 = (x_1, 0, z_1)$ and calculate ${\rm tr}[A(\phi,\theta)]$:

$$\begin{aligned}{\rm tr}[A(\phi,\theta)] = 2\cos^4(\theta/2) &+ 2\big((x_0x_1 + z_0z_1)^2 + (z_0x_1 - x_0z_1)^2\cos(\phi)\big)\sin^4(\theta/2)\\ &-\frac{1}{2}\big(z_0^2 - 2x_0x_1 + 4z_0z_1 + z_1^2 + (x_0 - x_1)^2\cos(\phi)\big)\sin^2(\theta)\\ &+2(x_0x_1 + z_0z_1)(x_0z_1 - z_0x_1)\sin^2(\theta/2)\sin(\theta)\sin(\phi).\end{aligned} \tag{63}$$

Combining this expression with $e^{iL\phi}$ in equation (62) will result in sinusoidal terms with frequencies of the sum and difference between $L\phi$ and ϕ; $(L \pm 1)\phi$. Thus, in the case of $L = 2$, we can expected two frequency components of $L_1 = 1$ and $L_2 = 3$. Together, this analysis provides a theoretical expression to compare simulation and data against, as well as motivation for the use of a two tone fit with frequencies L_1 and L_2 as the expected outcome (Fig. 20).

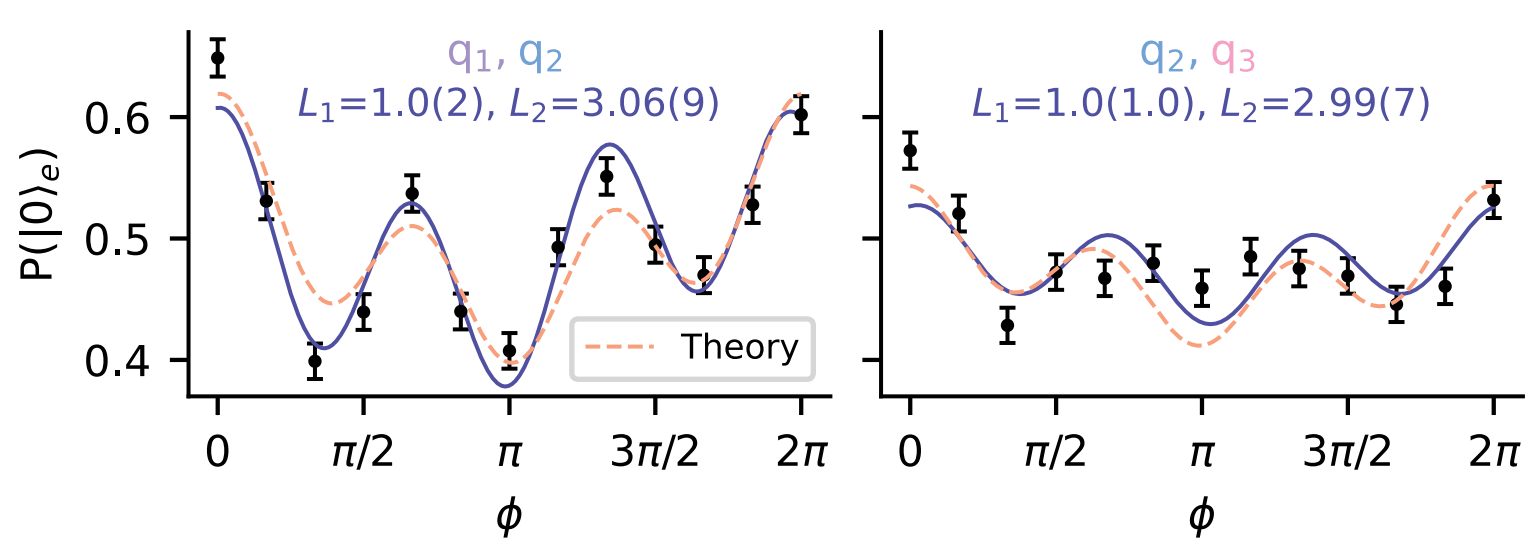

FIG. 20. **Residual entanglement generated with $L = 2$ parallel gates** Theory curve corresponds to equation (62) based on the parallel entangling gate parameters used from table V C. Only the contrast and vertical shift of the theory were adjusted to fit the data sets. In the case of targeting entanglement with nuclear qubits q_2 and q_3, the lower contrast and only one full oscillation resulted in a large uncertainty on L_1. More data could be taken to improve this in the future, but overall the fit (solid blue curve) matches the data well. Data points denote mean values taken over 10^6 shots and error bars denote photon shot noise.

As mentioned in Sec. V C, when targeting entanglement with two nuclear qubits, the next strongest is expected to have the largest residual entanglement generated. In the case of targeting q_1 and q_2, nuclear qubit q_3 acts as the mixed state and when targeting, q_2 and q_3, q_1 acts as the mixed state. Given this and precise knowledge of the hyperfine parameters of each residual nuclear qubit, the crosstalk rotation operators $R_j(\theta)$ can be fixed for each parallel gate unit pulse time and repeats used. This fully specifies equation (49) (Theory curve of Fig. 20) as a function of only ϕ and thus can serve as a prediction for the beating pattern observed in simulation and data. To better visualize this prediction, the contrast and vertical shift of equation (49) were adjusted to match the data in each case. The loss of contrast between the two experiments, and larger uncertainties in the fitting results can be accounted for by the fact that the unit pulse time used for targeting nuclear qubits q_2 and q_3 was farther into the spin bath region. Therefore, additional small amounts of residual entanglement were likely generated with other weaker nuclear qubits that have yet to be measured using DD spectroscopy, or are full unresolvable, and were not accounted for in this analysis.

D. Additional data

In the case of generating entanglement with two of the three possible nuclear qubits, there are three choices of which subset of qubits to target. The main text displayed sequential and parallel MQC results for entanglement with nuclear qubits q_1 and q_2 as an example, leaving pairs q_1, q_3 and q_2, q_3 left to consider (Fig. 21). Since additional data was taken out to 4π to improve frequency fitting results, data for nuclear qubits q_1, q_2 is again shown here in entirety.

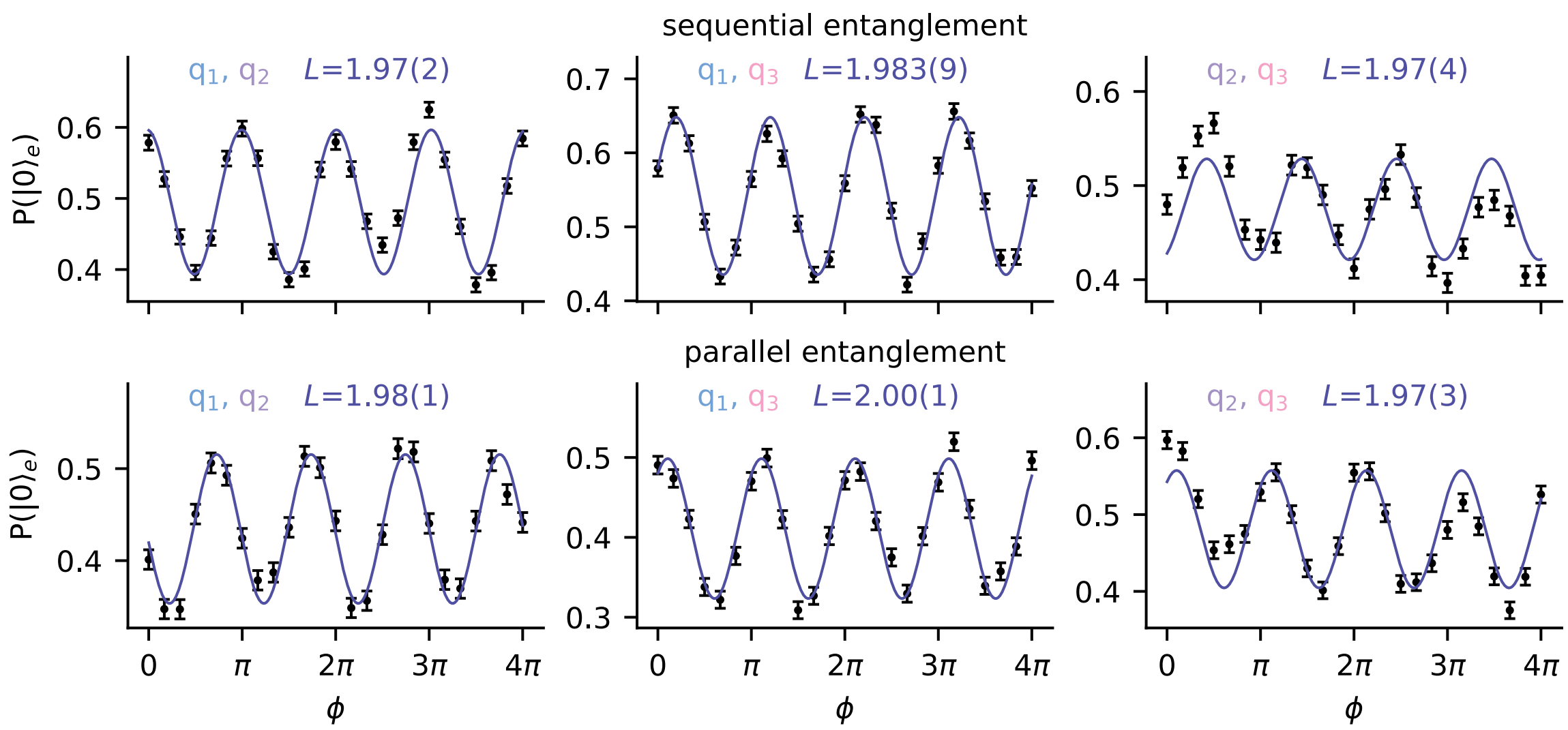

FIG. 21. **Complete $L = 2$ sequential and parallel entanglement data** Entanglement with nuclear qubits q_1 and q_2 was shown in the main text from $\phi = 0$ to 2π. Data points denote mean values taken over 10^6 shots and error bars denote photon shot noise.

X. ENTANGLING GATE FIDELITIES

A. State fidelity expression

The goal of this section is to derive an expression for the quantum state fidelity against $|0\rangle^{\otimes M}$ for an M qubit system in terms of individual qubit measurements. Recall that $M = L + 1$ where L is the number of nuclear qubits in the system. Ultimately, this approach will be an approximation given that correlator measurements need to be made to fully specify any entanglement present between qubits. This point will be discussed at more length once an expression has been derived.

We begin by considering the trace inner product between the two density matrices, which serves as a measure of quantum state fidelity given by:

$$F = \mathrm{tr}\,(\rho_1 \rho_2). \tag{64}$$

Since both ρ_1 and ρ_2 are Hermitian, this expression corresponds to their Frobenius inner product. Let $\rho_1 = \rho$ be any density matrix and $\rho_2 = (|0\rangle\langle 0|)^{\otimes M}$. Since ρ_2 is a projector, we only need to consider a single matrix element of ρ:

$$\begin{aligned} F &= \mathrm{tr}\,(\rho(|0\rangle\langle 0|)^{\otimes M}) \\ &= \mathrm{tr}(\langle 0|^{\otimes M}\rho\,|0\rangle^{\otimes M}) \\ &= \langle 0|^{\otimes M}\rho\,|0\rangle^{\otimes M}. \end{aligned} \tag{65}$$

To proceed, we expand ρ in terms of the M-qubit Pauli basis:

$$\rho = \frac{1}{2^M}\sum_{i_1,\ldots,i_M=0}^{3} c_{i_1,\ldots,i_M}\sigma_{i_1}\otimes\cdots\otimes\sigma_{i_M}, \tag{66}$$

where each coefficient is given by $c_{i_1,\ldots,i_M} = \mathrm{tr}(\sigma_{i_1}\otimes\cdots\otimes\sigma_{i_M}\rho)$. The matrix element of interest can be written as:

$$\begin{aligned} \langle 0|^{\otimes M}\rho\,|0\rangle^{\otimes M} &= \frac{1}{2^M}\sum_{i_1,\ldots,i_M=0}^{3} c_{i_1,\ldots,i_M}\langle 0|\sigma_{i_1}|0\rangle\cdots\langle 0|\sigma_{i_M}|0\rangle \\ &= \frac{1}{2^M}\sum_{i_1,\ldots,i_M=0}^{3} c_{i_1,\ldots,i_M}\prod_{k=1}^{M}\langle 0|\sigma_{i_k}|0\rangle. \end{aligned} \tag{67}$$

Each $\langle 0|\sigma_{i_k}|0\rangle$ is only nonzero for the identity $\mathbb{1}$ $(i=0)$ and Z $(i=3)$, where each quantity is 1, this gives:

$$\begin{aligned} \langle 0|^{\otimes M}\rho\,|0\rangle^{\otimes M} &= \frac{1}{2^M}\sum_{i_1,\ldots,i_M\in\{0,3\}} c_{i_1,\ldots,i_M} \\ &= \frac{1}{2^M}\sum_{i_1,\ldots,i_M\in\{0,3\}} \mathrm{tr}(\sigma_{i_1}\otimes\cdots\otimes\sigma_{i_M}\rho). \end{aligned} \tag{68}$$

Hence, to measure this fidelity exactly, correlators involving different combinations of identity and Z_m operators must be measured. However, since electron-nuclear correlations cannot be measured without single-shot read out, correlator terms such as $\langle Z_e\otimes Z_1 \ldots Z_L\rangle$ have to be avoided to accommodate room temperature conditions. Similarly, swapping inter-nuclear correlations onto the electron is experimentally time consuming and would not provide useful information given the low readout fidelity found in simulation. Given these constraints, we instead derive an approximation for the exact trace fidelity. To this end, we approximate that the state ρ is separable: $\rho = \rho_e\otimes\rho_1\otimes\ldots\otimes\rho_M$. The Pauli decomposition then takes a simpler form now with independent coefficients:

$$\rho = \frac{1}{2^M}\bigotimes_{m=1}^{M}\sum_{i=0}^{3}\langle\sigma_{i_m}\rangle\sigma_{i_m}. \tag{69}$$

Considering only the $\langle 0|^{\otimes M}\rho\,|0\rangle^{\otimes M}$ component for the fidelity expression, we have:

$$\begin{aligned} F &= \langle 0|^{\otimes M}\rho\,|0\rangle^{\otimes M} \\ &= \frac{1}{2^M}\prod_{m=1}^{M}\sum_{i=0}^{3}\langle\sigma_{i_m}\rangle\langle 0|\sigma_{i_m}|0\rangle \\ &= \frac{1}{2^M}\prod_{m=1}^{M}(1+\langle Z_m\rangle), \end{aligned} \tag{70}$$

which means each qubit's Z projection needs to be independently measured.

The difference between the exact state fidelity and the approximate form is determined by correlations within the system. To motivate this, consider the bipartite case where $M=2$. Let F_{ex} be the exact expression of equation (68) and F_a be the approximate expression of equation (70). For this small system size, these are given by:

$$F_{ex} = \frac{1}{2^2}(1+\langle Z_e\rangle+\langle Z_1\rangle+\langle Z_e\otimes Z_1\rangle), \tag{71}$$

and

$$F_a = \frac{1}{2^2}(1 + \langle Z_e \rangle + \langle Z_1 \rangle + \langle Z_e \rangle\langle Z_1 \rangle). \tag{72}$$

Thus, the difference is given by:

$$F_{ex} - F_a = \langle Z_e \otimes Z_1 \rangle - \langle Z_e \rangle\langle Z_1 \rangle \tag{73}$$

Therefore, $F_a \approx F_{ex}$ if the product of the independent measurements is approximately equal to the correlator measurement: $\langle Z_e \rangle\langle Z_1 \rangle \approx \langle Z_e \otimes Z_1 \rangle$. This is true as long as the covariance between the individual observables is near zero. The covariance is defined as:

$$\begin{aligned} \mathrm{Cov}(Z_e \otimes \mathbb{1}, \mathbb{1} \otimes Z_1) &= \mathrm{E}[Z_e \otimes Z_1] - \mathrm{E}[Z_e \otimes \mathbb{1}]\mathrm{E}[\mathbb{1} \otimes Z_1] \\ &= \langle Z_e \otimes Z_1 \rangle - \langle Z_e \rangle\langle Z_1 \rangle, \end{aligned} \tag{74}$$

hence if we assume a covariance near zero, then $\langle Z_e \rangle\langle Z_1 \rangle \approx \langle Z_e \otimes Z_1 \rangle$ as desired. This is exactly equal when there are no correlations, classical or quantum, between the qubits, as expected from the derivation of equation (70) using a separable state. Since our goal is to prepare the state $|00\rangle$ up to the small errors present in the entangling gate, the covariance between observables remains small and the approximation is well justified.

In the case of multipartite entanglement, similar arguments can be made but become more complicated. In order to relate individual measurements to measurements of correlations, one can consider $(L+1)$th order cumulant expansions of observables. To do so, one needs to consider each possible partition of the qubits in the system and analyze the correlations that exist between them. For example, with $L = 2$, the difference in state fidelity is given by

$$\begin{aligned} F_{ex} - F_a =& \langle Z_e \otimes Z_1 \rangle + \langle Z_e \otimes Z_2 \rangle + \langle Z_1 \otimes Z_2 \rangle + \langle Z_e \otimes Z_1 \otimes Z_2 \rangle \\ & - \langle Z_e \rangle\langle Z_1 \rangle - \langle Z_e \rangle\langle Z_2 \rangle - \langle Z_1 \rangle\langle Z_2 \rangle - \langle Z_e \rangle\langle Z_1 \rangle\langle Z_2 \rangle \\ =& \mathrm{Cov}(Z_e, Z_1) + \mathrm{Cov}(Z_e, Z_2) + \mathrm{Cov}(Z_1, Z_2) + \langle Z_e \otimes Z_1 \otimes Z_2 \rangle - \langle Z_e \rangle\langle Z_1 \rangle\langle Z_2 \rangle. \end{aligned} \tag{75}$$

To relate $\langle Z_e \otimes Z_1 \otimes Z_2 \rangle$ and $\langle Z_e \rangle\langle Z_1 \rangle\langle Z_2 \rangle$, one can consider the third order cumulant given by:

$$\mathrm{Cum}(Z_e, Z_1, Z_2) = \langle Z_e \otimes Z_1 \otimes Z_2 \rangle - (\langle Z_e \rangle\langle Z_1 \otimes Z_2 \rangle + \langle Z_1 \rangle\langle Z_e \otimes Z_2 \rangle + \langle Z_2 \rangle\langle Z_e \otimes Z_1 \rangle) + 2\langle Z_e \rangle\langle Z_1 \rangle\langle Z_2 \rangle, \tag{76}$$

where rearranging gives:

$$\begin{aligned} \langle Z_e \otimes Z_1 \otimes Z_2 \rangle - \langle Z_e \rangle\langle Z_1 \rangle\langle Z_2 \rangle = &\mathrm{Cum}(Z_e, Z_1, Z_2) + \left[\langle Z_e \rangle\langle Z_1 \otimes Z_2 \rangle + \langle Z_1 \rangle\langle Z_e \otimes Z_2 \rangle + \langle Z_2 \rangle\langle Z_e \otimes Z_1 \rangle\right] \\ & - 3\langle Z_e \rangle\langle Z_1 \rangle\langle Z_2 \rangle. \end{aligned} \tag{77}$$

Now recast each pairwise correlator $\langle Z_j \otimes Z_k \rangle$ in terms of covariance:

$$\langle Z_j \otimes Z_k \rangle = \mathrm{Cov}(Z_j, Z_k) + \langle Z_j \rangle\langle Z_k \rangle. \tag{78}$$

Therefore:

$$\langle Z_i \rangle\langle Z_j \otimes Z_k \rangle = \langle Z_i \rangle \mathrm{Cov}(Z_j, Z_k) + \langle Z_i \rangle\langle Z_j \rangle\langle Z_k \rangle. \tag{79}$$

Substituting this into equation (77) gives:

$$\begin{aligned} \langle Z_e \otimes Z_1 \otimes Z_2 \rangle - \langle Z_e \rangle\langle Z_1 \rangle\langle Z_2 \rangle &= \mathrm{Cum}(Z_e, Z_1, Z_2) + \big[\langle Z_e \rangle \mathrm{Cov}(Z_1, Z_2) + \langle Z_1 \rangle \mathrm{Cov}(Z_e, Z_2) + \langle Z_2 \rangle \mathrm{Cov}(Z_e, Z_1) \\ &\quad + 3\langle Z_e \rangle\langle Z_1 \rangle\langle Z_2 \rangle\big] - 3\langle Z_e \rangle\langle Z_1 \rangle\langle Z_2 \rangle \\ &= \mathrm{Cum}(Z_e, Z_1, Z_2) + \langle Z_e \rangle \mathrm{Cov}(Z_1, Z_2) + \langle Z_1 \rangle \mathrm{Cov}(Z_e, Z_2) + \langle Z_2 \rangle \mathrm{Cov}(Z_e, Z_1). \end{aligned} \tag{80}$$

Hence, if each covariance and the three way cumulant are approximately zero, we have our desired outcome:

$$\langle Z_e \otimes Z_1 \otimes Z_2 \rangle \approx \langle Z_e \rangle\langle Z_1 \rangle\langle Z_2 \rangle. \tag{81}$$

Again, since the target state contains no correlations, if this is prepared up to small errors present in the system, then each covariance and the three way cumulant are approximately zero and the fidelity approximation holds well.

Proceeding to larger L system sizes, the argument extends similarly, but requires that each $(L-1)$th order cumulant remain near zero. Ultimately, as long as we can prepare states close to $|0\rangle^{\otimes M}$ without inducing strong correlations in the system, we can expect the individual measurement state fidelity (equation (70)) to serve as an approximation for the exact state fidelity (equation (68)).

B. Entangling gate repeat sampling

Now that we have an expression for the state fidelity in terms of independent qubit measurements that can readily be made experimentally, we need to determine which number of repeats of the entangling gates N_E are expected to return the system to $|0\rangle^{\otimes M}$, up to the error associated with the gate. This will reveal which number of repeats we should measure at and then use equation (70) on the outcomes. From these state fidelities, the decay of state fidelity will be fit to an noisy quantum channel model to extract the error per gate, or equivalently the entangling gate fidelity (more details in Sec. X C)

1. Bipartite and sequential gates

In the bipartite entanglement case, determining the number of entangling gate repetitions, N_E, required to return the initialized system to $|00\rangle$ is straightforward. Since the gate geometry is $CX_{\pi/2}$, then multiples of four repeats create $CX_{2\pi k} = \mathbb{1} \otimes \mathbb{1}$ for $k \in \mathbb{Z}$. More generally, odd values of N_E produce maximally entangled states, while even values results in separable states. Although N_E values of resolution one are not needed to calculate the entangling gate's fidelity, full data was taken to highlight the gate's geometry described above and is shown in Fig. 22. From these data sets, only N_E values divisible by four were used to calculate the $|00\rangle$ state fidelity according to equation (70) and are shown in the main text. When the pair of qubits is maximally entangled, each qubit's Z projection is expected to be zero since each qubit is in a maximally mixed state when traced out (orange "Max entangled" points of Fig. 22).

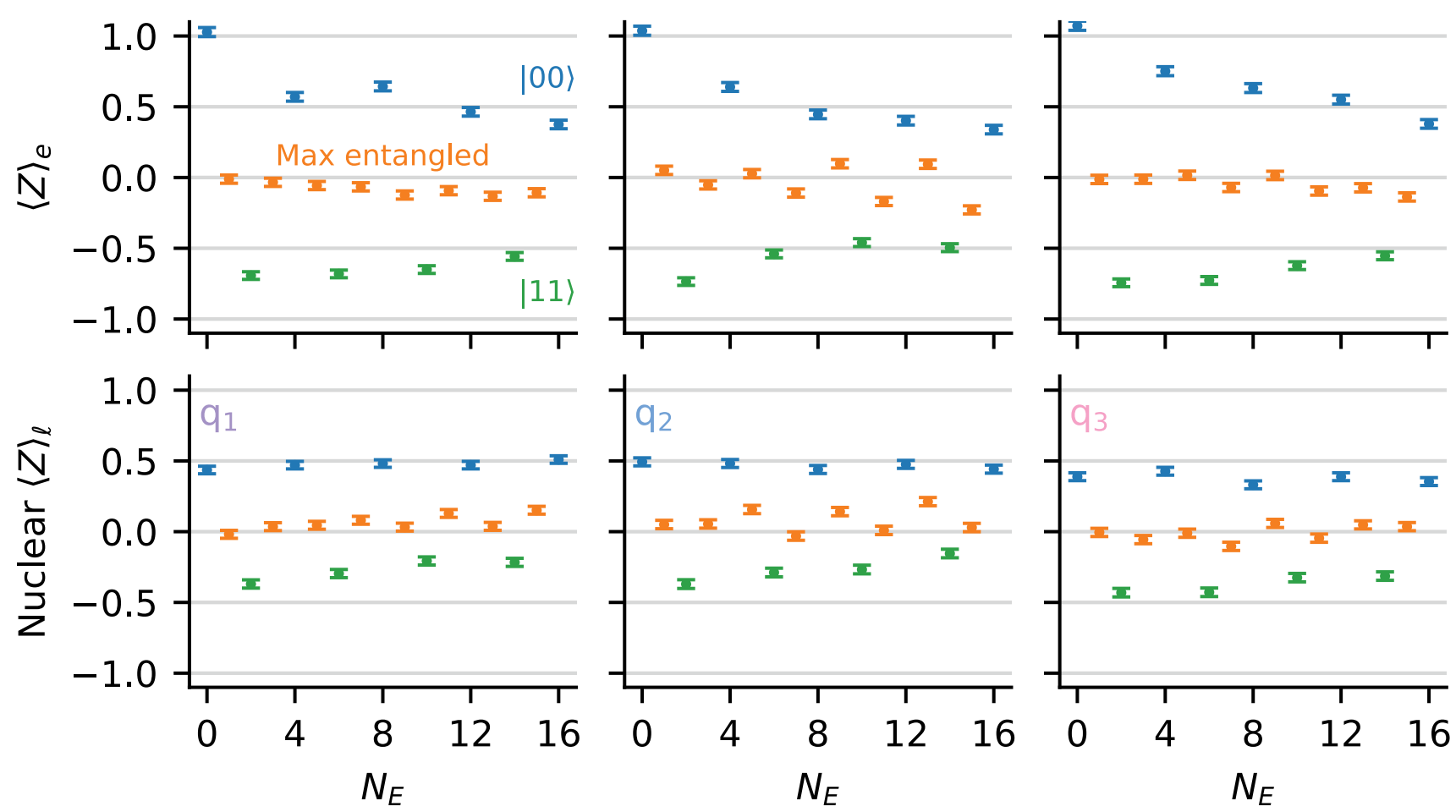

FIG. 22. **Full bipartite entangling gate data** Each plot is a single, distinct data set with the individual points color coded to highlight their meaning and role in the analysis. Each column is a distinct bipartite entangling gate repeated N_E times and each row corresponds to whether the electron or targeted nuclear qubit was measured. Only the blue "$|00\rangle$" points where $N_E \equiv 0(\text{mod}4)$ are used in calculating each bipartite entangling gate fidelity in the main text. Data points denote mean values taken over 10^6 shots and error bars denote photon shot noise.

Similarly, when using sequences of two-qubit $CX_{\pi/2}$ gates to generate multipartite entanglement, it is expected that N_E values divisible by four should return the system to the state $|0\rangle^{\otimes M}$. Sampling at these values compares the M-qubit sequential entangling gate against the ideal version of $CX_{\pi/2}^{\otimes L}$. As discussed, crosstalk is a significant factor in preventing the sequential approach from behaving like this ideal gate with high fidelity. Thus using N_E values divisible by four directly probe the crosstalk errors associated with it that do not affect the bipartite entangling cases.

2. Parallel gates

In the parallel entanglement case, determining the number of entangling gate repeats required to return the initialized system to $|0\rangle^{\otimes M}$ is more challenging given each gate's complicated geometry. In order to determine these values, an ideal simulation of each parallel gate was used to examine the role of its geometry is isolation. This simulation idealized register initialization, electron gate errors, measurement of correlators and only included the nuclear qubits

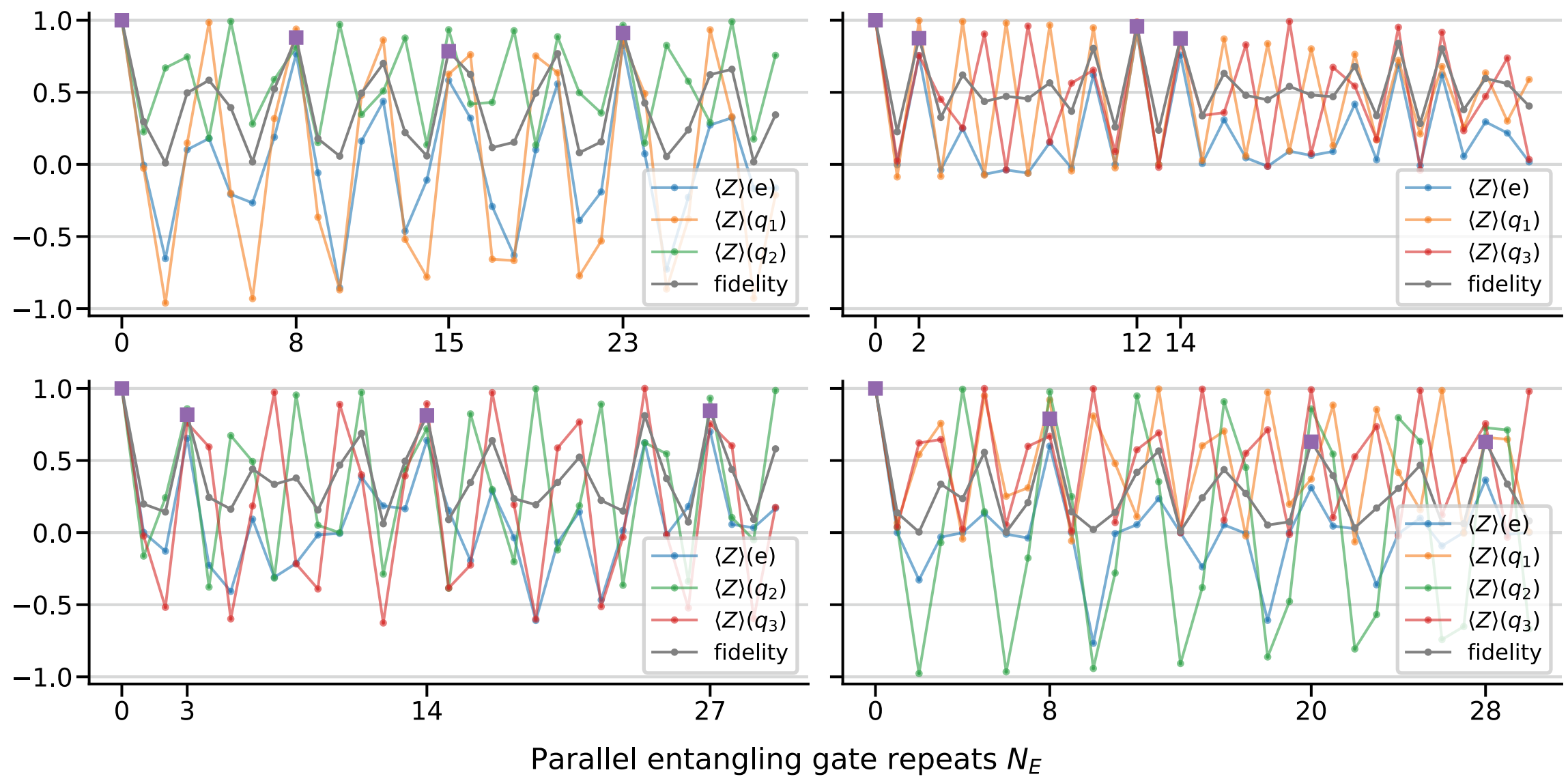

FIG. 23. **Idealized parallel entangling gates repeats** Z projections of each qubit, and exact state fidelity, from an ideal simulation of the $M = 3$ and $M = 4$ parallel entangling gates repeated N_E times. The four values of N_E that came closest to returning the state of the system to $|0\rangle^{\otimes M}$ (purple square markers) were chosen as points to experimentally sample at.

there were targeted for entanglement (Fig. 23). From this, the four values of N_E that came closet to return the system to $|0\rangle^{\otimes M}$ were chosen as sample points. To account for the small difference in the exact states produced, the ideally produced density matrices at each N_E were used an initial states in the fitting procedure described in the following section.

C. Kraus operator error model

In order to extract the entangling gate fidelity from the decay of the state fidelity measurements, a bit-flip quantum channel was developed and fit to the data. In this quantum channel, the probability of an individual bit-flip caused by the entangling gate is given by $\varepsilon \in [0, 1]$ and thus the probability of maintaining the bit without error is given by $1 - \varepsilon$. For M qubits, there are 2^M total possible bit-flip combinations, each represented with their own Kraus operator K_i. Thus, for k bit-flips where $0 \leq k \leq M$, the probability amplitude of the corresponding Kraus operator is given by:

$$c_{M,k}(\varepsilon) = \sqrt{\varepsilon^k (1 - \varepsilon)^{M-k}}. \tag{82}$$

This is the coefficient of the Kraus operator with k Pauli X operators, representing the bit-flips, tensored with $M - k$ identity operators. For k bit-flips, there are $n_{M,k} = \binom{M}{k}$ many Kraus operators with k Pauli X operators present among the M components of the Hilbert space. This fact is useful in proving the trace preservation of this channel $\sum_i K_i^\dagger K_i = I$, which relies on the sum of the squares of the probability amplitudes to be 1:

$$\begin{aligned} \sum_{\text{Kraus ops}}^{2^M} c_{M,k}^2(\varepsilon) &= \sum_{k=0}^{M} n_{M,k} c_{M,k}^2(\varepsilon) \\ &= \sum_{k=0}^{M} \binom{M}{k} \varepsilon^k (1 - \varepsilon)^{M-k} \\ &= (1 - \varepsilon + \varepsilon)^M \\ &= 1 \end{aligned} \tag{83}$$

For example, in the $M = 2$ bipartite case, the four Kraus operators are given by:

$$\begin{aligned} K_1 &= \sqrt{(1-\varepsilon)^2} I \otimes I \\ K_2 &= \sqrt{\varepsilon(1-\varepsilon)} I \otimes X \\ K_3 &= \sqrt{\varepsilon(1-\varepsilon)} X \otimes I \\ K_4 &= \sqrt{\varepsilon^2} X \otimes X \end{aligned} \tag{84}$$

In all M qubit cases, the squared coefficient of the Kraus operator containing only identities is what provides the gate fidelity measurement. Based on equation (82), the gate fidelity G_M is therefore given by:

$$\begin{aligned} G_M &= c^2_{M,0}(\varepsilon) \\ &= (1-\varepsilon)^M. \end{aligned} \tag{85}$$

Thus, based on the least squares fitting of the gate's error rate $\varepsilon_{\text{gate}}$ of this quantum channel to the state fidelities measured, the entangling gate fidelities were calculated with equation (85).

To account for constant SPAM errors, one application of this quantum channel was applied to an initial of state with a free parameter error rate $\varepsilon_{\text{SPAM}}$. This initial state was $|0\rangle^{\otimes M}$ for the bipartite and sequential entangling gates, and the ideally simulated states nearest $|0\rangle^{\otimes M}$ from Fig. 23 for the parallel entangling gates. The use of the ideally simulation states for the parallel gate allowed the channel to not penalize the gate for producing states that it was unable to due to its geometry. Then, using these imperfect initial density matrices, the quantum channel with a free parameter $\varepsilon_{\text{gate}}$ was applied N_E times recursively for each entangling gate repeat sampled experimentally. Based on the model's final density matrix, the state fidelity against $|0\rangle^{\otimes M}$ was then calculated for each N_E and fit to the state fidelities measured using least squares.

The only data sets not shown in the main text were the other $L = 2$ nuclear qubit combinations of q_1, q_2 and q_2, q_3 (Fig. 24) Note, the sampling of the fits is larger (resolution of 1 repeat) for the sequential entangling gates since a constant initial density matrix of $|000\rangle$ was used. In the parallel case, since a unique ideal density matrix nearest $|000\rangle$ was taken from simulation for each N_E, a finer resolution could not be meaningfully used in displaying the fitting results. The entirety of the fitting results from the data sets in the main text and Fig. 24, including the SPAM fidelity, is shown in Table X C.

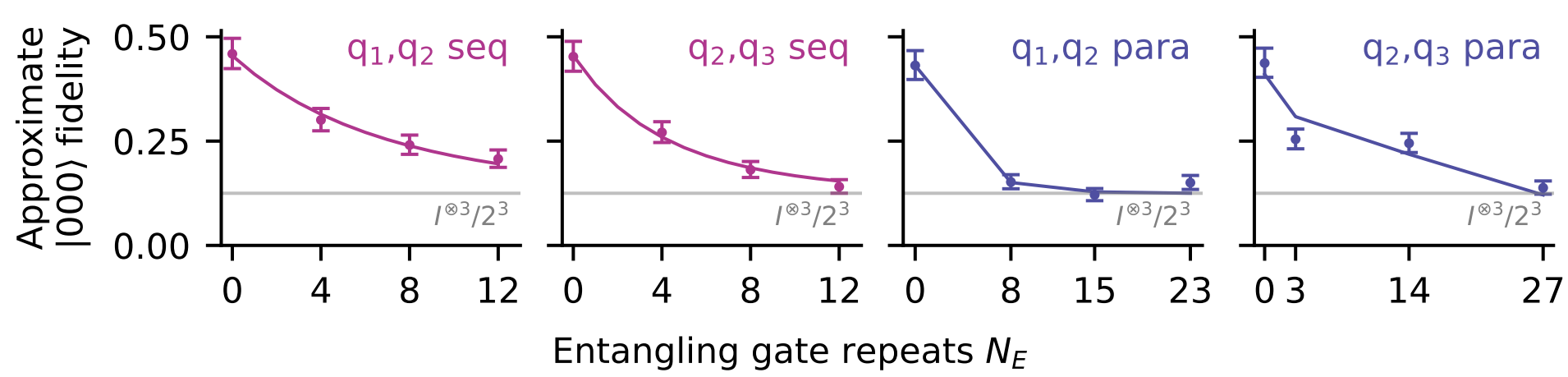

FIG. 24. **Additional $L = 2$ entangling gate fidelity data** Solid lines denote fits to noisy quantum channel described in Sec. X C. Data points denote mean values taken over 10^6 shots and error bars denote photon shot noise.

To give some insight into the nature of using approximate state fidelities to calculate the gate fidelities, the state fidelities created by the parallel entangling gates were calculated using both the exact trace fidelity as in equation (68) and the approximate fidelity in equation (70). The difference in fidelities depends on the extent to which correlations exist within the system and subsystems (see equations (73) and (75) for exact expressions). Fig. 25 reveals that the approximate state fidelity is always less than the exact, with the difference approximately growing larger as a function of N_E. A similar behavior holds for the same behavior holding for the sequential entangling gates. Thus, the state fidelities calculated experimentally according to the approximate fidelity decrease faster as a function of N_E than the exact values and therefore the quantum channel fit to the data produces a lower entangling gate fidelity. This observation suggests that the experimental entangling gate fidelities we obtain actually underestimate the exact values.

XI. APPLICATIONS TO QUANTUM ERROR CORRECTION

Following the quantum circuits of Takou *et al.* [4] and the experimental techniques of Taminiau *et al.* [6], we simulate how the parallel entangling gates used in this work could improve quantum error correction protocols. In

Entangling gate	SPAM fidelity	Gate fidelity
q_0	0.70 ± 0.04	0.972 ± 0.008
q_1	0.73 ± 0.03	0.956 ± 0.008
q_2	0.71 ± 0.02	0.963 ± 0.004
q_1, q_2 sequential	0.46 ± 0.01	0.86 ± 0.01
q_1, q_2 parallel	0.43 ± 0.02	0.74 ± 0.08
q_1, q_3 sequential	0.48 ± 0.03	0.69 ± 0.06
q_1, q_3 parallel	0.48 ± 0.02	0.80 ± 0.04
q_2, q_3 sequential	0.46 ± 0.01	0.79 ± 0.02
q_2, q_3 parallel	0.41 ± 0.04	0.94 ± 0.04
q_1, q_2, q_3 sequential	0.31 ± 0.01	0.69 ± 0.03
q_1, q_2, q_3 parallel	0.32 ± 0.01	0.92 ± 0.04

TABLE XI. **Gate and SPAM fidelity fit results.** The SPAM fidelity decreases for larger registers due to the combined effects of reduced nuclear initialization and measurement fidelity.

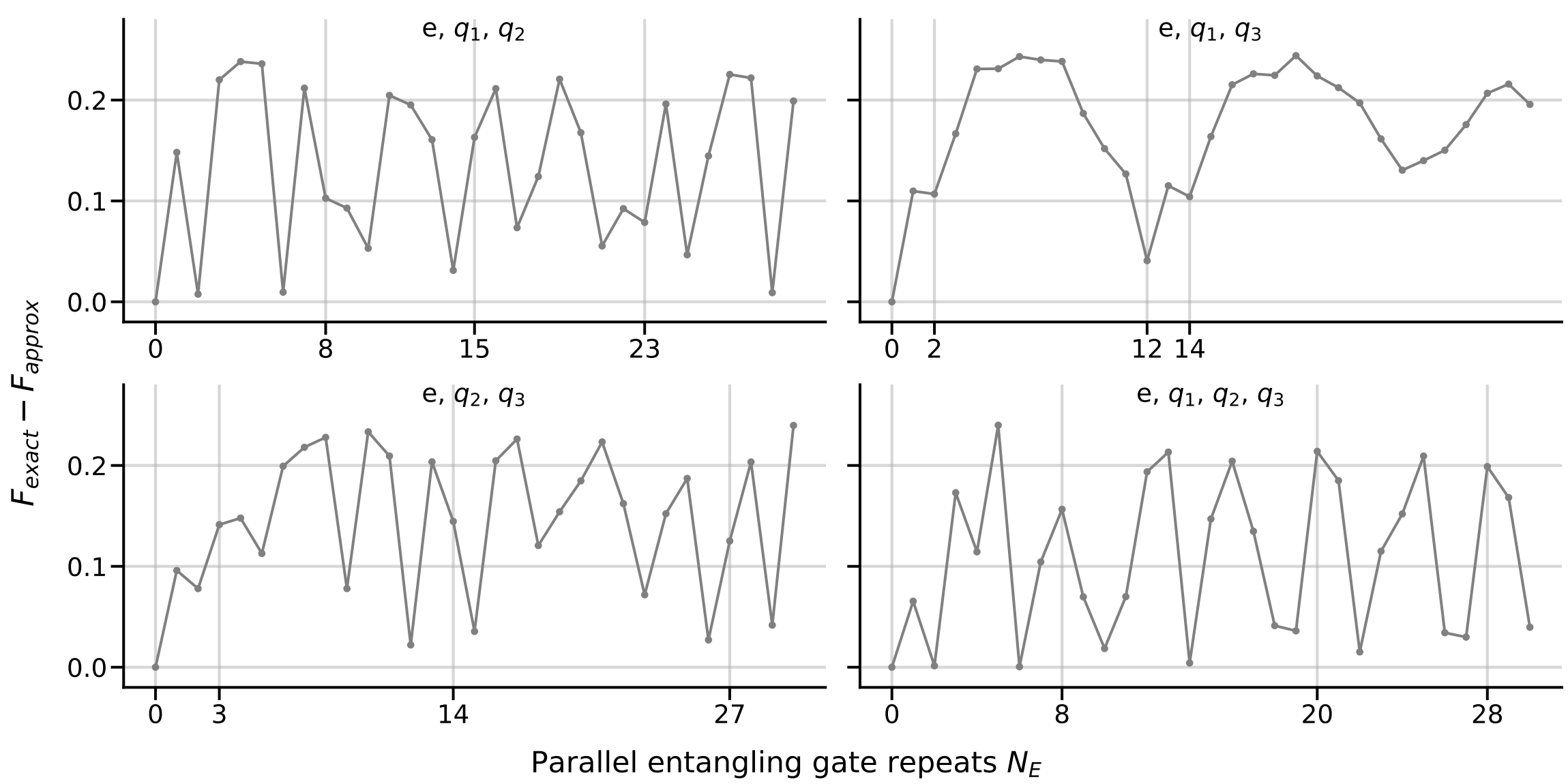

FIG. 25. **Difference between the exact and approximate state fidelities.** F_{exact} is calculated based on equation equation (68) and F_{approx} is calculated based on equation (70).

this example, a three-qubit bit-flip code is simulated that can detect and correct any single qubit bit-flip errors by majority voting. The protocol is simulated two ways — one using a sequence of two-qubit gates in the encoding and decoding steps (Fig. 26a), and the other using a single parallel entangling gate (Fig. 26b). The correction is implemented using a doubly controlled not (Toffoli) gate, which is idealized in the following simulations to accentuate only the differences in the entangling gates used during encoding and decoding. The Toffoli gate can be implemented with a sequence of single and two-qubit DD gates, but is therefore subject to their crosstalk. Similarly, in both the parallel and sequential cases, register initialization is idealized and electron gate errors associated with DD sequences are ignored. Single qubit errors are induced on the electron as a variable X_θ rotation. The correction is turned off by flipping one of the nuclear qubits at the start of the circuit (keeping it in $|0\rangle$ at the start). In the parallel case, additional local Y_π gates are needed on the nuclear qubits to compensate for the complicated geometry of the parallel gate [4], also idealized in the following simulation. The electron is prepared in 6 initial states $|\psi\rangle = |\pm X\rangle, |\pm Y\rangle, |\pm Z\rangle$, from which the process fidelity as a function of error angle is calculated according to [6] (Fig. 26c). Notably, the parallel gate maintains approximately double the process fidelity than the sequential entangling gates over all error angles — which is directly related to its higher fidelity. With no bit-flip error on the electron, the parallel gate still does not reach unit process fidelity with idealized local nuclear gates due to residual entanglement generated by the next strongest nuclear qubit. Parallel gates with lower amounts of residual entanglement will behave even closer to the idealized blue curve of Fig. 26c. Improvements to such QEC circuits with the parallel gate can immediately benefit recent studies of hybrid entanglement between the electron, multiple ^{13}C nuclear qubits, and photons at low temperatures for error corrected quantum networking applications [14].

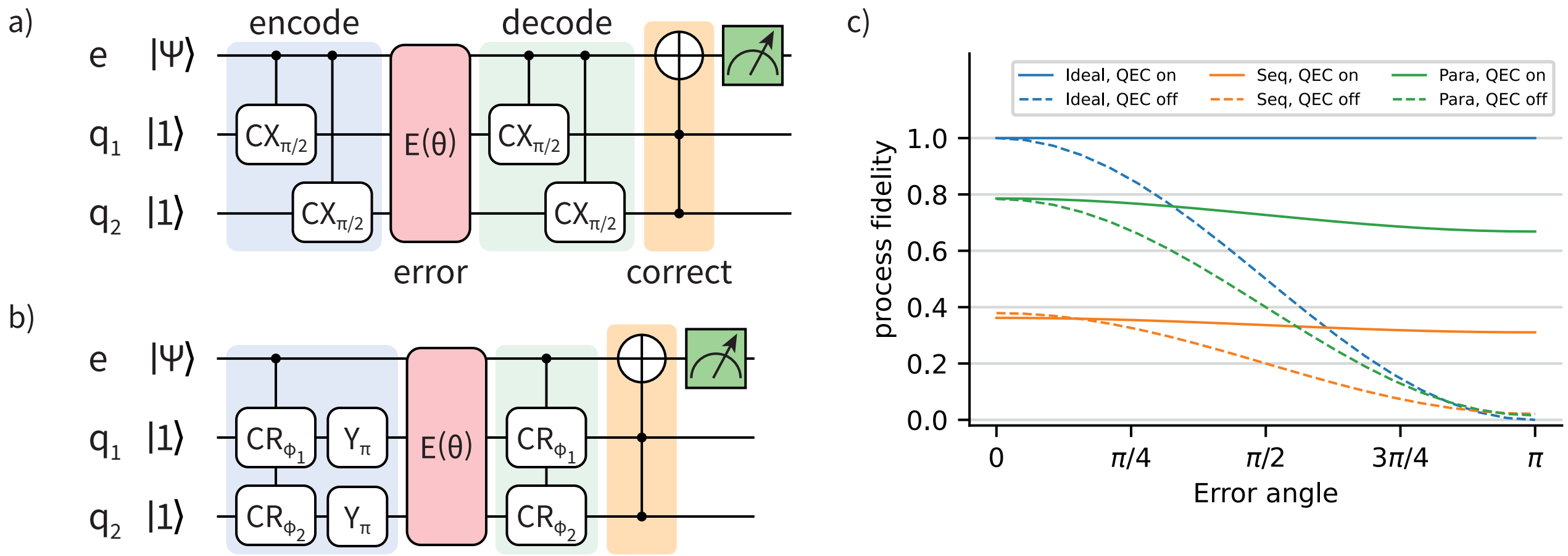

FIG. 26. **Three-qubit bit-flip code (a)** bit-flip code using sequences of two-qubit gates. **(b)** bit-flip code using parallel three-qubit gate. Local nuclear Y_π gates are needed to compensate for entangling gate geometry. **(c)** Process fidelity of correcting bit-flip error on the electron qubit. "QEC on" refers to the initial nuclear state $|11\rangle$, while "QEC off" refers to the initial nuclear state $|01\rangle$ (equivalently, $|10\rangle$). Since the code cannot correct two bit-flips, the flipped nuclear qubit deactivates the code.

While three-qubit bit (and phase) flip codes are useful for single qubit errors, they fail to correct multiple errors and are overall not fault-tolerant. With additional nuclear qubits, minimal fault-tolerant protocols can be achieved with parallel entangling gates using a similar approach as in Fig. 26b. The first fault-tolerant operation of a NV-nuclear qubit register was demonstrated using $L = 5$ weakly coupled ^{13}C nuclear qubits, as well as the ^{14}N qubit as a flag qubit [15]. The encoding and decoding steps to form the logical qubit required a sequence of two-qubit gates, dominating the circuit depth. Based on the simulations of Sec. VI, $L = 5$ parallel entangling gates with properties similar to the ones demonstrated in this work are possible in a small, but reasonable, fraction of NV centers. Searching for such an NV center could drastically reduce circuit durations and errors, potentially enabling fault tolerance at room temperature for the first time.

[1] VV Dobrovitski, G De Lange, D Riste, and R Hanson. Bootstrap tomography of the pulses for quantum control. *Physical Review Letters*, 105(7):077601, 2010.

[2] HP Bartling, J Yun, KN Schymik, M van Riggelen, LA Enthoven, HB van Ommen, M Babaie, F Sebastiano, M Markham, DJ Twitchen, and TH Taminiau. Universal high-fidelity quantum gates for spin-qubits in diamond. *Physical Review Applied*, 23(3):034052, 2025.

[3] Lee C Bassett, F Joseph Heremans, David J Christle, Christopher G Yale, Guido Burkard, Bob B Buckley, and David D Awschalom. Ultrafast optical control of orbital and spin dynamics in a solid-state defect. *Science*, 345(6202):1333–1337, 2014.

[4] Evangelia Takou, Edwin Barnes, and Sophia E Economou. Precise control of entanglement in multinuclear spin registers coupled to defects. *Physical Review X*, 13(1):011004, 2023.

[5] T Van der Sar, ZH Wang, MS Blok, H Bernien, TH Taminiau, DM Toyli, DA Lidar, DD Awschalom, Robin Hanson, and VV Dobrovitski. Decoherence-protected quantum gates for a hybrid solid-state spin register. *Nature*, 484(7392):82–86, 2012.

[6] Tim Hugo Taminiau, Julia Cramer, Toeno van der Sar, Viatcheslav V Dobrovitski, and Ronald Hanson. Universal control and error correction in multi-qubit spin registers in diamond. *Nature Nanotechnology*, 9(3):171–176, 2014.

[7] TH Taminiau, JJT Wagenaar, T Van der Sar, Fedor Jelezko, Viatcheslav V Dobrovitski, and R Hanson. Detection and control of individual nuclear spins using a weakly coupled electron spin. *Physical Review Letters*, 109(13):137602, 2012.

[8] Zhi-Hui Wang, G De Lange, D Ristè, R Hanson, and VV Dobrovitski. Comparison of dynamical decoupling protocols for a nitrogen-vacancy center in diamond. *Physical Review B*, 85(15):155204, 2012.

[9] Evangelia Takou, Edwin Barnes, and Sophia E Economou. Generation of genuine all-way entanglement in defect-nuclear spin systems through dynamical decoupling sequences. *Quantum*, 8:1304, 2024.

[10] Conor E Bradley, Joe Randall, Mohamed H Abobeih, Remon C Berrevoets, Maarten J Degen, Michiel A Bakker, Matthew Markham, Daniel J Twitchen, and Tim H Taminiau. A ten-qubit solid-state spin register with quantum memory up to one minute. *Physical Review X*, 9(3):031045, 2019.

[11] Paul L Stanwix, Linh M Pham, Jeronimo R Maze, David Le Sage, Tsun Kwan Yeung, Paola Cappellaro, Philip R Hemmer,

Amir Yacoby, Mikhail D Lukin, and Ronald L Walsworth. Coherence of nitrogen-vacancy electronic spin ensembles in diamond. *Physical Review B*, 82(20):201201, 2010.

[12] Ilai Schwartz, Jochen Scheuer, Benedikt Tratzmiller, Samuel Müller, Qiong Chen, Ish Dhand, Zhen-Yu Wang, Christoph Müller, Boris Naydenov, Fedor Jelezko, et al. Robust optical polarization of nuclear spin baths using Hamiltonian engineering of nitrogen-vacancy center quantum dynamics. *Science Advances*, 4(8), 2018.

[13] David A Hopper, Joseph D Lauigan, Tzu-Yung Huang, and Lee C Bassett. Real-time charge initialization of diamond nitrogen-vacancy centers for enhanced spin readout. *Physical Review Applied*, 13(2):024016, 2020.

[14] Xiu-Ying Chang, Pan-Yu Hou, Wen-Gang Zhang, Xiang-Qian Meng, Ye-Fei Yu, Ya-Nan Lu, Yan-Qing Liu, Bin-Xiang Qi, Dong-Ling Deng, and Lu-Ming Duan. Hybrid entanglement and bit-flip error correction in a scalable quantum network node. *Nature Physics*, 21(4):583–589, 2025.

[15] Mohamed H Abobeih, Yu Wang, John Randall, SJH Loenen, Christina E Bradley, Matthew Markham, Daniel J Twitchen, Barbara M Terhal, and Tim H Taminiau. Fault-tolerant operation of a logical qubit in a diamond quantum processor. *Nature*, 606(7916):884–889, 2022.
