# Supplementary material for: Single-gate, multipartite entanglement on a room-temperature quantum register
Source: Nat Nanotechnol. 2026 Sep 14;21(10):1383–9. doi: 10.1038/s41565-026-02254-6 (PMC13661294; doi:10.1038/s41565-026-02254-6)
Supplement: Supplementary file 2 [file 41565_2026_2254_MOESM2_ESM.pdf]

nature portfolio

Peer Review File

Single-gate, multipartite entanglement on a room-temperature quantum register

Corresponding Author: Professor Lee Bassett

This file contains all reviewer reports in order by version, followed by all author rebuttals in order by version.

A version of this paper was originally rejected for publication by Nature Nanotechnology, however that decision was reconsidered after appeal by the authors.

Version 0:

Reviewer comments:

Reviewer #1

(Comments for the Author)
Summary:
The paper by Minnella et al. reports on the experimental demonstration of a single parallel dynamical-decoupling (DD) gate that generates a four-qubit GHZ state (the NV electron plus three proximal 13-C nuclei) at room temperature. In the more well-known method of generating GHZ states, on a previous device with similar hyperfine coupling values, such an entangling sequence was composed of sequential DD-sequences for each nuclear spin. In this work, the authors introduced a faster method of entanglement by leveraging the crosstalk from the DD gates due to similar hyperfine coupling values.

Using this multi-qubit gate they generated a 4-qubit GHZ state approximately 10 times faster than if they were to use the sequence of two-qubit gates, reducing their entangling gate operation time from 139.9 μs to 14.8 μs and leading to an improvement in gate fidelity from 0.69(3) to 0.96(1). While existing limitations remain dominated by room-temperature SPAM errors and errors from the local hyperfine landscape, they conclude that such a multi-qubit DD gate is well-positioned for the scalable generation and control of entanglement in solid-state registers for both error correction and entanglement-enhanced quantum sensing.

Three key questions stand out: 1. how is this technique applicable to other qubit platforms, and indeed to other devices in the same system, where it is not so easy to achieve similar hyperfine couplings values? 2. For scalability other limitations still dominate such as SPAM errors and local hyperfine variations, can the authors address this in their argument for scalability and 3. They have not demonstrated or discussed in detail how this will be used for quantum error correction and this should be addressed.

Originality and significance
The realization of multi-qubit gates in solid-state spin registers is not in itself new. For example, multi-qubit entangling gates have been previously demonstrated in nitrogen vacancies (see Waldherr et al, Nature 506, 204-207 (2014)) and later in silicon with fidelities up to 99.9% (see H. Edlbauer et al., arXiv:2506.03567v1 and I. Thorvaldson et al., Nature Nanotechnology 20, 472-477 (2025)). Note that, these previous results leverage the geometric phase of the electron instead of dynamical decoupling (DD) gate sequences.

Nuclear control utilising dynamically decoupling pulses of the electron has been studied in many previous works in nitrogen vacancy systems (such as T. H. Taminiau et al, Nature Nanotechnology 9, 171-176 (2014)). While earlier techniques primarily focused on sequential operation of the nuclear spins, recent theoretical work by Takou et al (Phys. Rev. X 13, 011004 (2023) & Quantum 8, 1304 (2024)) has shown that it is possible to perform fully entangling operations over multiple nuclei, provided they are similar enough in resonance frequency. The result shown by Minnella et al represents, to our knowledge, the first experimental demonstration of such proposed pulses, characterising the fidelity of these operations on a physical device. Due to the slower operations needed to individually address nuclei with similar hyperfine coupling values, the result presented does indeed show a 10x speedup (from 139.9us to 14.8us) for simultaneous GHZ entanglement compared to sequential entanglement. However, it is important to note that other devices, where the nuclear spins are more addressable (see Edlbauer et al, Thorvaldson et al, Waldherr et al, Taminiau et al above) observe entanglement times of

~10-30us, meaning that the techniques demonstrated by Minnella et al seem to be only specific to devices where the ability to address individual nuclei is challenging. It would be good for the authors to comment on this.

Overall, we believe that the results shown by Minnella et al are novel, in that they are the first experimental demonstration of simultaneous entanglement in solid state systems using dynamical decoupling techniques. This technique provides an experimentally verified alternative to the existing dynamical-decoupling control techniques which are well-established in the solid-state qubit field. We believe that in some unique cases, where hyperfine couplings are similar, this may result in both faster device operation and higher operational fidelities. It would be good for the authors to comment on how wide-spread this technique would be available. We can see that the results were obtained at room temperature, but this technique should be applicable to devices operated at cryogenic temperatures.

Regarding the significance of these results, the main concern therefore is to the extent this method is applicable to future devices which can have different hyperfine coupling strengths and number of nearby nuclear spins. It would be helpful if the authors can quantify the influence of the variation in hyperfine coupling on speed and simulated gate fidelity.

In addition, the authors mentioned the benefit of such an entangling gate for quantum error correction (QEC) schemes. However, the authors did not mention which QEC method would benefit from it since traditional surface code would require multi-qubit gate for a subset of data qubits at the time, instead of operating on all qubits at once.

Data and methodology
Overall, the data and methodology appear solid and are generally aligned with established techniques in NV-center quantum control. The authors rely heavily on simulation to identify optimal DD parameters (such as t and N for multi-qubit entangling gate), including the compensation phases for local gates. However, some methodological choices and omissions require clarification, and several figures would benefit from additional contextual information or consistency in presentation.

Figure 2
The Multiple Quantum Coherence (MQC) method is correctly cited (Refs. 42–43). The data are fit with Eq. 2 as expected, although it may be helpful for the reader to mentioned it in the figure caption.

Figure 3
The parallel entanglement method is correctly attributed to Refs. 40–41. The data is fitted with theory and additional data sets are shown in the supplementary information, although the fitted phase compensation parameters are not shown.

An important point to raise is the key results shown in fig3c. The oscillation may have a secondary frequency (similar to fig 3e). What does this plot look like if a second frequency is included? In addition, the uncertainty in L for fig 3c is smaller than fig 3d, even though the latter seems to have a cleaner oscillation pattern. If the authors can comment on these points, it will be strengthening their conclusion.

Figure 4
The methodology adapted from Evered et al. (Ref. 36) seems appropriate, and separating SPAM from gate error is critical given the room-temperature constraints. The use of a quantum depolarizing channel to fit fidelity decay is reasonable. The choice of N_E for parallel gates is shown in the supplementary information.
One concerning point is that the authors assume there is no correlated errors in the final state, and therefore they can apply their model to extract the gate fidelity. However, it will strengthen their conclusion if they quantify the extent of the correlated errors.

Appropriate use of statistics
The data points have error bars, although it is missing how these error bars are calculated (probably from number of repetitions). On the other hand, the uncertainties from the fitted values are presented. Overall, additional explanations on how these statistics are calculated would be appreciated.

Conclusions, robustness, validity and reliability
For the current device, these results seem to be valid, and in good agreement with the simulations. However, there are a few key points that the authors should address to strengthen the impact of this work.
1. As mentioned previously, the main concern is up to which extent this method is applicable to future devices. In other words, are there any analytical conditions on relevant parameters such as hyperfine coupling strengths, t, N, number of nearby nuclei that these gates perform at high fidelity?
2. It appears that uniform separations (i.e. near-equal spacings of hyperfine couplings at 40 kHz) simplifies finding a single t and N that addresses all targets. If hyperfine couplings are asymmetrically spaced and/or separated larger in their spacing, how does this affect the implementation of the DD gate?
3. Similarly, as shown in Fig 1c, if L=4, is it still possible to apply this method?
4. Since a requirement for this method is to have (n0 · n1) < 0, how does the gate fidelity change if (n0 · n1) becomes close to zero? Does this limit the observed gate fidelity?

References: appropriate to credit existing work
Overall, the paper by Minnella et al is well-referenced, with relevant existing work credited where appropriate. However, when reviewing the previous work performed in solid-state spin registers, we believe Minnella et al omitted important work that utilises geometric phase gates to efficiently create multi-qubit entangling gates. This work was originally performed in

nitrogen vacancy centres (Waldherr et al, Nature 506, 204-207 (2014)), and has been leveraged extensively in recent work in phosphorus atom qubits in silicon (see M. T. Mądzik et al, Nature 601, 348-353 (2022); H. Edlbauer et al., arXiv:2506.03567v1 (2025); and I. Thorvaldson et al., Nature Nanotechnology 20, 472-477 (2025)). Despite these operations being performed with different techniques to those explored by Minnella et al, we believe that this past work should be referenced in their manuscript.

Clarity and context
In the main text, the "order" k was somewhat unclear on first reading (specifically in main text section I and the caption of Fig. 1). Can the authors please add a definition (or reference) to the main text to define this variable? If it is the same k appearing in methods section B, then an explicit reference to this would make it much clearer.

In addition, we believe that the caption of Fig. 2a would be made clearer if the authors explicitly stated that the circuit in 2a was not used for the analysis performed in the rest of the paper – instead, the Z rotation of the electron was omitted. This would avoid confusion as 2a suggests oscillations should appear at a frequency given by M, in contrast to the experimental results which oscillate at a frequency of L.

Reviewer #2

(Comments for the Author)
In Minnella et al, the authors demonstrate a scheme to perform multipartite entanglement between a nitrogen vacancy center electron spin and three nearby 13C nuclear spins using a single entangling gate at room temperature. The multipartite entanglement uses a scheme that was theoretically developed by Takou et al. (refs 40, 41) that leverages the inherent crosstalk present in the always-on hyperfine interactions between electron and nuclear spins to generate maximally entangled states. The authors then generate three- and four-qubit GHZ states and adapt a method (Wei et al. PRA 2020) to verify the number of entangled spins by measuring multiple quantum coherences. They then estimate the fidelity of the four-qubit GHZ state (using a method adapted from Evered et al. Nature 2023), and show that the GHZ state preparation enabled by the single gate is faster and higher fidelity than the state preparation via serial single gates.

Prior work in the field has relied on such serial single gates with a central electron spin to enable generation of multiparticle entangled states, such as a seven-qubit GHZ state in Bradley & Randall et al. PRX (2019). In such work, care had to be taken to mitigate the effects of crosstalk between various electron-nuclear spin gates. Other solid-state defects such as rare-earth ions enable electron-nuclear and electron-electron selective couplings via frequency selectivity (see https://arxiv.org/abs/2508.09122), omitting the need for a central electron spin to act as mediator between all spins and potentially enabling the formation of maximally entangled states comprising multiple electron spins.

By aggregating technical advances from several other papers, this work advances the toolkit for generating, verifying and estimating the fidelity of multiparticle entangled states in solid-state spin registers. In general, I believe this work is technically robust, the results are interesting and that the paper will be useful for a broader audience aiming to prepare and apply multipartite entangled states. However, the foundational ability to generate such a GHZ state with a central NV center electron spin has been present for some time. Given this, and the lack of substantial movement in this direction for several years, the paper would be strengthened if the utility of the faster, higher fidelity single gate scheme developed in this work was more fleshed out. If the authors can quantitatively substantiate the utility of this work and address the minor comments below, I would support publication in Nature Nanotechnology.

Specifically, the authors claim that the multipartite entangled states can be useful for Heisenberg sensitivity scaling. To use this local register for quantum enhanced sensing, how would it work, and what is the sensitivity enhancement relative to serial single gates? It's additionally unclear how far this scheme can be scaled in terms of number of qubits, and how a large quantum register of this type will be useful for any quantum computing applications. In principle, these gates could help advance quantum networking by enabling different quantum codes at individual network nodes, but some numbers would be useful here. How much better could a certain quantum code be, given this increase in multi-qubit gate fidelity and speed?

Small comments:
1. How many qubits can this scheme scale to? If the diamond is isotopically enriched with 13C, how many spins would be addressable? Additionally, what changes if trying to address multiple electron spins?
2. The use of k to index dynamical decoupling "orders" with different unit pulse time resonances in the main text was slightly opaque in regard to how exactly it was indexing these resonance times. Perhaps it is a matter of familiarity with the notation, but I believe it would make the actual DD sequences used here easier to understand if it were expanded upon at its introduction and not just in the methods.
3. Given the residual entanglement on the third nuclear spin in the L=2 GHZ states, is there any path forward to a protocol achieving isolated parallel entanglement on a subset of the nuclear spins addressable in the parallel order while maintaining superiority over sequential gates? That is, is there an N such that the residual entanglement is minimal while keeping gate times short and state fidelity high, and how does it scale with L? If not, how does that affect computational ability for the applications described in the paper, if at all?

Version 1:

Reviewer comments:

Reviewer #2

(Comments for the Author)
The authors have addressed my questions and I am happy to support publication in Nature Nano as-is.

Reviewer #3

(Comments for the Author)
I am a new reviewer assigned in this review round. In addition to evaluating the work itself, I was asked to assess whether the concerns raised by Reviewer #1 have been adequately addressed. I have read the reviewer comments from the previous round, the authors’ responses, and the revised manuscript. The main concerns raised by Reviewer #1 were the general applicability of the parallel entangling gate technique to future work in the field and its advancement beyond previous studies. These concerns have been addressed through revisions to the Introduction, the addition of a new section IV, and a brief discussion of applications to quantum error correction. In addition, I believe that all other technical concerns have been adequately addressed.

In the revised manuscript, the advantages of the parallel entangling gate and its general applicability are presented clearly. This work represents the first experimental demonstration of a parallel entangling gate between a central electron spin and weakly coupled multiple nuclear spins. The realization of a fast four-qubit entangling gate with moderately high fidelity is impressive. Therefore, I recommend publication of this work in Nature Nanotechnology after the authors address the following points.

In Table XI of the Supplementary Information, the parallel gate fidelity for q1 and q2 is lower than that of the sequential gate. On the other hand, QEC implemented with the parallel gate exhibits a much higher process fidelity than the sequential gate case, as shown in Fig. 26. Could the authors clarify why a higher QEC process fidelity is obtained despite the lower fidelity of the parallel gate? In addition, achieving very high-fidelity parallel entangling gates appears to be inherently challenging, as a parallel gate necessarily deviates slightly from the ideal conditional rotations. I agree that the parallel gate can improve gate fidelity and reduce gate time, but is a moderately high-fidelity gate of this kind truly useful for quantum error correction, which typically requires operation above a stringent error correction threshold?

If I understand correctly, in the MQC protocol, any unconditional rotations of spectator qubits caused by crosstalk do not affect the measured result. Are these rotations corrected, or are they simply ignored? Furthermore, do unconditional rotations of spectator qubits have any impact on the measured entangling gate fidelities?

In Fig. 3e, CX_pi/2 should be CR_psi2.

In Fig. 3c–f, the red (blue) curves correspond to the parallel (sequential) gate, respectively. However, the opposite color is used in Fig. 4d,e. In addition, the labels for the parallel and sequential gates in Fig. 4f,g appear to be incorrect.

Open Access This Peer Review File is licensed under a Creative Commons Attribution 4.0 International License, which

permits use, sharing, adaptation, distribution and reproduction in any medium or format, as long as you give appropriate credit to the original author(s) and the source, provide a link to the Creative Commons license, and indicate if changes were made.
In cases where reviewers are anonymous, credit should be given to 'Anonymous Referee' and the source.
The images or other third party material in this Peer Review File are included in the article's Creative Commons license, unless indicated otherwise in a credit line to the material. If material is not included in the article's Creative Commons license and your intended use is not permitted by statutory regulation or exceeds the permitted use, you will need to obtain permission directly from the copyright holder.
To view a copy of this license, visit https://creativecommons.org/licenses/by/4.0/

Revisions and Response to Reviewer Comments

In the following, Reviewers' comments are formatted in italic font. Responses to each comment and actions taken are formatted in normal font, with revisions to the text formatted in blue font.

Major revisions to address comments by both reviewers

Both reviewers were interested to understand more about the generality of the multipartite entanglement method, the methods' applications to quantum error correction, and the ability to control subsets of the nuclear register qubits. Our revised manuscript includes new data, figures, and discussion to address these questions specifically, as follows.

Major action 1: New "Generality and extensions" sections

We added a new section to the main text entitled – "IV. Generality and extensions," along with a new associated Figure 5, as follows:

IV. Generality and extensions

To contextualize these results, we simulated 500 random, weakly-coupled NV–nuclear qubit registers to evaluate their capacity for parallel entanglement. We excluded configurations with one or more strongly-coupled qubits, although such mixed systems could potentially be addressed as well. For each remaining configuration, we determined which combinations of nuclear qubits could be entangled in parallel, and we calculated the corresponding gate parameters for both parallel and sequential operations. The results, summarized in Fig. 5, illustrate that L = 2 and L = 3 parallel entanglement gates are available in the majority of configurations (Fig. 5a). In some cases, entangled states of four or even five nuclear qubits can be created. Furthermore, the registers generally support parallel entanglement of multiple unique qubit combinations (Fig. 5b). The parallel gate duration remains approximately constant as L increases, whereas the sequential gates steadily increase (Fig. 5c). Thus, the speedup of the parallel approach becomes more pronounced with increasing L (Fig. 5d). Further simulation details are provided in Methods Sec. VIG, with additional simulations in SI Sec. VI.

The experimental results reported here fall within the simulated distributions. In future work, registers can reasonably be identified that enable L = 4 or L = 5 parallel entangling gates. The L = 5 case is of particular importance for fault tolerant operation [11]; see SI Sec. XI for further discussion and simulations of quantum error correction.

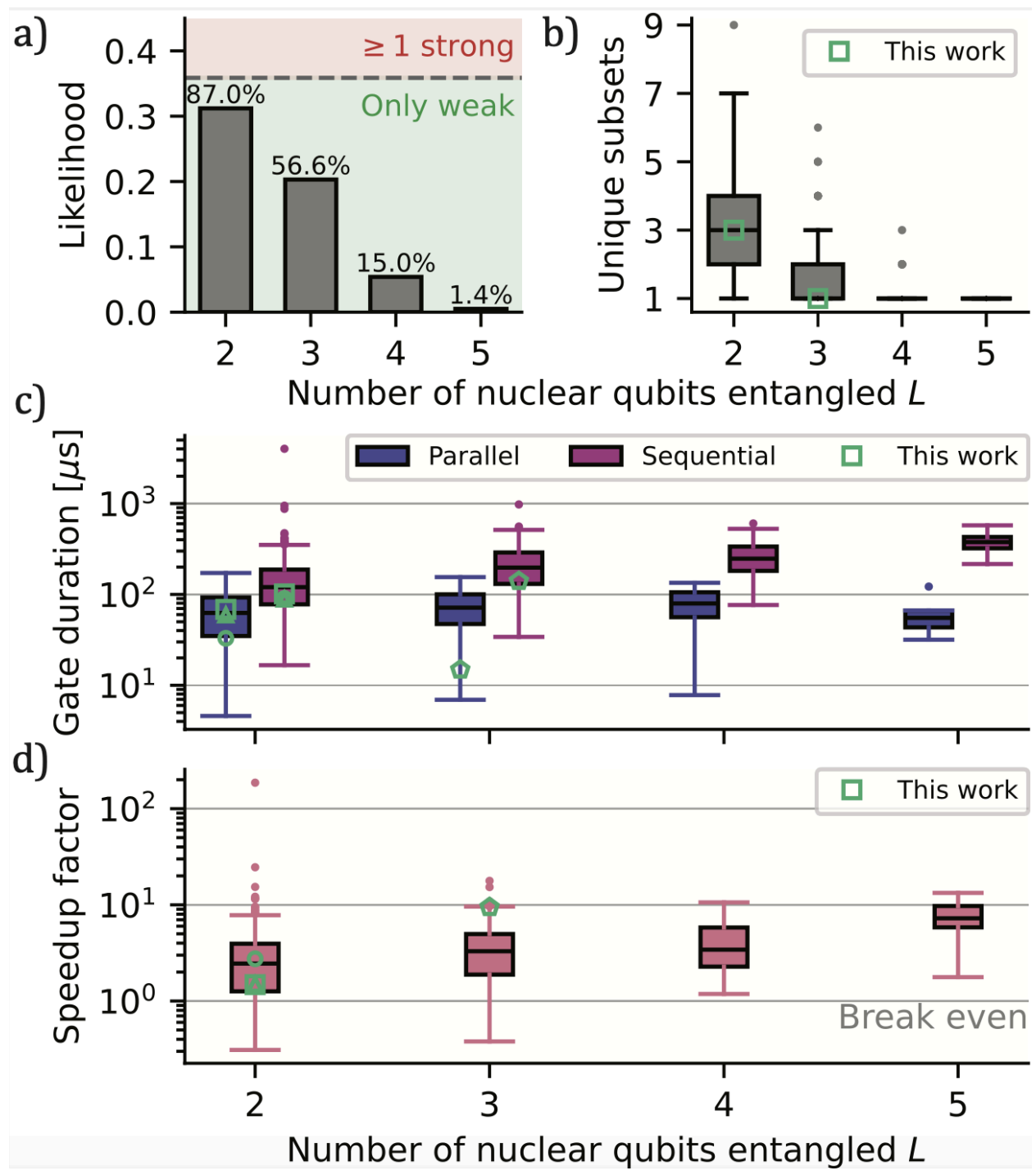

Fig. 5 Generality of parallel entanglement in NV-nuclear registers (a) Likelihood of a register supporting parallel entanglement. In this analysis, we exclude registers that contain at least one strongly coupled nuclear qubit (red region, corresponding to 64% of configurations). Percentages listed above each bar correspond to the likelihood of weakly-coupled registers (green region) supporting parallel entanglement with at least one set of L nuclear qubits. On average, the weakly-coupled registers contain 5.7 (2.5) individually-addressable nuclear qubits in total. (b) Number of unique subsets of L nuclear qubits in registers hosting parallel entanglement. The middle black line represents the median, the box spans the interquartile range (IQR), the whiskers extend to 1.5× the IQR, and points indicate outliers. (c) Parallel and k = 2 sequential entangling gate durations. (d) Parallel entanglement speedup factor.

Major action 2: New "Applications to quantum error correction" section

We added a new section to the Supplementary information discussing "Applications to quantum error correction." This section describes how the parallel three-qubit (L=2) gates can replace sequences of two-qubit gates to realize a three-qubit bit-flip code. We further discuss why the parallel entangling gates are promising for use in more complex, fault tolerant codes that have only recently been achieved. See Major action 3 for details on the realization of L=2 parallel entangling gates. The new section consists of the following text and figures:

XI. Applications to quantum error correction

Following the quantum circuits of Takou et al. [4] and the experimental techniques of Taminiau et al. [6], we simulate how the parallel entangling gates used in this work could improve quantum error correction protocols. In this example, a three-qubit bit-flip code is simulated that can detect and correct any single qubit bit-flip errors by majority voting.

The protocol is simulated two ways — one using a sequence of two-qubit gates in the encoding and decoding steps (Fig. 26a), and the other using a single parallel entangling gate (Fig. 26b). The correction is implemented using a doubly controlled not (Toffoli) gate, which is idealized in the following simulations to accentuate only the differences in the entangling gates used during encoding and decoding. The Toffoli gate can be implemented with a sequence of single and two-qubit DD gates, but is therefore subject to their crosstalk. Similarly, register initialization is idealized in all cases. Single qubit errors are induced on the electron as a variable X_θ rotation. The correction is turned off by flipping one of the nuclear qubits at the start of the circuit (keeping it in $|0\rangle$ at the start). In the parallel case, additional local Y_π gates are needed on the nuclear qubits to compensate for the complicated geometry of the parallel gate [4], also idealized in the following simulation. The electron is prepared in 6 initial states $|\psi\rangle = |\pm X\rangle$, $|\pm Y\rangle$, $|\pm Z\rangle$, from which the process fidelity as a function of error angle is calculated according to [6] (Fig. 26c). Notably, the parallel gate maintains approximately double the process fidelity than the sequential entangling gates over all error angles — which is directly related to its higher fidelity. With no bit-flip error on the electron, the parallel gate still does not reach unit process fidelity with idealized local nuclear gates due to residual entanglement generated by the next strongest nuclear qubit. Parallel gates with lower amounts of residual entanglement will behave even closer to the idealized blue curve of Fig. 26c. Improvements to such QEC circuits with the parallel gate can immediately benefit recent studies of hybrid entanglement between the electron, multiple ^{13}C nuclear qubits, and photons at low temperatures for error corrected quantum networking applications [14].

While three-qubit bit (and phase) flip codes are useful for single qubit errors, they fail to correct multiple errors and are overall not fault-tolerant. With additional nuclear qubits, minimal fault-tolerant protocols can be achieved with parallel entangling gates using a similar approach as in Fig. 26b. The first fault-tolerant operation of a NV-nuclear qubit register was demonstrated using L = 5 weakly coupled ^{13}C nuclear qubits, as well as the ^{14}N qubit as a flag qubit [15]. The encoding and decoding steps to form the logical qubit required a sequence of two-qubit gates, dominating the circuit depth. Based on the simulations of Sec. VI, L = 5 parallel entangling gates with properties similar to the ones demonstrated in this work are possible in a small, but reasonable, fraction of NV centers. Searching for such an NV center could drastically reduce circuit durations and errors, potentially enabling fault tolerance at room temperature for the first time.

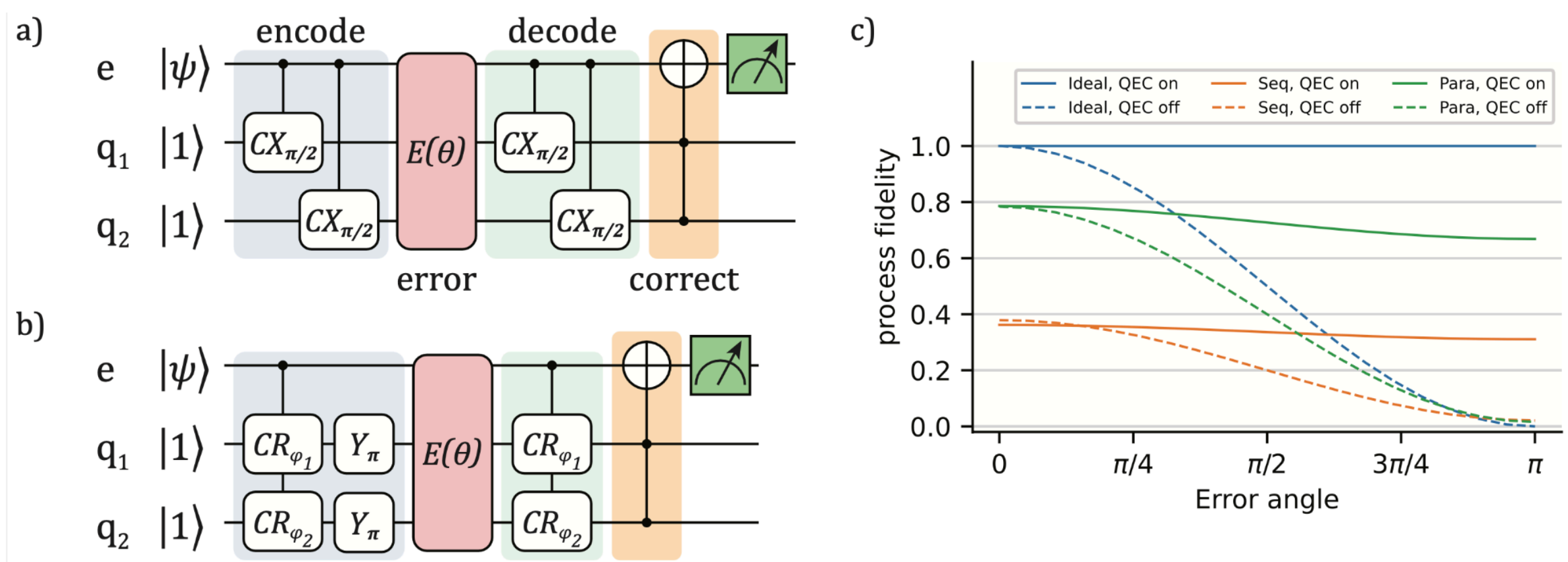

Supplemental Fig. 25 Three-qubit bit-flip (a) bit-flip code using sequences of two-qubit gates. (b) bit-flip code using parallel three-qubit gate. Local nuclear Y_π gates are needed to compensate for entangling gate geometry. (c) Process fidelity of correcting bit-flip error on the electron qubit. ”QEC on” refers to the initial nuclear state |11⟩, while ”QEC off” refers to the initial nuclear state |01⟩ (equivalently, |10⟩). Since the code cannot correct two bit-flips, the flipped nuclear qubit deactivates the code.

<u>Major action 3: New data and discussion for L=2 parallel entangling gates</u>

To address the reviewers’ questions regarding the design of entangling gates to target subsets of the register qubits, as desired for quantum error correction protocols, we added new experimental results and associated discussion showing how this can be achieved. Whereas the MQC data for L=2 originally included in Fig. 3 exhibited features due to conditional crosstalk with the remaining spectator qubit, we found that the crosstalk could be eliminated by optimizing the choice of *N*. In this way, we were able to design and implement three-qubit entangling gates for all possible subsets of nuclear spins. An example of the new parallel L=2 MQC data was added to Fig. 3, replacing the previous data that contained conditional crosstalk. The conditional crosstalk data was moved to the Supplementary Materials since it is still valuable in highlighting how residual entanglement can cause observable effects with the MQC method. The text discussing the L=2 MQC data was updated as follows:

> Starting with L = 2 (two nuclear qubits), we can design a parallel three-qubit entangling gate with any subset of (q1, q2, q3); Fig. 3c shows the example of e, q1 , and q2 . Since any residual entanglement with the spectator nuclear qubit will lower the entangling gate fidelity, the L = 2 gate parameters must be selected such that crosstalk on the spectator qubit is unconditional (see SI Sec. V for details). The MQC method is also sensitive to such effects; residual entanglement manifests as multi-frequency beating in the signal (see SI Sec. IX for theoretical analysis and exemplary data). The single-frequency oscillation observed in Fig. 3c, with L = 2, confirms that the gate creates a tripartite entangled state as desired. The sequential approach (with optimized crosstalk corrections) also generates a tripartite state with the expected MQC signal (Fig. 3d); however, the sequential gate duration is almost 50% longer than the parallel gate.

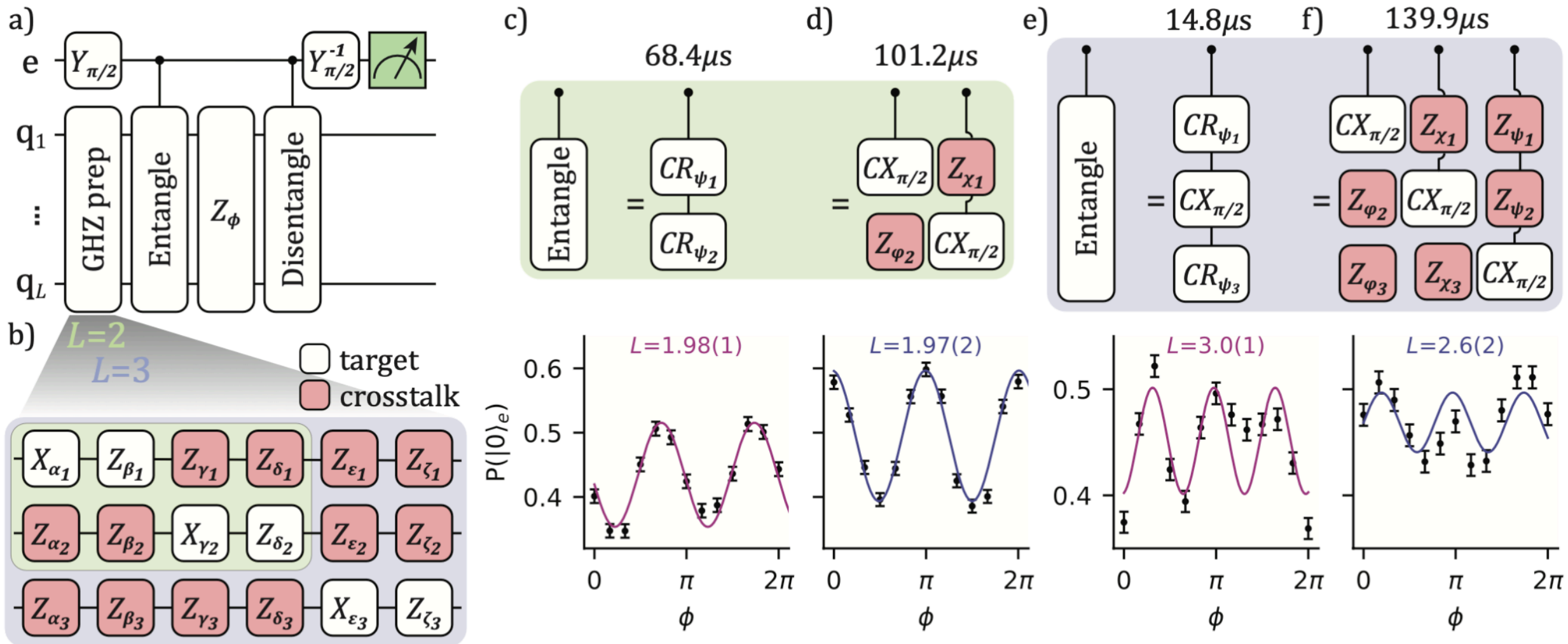

Fig. 3 Generation and verification of multipartite GHZ states Block-diagram quantum circuit for entanglement verification using multiple nuclear qubits. Qubits are initialized sequentially as shown in Fig. 2b. (b) Local gates used to prepare for GHZ state generation for L = 2 or 3 nuclear qubits (green and blue shaded regions, respectively). Red shaded gates represent unwanted crosstalk effects, which manifest as Z-axis rotations on each nuclear qubit in the register that is not targeted with a white gate. The angles of the local gates (associated with the unit pulse repeats) were determined using numerical optimization, with the gate axes (associated with unit pulse times) remaining fixed as shown. (c,d) Results of three-qubit (L = 2) MQC verification experiments using nuclear qubits q1 and q2 (data points in lower panels) corresponding to the parallel and sequential entangling gates shown in the upper panel. The corresponding disentangling gates in (a) are identical. Red (parallel) and blue (sequential) curves are sinusoidal fits to measure the number of entangled nuclear qubits L. See the SI, Sec. IX for results corresponding to all other combinations of qubits. (e,f) Results of four-qubit (L = 3) MQC verification experiments. Error bars on points denote photon shot noise, and uncertainties on L are 68% confidence intervals from from fits to equation (2).

Likewise, Fig. 4 was expanded to include entangling gate fidelity measurements for all possible subsets of nuclear qubits using both the parallel and sequential entangling approaches. The discussion of the results was revised as follows:

> We observe an average (standard deviation) bipartite gate fidelity of G_2^{avg} = 0.96(1). The three-qubit gate fidelities vary significantly: the parallel gate fidelities are anti-correlated with how many electron operations are needed (Fig. 4g), whereas the sequential gates are further limited by their intrinsic crosstalk. Nonetheless, the average fidelity for the parallel gates, G_3^{para} = 0.83(9), exceeds that of the sequential approach, G_3^{seq} = 0.78(8), and the parallel gates are ~2 times faster. For M = 4, the advantages of the parallel approach become dramatically apparent. The four-qubit parallel gate is the fastest gate overall, having a similar number of pulses as the two-qubit gates but a shorter t; this contributes to its high fidelity of G_4^{para} = 0.92(4), despite the large number of qubits

involved. The sequential gate, on the other hand, exhibits a low fidelity of G_4^{seq} = 0.69(3), due to the combined impacts of crosstalk, its longer duration, and a large number of electron pulses.

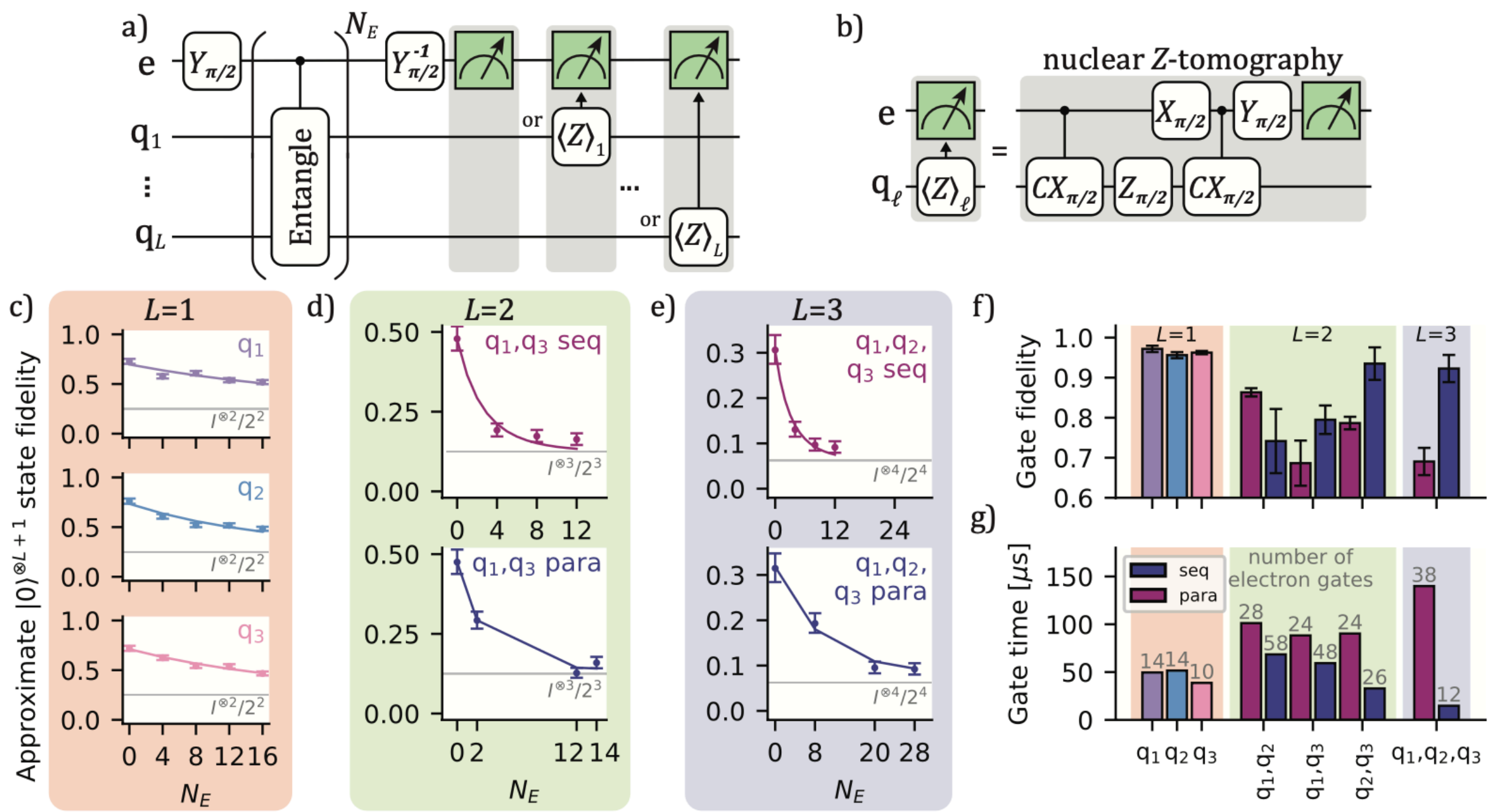

Fig. 4 Entangling-gate fidelities (a) Quantum circuit to efficiently measure entangling-gate fidelity. After repeating the entangling gate N_E times, the Z projection of either the electron or an individual nuclear qubit is measured, and the combined results are used to approximate the $|0\rangle^{\otimes M}$ state fidelity. (b) Sub-circuit to measure nuclear-qubit Z projections. (c) Experimental results (data points) for bipartite entangling gates associated with each nuclear qubit. Solid curves are fits using a noisy quantum channel model to extract the entangling-gate fidelity. (d,e) Experimental results (data points) for three-way and four-way entanglement generated either sequentially (top panel) or using a parallel gate (bottom panel). Repeating the parallel gate does not generally return exactly $|0\rangle^{\otimes M}$ due to its more complicated geometry; values of N_E were chosen to maximize the overlap with $|0\rangle^{\otimes M}$. Fits to the data (solid curves) account for this effect along with gate errors and are used determine the fidelity of each gate. Error bars on points in (c-e) are propagated from each measurement's photon shot noise. (f) Entangling-gate fidelities determined from fits to the data in (c-e). Error bars denote 68% confidence intervals from fits to the noisy quantum channel model. (g) Entangling-gate durations. The number of electron pulses (equivalent to 2N) are printed above each bar.

The design and calibration of the L=2 parallel gates was made possible through use of the non-unitary entangling power, rather than the unitary entangling power discussed in the original paper. The details of this metric pertaining to the calibration of these gates was added to the "Entanglement metrics" methods section of the main text:

We utilize the non-unitary entangling power to account for the impact of residual entanglement generated with non-targeted nuclear spins [41]. This entanglement metric

is derived using the partial trace quantum channel E over non-targeted nuclear qubits. The set of all nuclear qubits is partitioned into a subset that is targeted (size L) and the rest that are not targeted (size $L_{total} - L$). A simple approximate form for this entanglement metric is given by

$$\varepsilon_{p,M}(\mathcal{E}) = \frac{\varepsilon_{p,M}(U_{DD})}{2}\left(1 + \prod_{\substack{\ell\in\text{not}\\ \text{targeted}}}^{L_{\text{total}}-L} G_1^{(\ell)}\right). \qquad (11)$$

The non-unitary entangling power is bounded above by the unitary entangling power; $\varepsilon_{p,M}(E) \leq \varepsilon_{p,M}(U_{DD})$, with equality holding when no residual entanglement is generated ($G^{(\ell)}_1 = 1$ for all non-targeted nuclear qubits) [41]. Therefore, any residual entanglement generated leads to a Makhlin invariant less than 1, lowering this entanglement metric. This metric is essential when designing parallel entangling gates with subsets of known nuclear qubits, e.g., in the case of L = 2 parallel entangling gates in this work. Additional details regarding how these metrics were used to calibrate each parallel entangling gate can be found in SI, Sec. V.

Additionally, a substantial portion of Supplementary Information Sec. V.C surrounding parallel gate calibration was updated, including new simulation plots taking residual entanglement into account in Supplemental Figure 8.

Reviewer #1 (Comments for the Author):

Summary:

The paper by Minnella et al. reports on the experimental demonstration of a single parallel dynamical-decoupling (DD) gate that generates a four-qubit GHZ state (the NV electron plus three proximal 13-C nuclei) at room temperature. In the more well-known method of generating GHZ states, on a previous device with similar hyperfine coupling values, such an entangling sequence was composed of sequential DD-sequences for each nuclear spin. In this work, the authors introduced a faster method of entanglement by leveraging the crosstalk from the DD gates due to similar hyperfine coupling values.

Using this multi-qubit gate they generated a 4-qubit GHZ state approximately 10 times faster than if they were to use the sequence of two-qubit gates, reducing their entangling gate operation time from 139.9 µs to 14.8 µs and leading to an improvement in gate fidelity from 0.69(3) to 0.96(1). While existing limitations remain dominated by room-temperature SPAM errors and errors from the local hyperfine landscape, they conclude that such a multi-qubit DD gate is well-positioned for the scalable generation and control of entanglement in solid-state registers for both error correction and entanglement-enhanced quantum sensing.

Response: We thank the reviewer for their thoughtful and thorough evaluation of our paper.

Three key questions stand out:

1. How is this technique applicable to other qubit platforms, and indeed to other devices in the same system, where it is not so easy to achieve similar hyperfine couplings values?

Response: This technique is broadly applicable to quantum registers comprised of a central spin with weakly coupled qubits, which is ubiquitous among naturally occurring central-spin systems with broad generality to other solid-state qubit systems. The distinction between weak coupling (i.e., hyperfine coupling frequencies smaller than 1/T2*) and strong coupling (with hyperfine coupling frequencies larger than 1/T2*) was not emphasized in the original paper, and this has been addressed in our revisions. In systems with strongly coupled qubits, such as those the reviewer mentions later, entangling gates are achieved via direct driving of transitions conditional on the qubit states, rather than via dynamical decoupling. However, quantum register systems with large numbers of individually addressable, strongly coupled spins are exceedingly rare in nature, so they must be identified through extensive screening or engineered near the atomic scale. In such systems, furthermore, large differences between hyperfine couplings are generally advantageous to reduce gate crosstalk, and this quickly presents scaling limitations due to the required spectral distinguishability.

For weakly-coupled registers, in contrast, the flexibility of DD gate design means that the approach presented in this work is **likely applicable to any weakly-coupled NV-based register, and moreover to any analogous central-spin system**. To quantitatively substantiate this claim, a new section “Generality and extensions” has been added to the main text that simulates over 500 other NV-nuclear quantum registers and investigates their capacity to host parallel entanglement. Likewise a new section by the same title was added to the Methods, with an extended discussion added to the Supplementary Information Sec. VI.

Action taken: Please refer to Major Action 1, described at the opening of this document, for revisions in response to this question about generality. To further emphasize the type of systems this parallel entanglement framework applies to, the following updates have been made in the introduction. When discussing parallel entangling gates in the broader context, we added:

> For solid-state registers operating in the strong-coupling regime (i.e., with hyperfine coupling frequencies larger than the electron spin's inhomogeneous linewidth), multi-qubit entangling gates can be achieved using frequency-selective conditional electron pulses [16–19]. However, strongly-coupled registers are rare in nature, so they either need to be engineered [17–19] or identified through screening [16]. Their scalability is limited by the need for strong, widely spaced couplings for individual addressability. Weakly-coupled registers typically offer larger register sizes that enable more robust error correction and networking capabilities [4, 10, 11], but the sequential application of DD sequences leads to impractical gate times and errors for multi-qubit control.

Furthermore, to distinguish the weak and strong coupling regimes, we revised the following sentences in the last paragraph of the introduction:

> We use the entanglement metric framework developed by Takou et al., [40, 41] to design DD sequences that address multiple weakly coupled ^{13}C nuclear qubits surrounding an NV center (Fig. 1a)

As well as

> We experimentally generate four-qubit Greenberger–Horne–Zeilinger (GHZ) entangled states that include the electron and three weakly coupled ^{13}C nuclear qubits…

2. For scalability other limitations still dominate such as SPAM errors and local hyperfine variations, can the authors address this in their argument for scalability

Response: We agree that this is an important point in the context of scalability. The question regarding hyperfine variations was addressed previously, with the conclusion that the approach is broadly scalable across NV-center and similar qubit systems. Regarding SPAM errors, this is addressed in the conclusions section as follows:

> The entangled-state fidelities are primarily limited by SPAM errors associated with the NV center's room-temperature properties. These can be solved by working at cryogenic temperatures [46]. Alternatively, the room-temperature SPAM errors can be reduced using repetitive readout at high magnetic fields [47], spin-to-charge conversion [48], or dynamical nuclear polarization methods [49].

3. They have not demonstrated or discussed in detail how this will be used for quantum error correction and this should be addressed.

Response: We agree that quantitatively showing how the new parallel entangling gates can be used for quantum error correction should be addressed. To that end, we have added a new section to the Supplemental Information, “Applications to quantum error correction”.

Action Taken: Please see <u>Major action 2</u> at the beginning of this document.

Originality and significance

The realization of multi-qubit gates in solid-state spin registers is not in itself new. For example, multi-qubit entangling gates have been previously demonstrated in nitrogen vacancies (see Waldherr et al, Nature 506, 204-207 (2014)) and later in silicon with fidelities up to 99.9% (see H. Edlbauer et al., arXiv:2506.03567v1 and I. Thorvaldson et al., Nature Nanotechnology 20, 472-477 (2025)). Note that, these previous results leverage the geometric phase of the electron instead of dynamical decoupling (DD) gate sequences.

Response: Thank you for highlighting this important body of work. While relevant to our work, all of these papers utilize strongly coupled central spin registers — a different regime of physics than our work that rarely occurs naturally and is difficult to scale. A strongly-coupled register requires the existence of multiple nuclei with strong and distinct hyperfine couplings – meaning they are only a few lattice sites away from the electron – within an otherwise nuclear-spin-free environment. Such atomic arrangements are statistical outliers; Waldherr et al, 2014 describe in their Methods section that it was necessary to screen over 3,300 NV centers in order to identify the one they ultimately used, and that system only included two ^{13}C nuclear qubits. On the other hand, weakly coupled nuclear registers occur commonly in nature, and they offer a clear path to achieve larger register sizes. One of the trade-offs is that more complicated nuclear qubit control schemes (such as DD) are required since the nuclear qubit transitions are, by definition, no longer resolvable from the electron spin transition linewidth. Such gates naturally take longer to implement, given the weaker interaction strength, and are susceptible to crosstalk. Our approach of parallelizing the entangling gates directly addresses both of these tradeoffs, advancing the scalability of quantum registers in this practically relevant regime.

Action taken: To summarize the works mentioned and the differences in physical regimes, the introduction when discussing parallel entanglement in a broader context was revised and expanded (see changes previously described in response to reviewer question 1).

Nuclear control utilising dynamically decoupling pulses of the electron has been studied in many previous works in nitrogen vacancy systems (such as T. H. Taminiau et al, Nature Nanotechnology 9, 171-176 (2014)). While earlier techniques primarily focused on sequential operation of the nuclear spins, recent theoretical work by Takou et al (Phys. Rev. X 13, 011004 (2023) & Quantum 8, 1304 (2024)) has shown that it is possible to perform fully entangling operations over multiple nuclei, provided they are similar enough in resonance frequency. The result shown by Minnella et al represents, to our knowledge, the first experimental demonstration of such proposed pulses, characterising the fidelity of these operations on a physical device. Due to the slower operations needed to individually address nuclei with similar

hyperfine coupling values, the result presented does indeed show a 10x speedup (from 139.9us to 14.8us) for simultaneous GHZ entanglement compared to sequential entanglement. However, it is important to note that other devices, where the nuclear spins are more addressable (see Edlbauer et al, Thorvaldson et al, Waldherr et al, Taminiau et al above) observe entanglement times of ~10-30us, meaning that the techniques demonstrated by Minnella et al seem to be only specific to devices where the ability to address individual nuclei is challenging. It would be good for the authors to comment on this.

Response: We appreciate the reviewer's comments regarding the novelty of this approach and the practical importance of faster gates. We are happy to address the question of pulse times, which further relates to the difference between strongly and weakly-coupled registers.

Regarding the specific gate durations, it is again useful to separate the two physical regimes in the works mentioned above. Works such as Edlbauer et al, Thorvaldson et al, and Waldherr et al all utilize strongly coupled qubits, with interaction strengths on the order of 1-100MHz. One can consider the inverse of the interaction strength (frequency) as an approximate fundamental lower limit on the entangling gate duration. This would suggest gates as fast as 10ns to 1us are possible. In practice, to implement multi-qubit gates in this regime, the driving strength cannot be larger than the minimum difference in interaction strength, otherwise resonances will spectrally overlap and the operation will no longer be conditional on the given state. In turn, this leads to entangling gate durations much slower than the fundamental limit, with durations in the range that the reviewer quotes.

On the other hand, works such as ours and Taminiau et al, 2014 apply to the weakly coupled regime, with interaction strengths on the order of 10-100kHz. The lower interaction strengths result in fundamental gate limits of 10 to 100us. Historically, the approach to entangling gates using DD has had the same limitations as strongly coupled registers, where higher orders ($k \geq 2$) are needed to resolve individual nuclei based on differences in hyperfine coupling. The hallmark of the parallel entangling gate approach (available at $k=1$) is in leveraging qubits' spectral overlap to achieve the desired operation and eliminate the previous speed limits due to coupling differences. In our experiments, two of the nuclei are distinguished by only ~10 kHz and yet gates involving those nuclei are substantially faster than 100 us. Additionally, the ability for a register to host parallel entanglement does not imply that nuclei are intrinsically challenging to address as the reviewer suggests — we do so with high fidelity using second order DD two-qubit gates.

As an example, Taminiau et al, 2014 created entangled states through the sequential application of individual, spectrally resolved, DD gates. There, higher-order ($k=3$ and 4) resonances were used to provide higher spectral resolution over longer times. As a result, Taminiau et al, 2014 required ~150-200us to achieve a two-qubit gate, far from the fundamental limit and also far from ~10-30us that the reviewer noted. To highlight this distinction, we used the hyperfine couplings of Taminiau et al, 2014 to design parallel entanglement gates and compared them to the corresponding gate times for $k=2$ and $k=3$ order sequential gates. This example further illustrates the technique's generality, since we were able to obtain a complete set of entangling gates (one four-qubit gate and all three possible three-qubit gates) for this hyperfine configuration. Notably, the four-qubit gate is approximately 14 times faster than

sequences of third order two-qubit gates and operates near the fundamental speed limit. The results are summarized in the table below.

Taminiau 2014 nuclear qubits	k=1 parallel duration [μs]	k=2 sequential duration [μs]	k=2 speedup	k=3 sequential duration [μs]	k=3 speedup
q1,q2	64	135	2.1x	271	4.2x
q1,q3	45	206	4.6x	405	9.0x
q2,q3	84	175	2.1x	306	3.6x
q1,q2,q3	36	258	7.2x	491	13.7x

Rebuttal Table 1. Taminiau et al, 2014 hyperfine couplings parallel entanglement capacity and durations. Only two-qubit gates for q1 and q2 were used in Taminiau et al, 2014. The durations were 170 and 140us and at k=3 and 4 order resonances, respectively. Note, this table is not included in the revisions and is merely an example to highlight the broad applications of the parallel entangling methodology.

Action taken: The revised introduction now highlights the weak-coupling physical regime in which the parallel entangling method is applicable. The new Figure 5 and associated discussion (see Major action 1) shows the expected durations and speed ups for random distribution of nuclear configurations, verifying that our results lie within the expected distributions.

Overall, we believe that the results shown by Minnella et al are novel, in that they are the first experimental demonstration of simultaneous entanglement in solid state systems using dynamical decoupling techniques. This technique provides an experimentally verified alternative to the existing dynamical-decoupling control techniques which are well-established in the solid-state qubit field. We believe that in some unique cases, where hyperfine couplings are similar, this may result in both faster device operation and higher operational fidelities. It would be good for the authors to comment on how wide-spread this technique would be available.

Response: We thank the reviewer for the positive feedback pertaining to the novelty of our work. We trust that, with the new section “Generality and extensions” (see Major action 1) the reviewer will agree that this approach is not limited to “unique cases”, but is applicable to a broad class of quantum registers.

We can see that the results were obtained at room temperature, but this technique should be applicable to devices operated at cryogenic temperatures.

Response: This technique is certainly applicable at cryogenic temperatures. In addition to extended electron-spin lifetimes at low temperatures, nuclear SPAM errors can be greatly

reduced, and correlators between the electron and nuclear qubits can be directly measured via high-fidelity electron-spin readout.

Regarding the significance of these results, the main concern therefore is to the extent this method is applicable to future devices which can have different hyperfine coupling strengths and number of nearby nuclear spins. It would be helpful if the authors can quantify the influence of the variation in hyperfine coupling on speed and simulated gate fidelity.

Response: We hope that the reviewer is satisfied with our responses above regarding the generality and future applicability of this technique (see especially the revisions discussed in Major action 1). Regarding gate speed and fidelity, the new Figure 5 quantitatively predicts the gate speedup, whereas gate infidelity distributions due to residual entanglement are shown in the associated Supplementary Information section VI.

In addition, the authors mentioned the benefit of such an entangling gate for quantum error correction (QEC) schemes. However, the authors did not mention which QEC method would benefit from it since traditional surface code would require multi-qubit gate for a subset of data qubits at the time, instead of operating on all qubits at once.

Response: We appreciate this question and are happy to elaborate. Given the central-spin topology of interactions in electron-nuclear qubit registers, traditional surface codes are probably not the best path to achieve fault tolerance. The nuclear register is more analogous to ion traps and other systems that utilize clusters of qubits with all-to-all connectivity, rather than qubit arrays. For example, protocols such as that demonstrated in Abobieh et al, *Nature* (2022), that utilize the electron and ^{14}N as a flag qubits to detect errors are better suited for the system. The new Supplementary Materials section "Applications to quantum error correction" (see Major action 2) discusses this topic, supported by simulations showing how the parallel gate improves the execution of three-qubit bit-flip correction codes and how fault tolerance algorithms can be achieved using the new parallel gates.

Nonetheless, the reviewer raises an important point about realizing entangling gates on subsets of nuclear qubits. While we had focused on the L=3 parallel gate in our original submission, we returned to consider L=2 gates using the framework of non-unitary entangling power discussed by Takou et al, 2024. We found that we could achieve near maximal entanglement between any two of the three register qubits by increasing N. We further used the optimized L=2 parallel entangling gates to demonstrate MQC and quantify the entangling gate fidelity.

Action taken: See Major action 2 for QEC related changes and Major action 3 for L=2 parallel entangling gate MQC data and fidelities.

Data and methodology

Overall, the data and methodology appear solid and are generally aligned with established techniques in NV-center quantum control. The authors rely heavily on simulation to identify optimal DD parameters (such as t and N for multi-qubit entangling gate), including the

compensation phases for local gates. However, some methodological choices and omissions require clarification, and several figures would benefit from additional contextual information or consistency in presentation.

Figure 2
The Multiple Quantum Coherence (MQC) method is correctly cited (Refs. 42–43). The data are fit with Eq. 2 as expected, although it may be helpful for the reader to mentioned it in the figure caption.

Response: We agree it would be helpful to mention this explicitly in the figure caption.

Action taken: The following text has been added to the end of the caption describing Fig 2d.

> Error bars on points denote photon shot noise, and uncertainties on L are 68% confidence intervals from from fits to equation (2) with a variable amplitude, midpoint and phase.

Figure 3
The parallel entanglement method is correctly attributed to Refs. 40–41. The data is fitted with theory and additional data sets are shown in the supplementary information, although the fitted phase compensation parameters are not shown.

Response: We agree that showing the compensation parameters would be useful for completeness.

Action taken: Tables in the Supplementary Information Sec. IX.B have been added containing all optimization results for phase compensation.

An important point to raise is the key results shown in fig3c. The oscillation may have a secondary frequency (similar to fig 3e). What does this plot look like if a second frequency is included?

Response: First, please note that we have reordered the figures such that the L=3 MQC data now appears in Fig. 3(e), and the L=2 data the reviewer references from fig 3e has now been replaced with new data (now Fig. 3(c)) that does not show a secondary oscillation. Nonetheless, we are happy to address the reviewer's question.

Indeed there appears to be a small amount of beating present in the L=3 parallel MQC signal. The data can be fit using multiple modes, as shown in the Figure below, where each oscillation was fixed in frequency at integer (1,2,3,4) modes with variable amplitudes, phase shifts and a common vertical shift. The L=3 mode dominates the fit with the largest amplitude, as expected for entanglement with L=3 nuclear qubits. Notably, an exact simulation of the circuit (Supplementary Information Sec. IX.B Figure 17) also shows a reduction in contrast around $\phi \sim \pi$ to 1.5π. Hence, we believe these smaller features mainly reflect subtle details of the entangling gate geometry and nonidealities of the disentangling step. The important conclusion

to be drawn from the MQC experiment is the dominant contribution of a mode at L=3, which we believe is best conveyed through the single-frequency fit included in the manuscript.

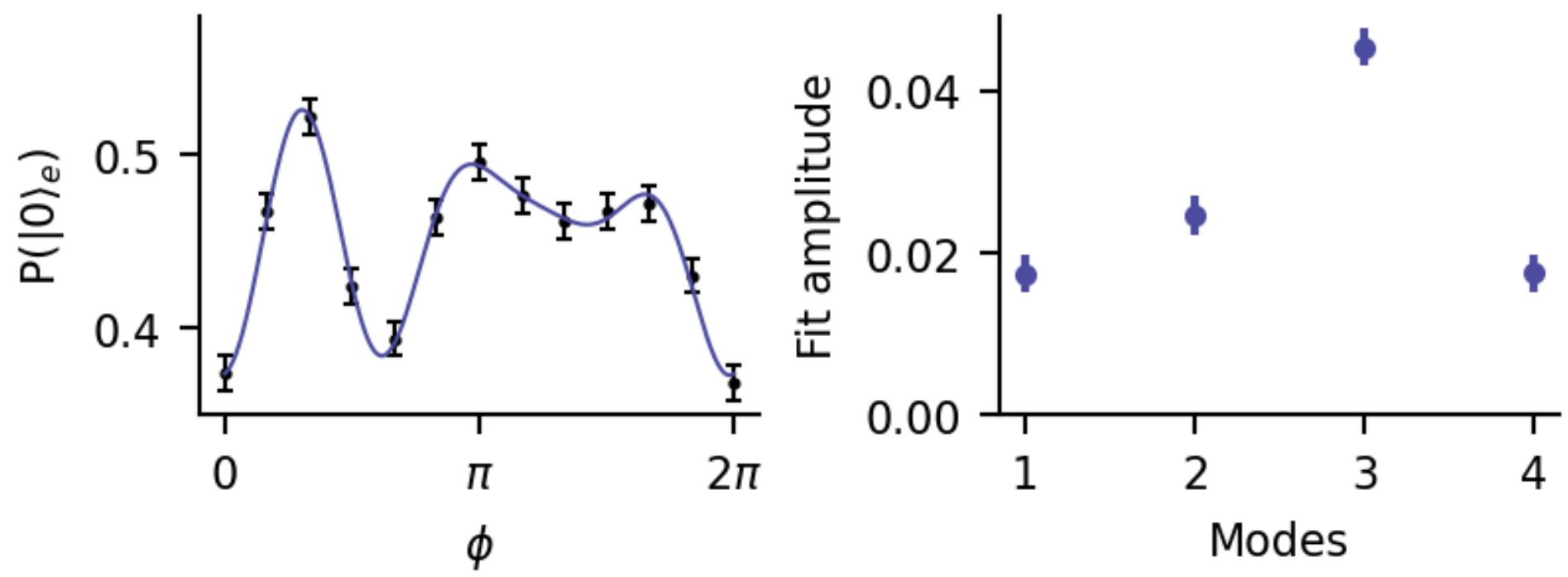

Rebuttal Fig. 1 Multi-tone fit to L=3 parallel MQC data. Error bars on fit amplitudes denote 68% confidence intervals.

In addition, the uncertainty in L for fig 3c is smaller than fig 3d, even though the latter seems to have a cleaner oscillation pattern. If the authors can comment on these points, it will be strengthening their conclusion.

Response: The unrounded uncertainties are 0.14 and 0.17 for the L=3 parallel and sequential fits. Rounding to the appropriate precision exaggerates the difference between them.

Figure 4
The methodology adapted from Evered et al. (Ref. 36) seems appropriate, and separating SPAM from gate error is critical given the room-temperature constraints. The use of a quantum depolarizing channel to fit fidelity decay is reasonable. The choice of N_E for parallel gates is shown in the supplementary information. One concerning point is that the authors assume there is no correlated errors in the final state, and therefore they can apply their model to extract the gate fidelity. However, it will strengthen their conclusion if they quantify the extent of the correlated errors.

Response: We appreciate this concern and can elaborate on this point further. For bipartite entanglement, the difference between the exact state fidelity and the approximate state fidelity is exactly given by the covariance of each Z observable. When the qubits have some degree of entanglement and thus correlation, this covariance is nonzero and is the difference in our approximation from the exact value. More generally, when considering multipartite entanglement, the difference is based on each pairwise covariance as well as the (L+1)th order cumulant. All of these points are established mathematically in Section X.A of the Supplementary Information.

Nonetheless, the exact difference in the state fidelity expressions can be taken using simulations with access to all correlations of qubits in each case and highlights the net effect of the approximation. Notably, the approximate state fidelity is always less than the exact fidelity, approximately growing larger as N_E increases. Thus, the state fidelities calculated experimentally decrease faster as a function of N_E than the exact values, and therefore the

quantum channel fit to the data produces a lower entangling gate fidelity. While we do not have a mathematical proof of this fact, this observation suggests that the experimental entangling gate fidelities we obtain actually *underestimate* the exact values.

Action taken: To quantify the extent that correlated errors affect the state fidelity, a new figure and the following text was added to the Supplementary Information in Sec. X.D:

> To give some insight into the nature of using approximate state fidelities to calculate the gate fidelities, the state fidelities created by the parallel entangling gates were calculated using both the exact trace fidelity as in equation (68) and the approximate fidelity in equation (70). The difference in fidelities depends on the extent to which correlations exist within the system and subsystems (see equations (73) and (75) for exact expressions). Fig. 25 reveals that the approximate state fidelity is always less than the exact, with the difference approximately growing larger as a function of N_E. A similar behavior holds for the same behavior holding for the sequential entangling gates. Thus, the state fidelities calculated experimentally according to the approximate fidelity decrease faster as a function of N_E than the exact values and therefore the quantum channel fit to the data produces a lower entangling gate fidelity. This observation suggests that the experimental entangling gate fidelities we obtain actually underestimate the exact values.

Additionally, equations (73) and (75) were added to the Supplementary Information Sec. X.A expressing the state fidelity difference in terms of correlations of observables.

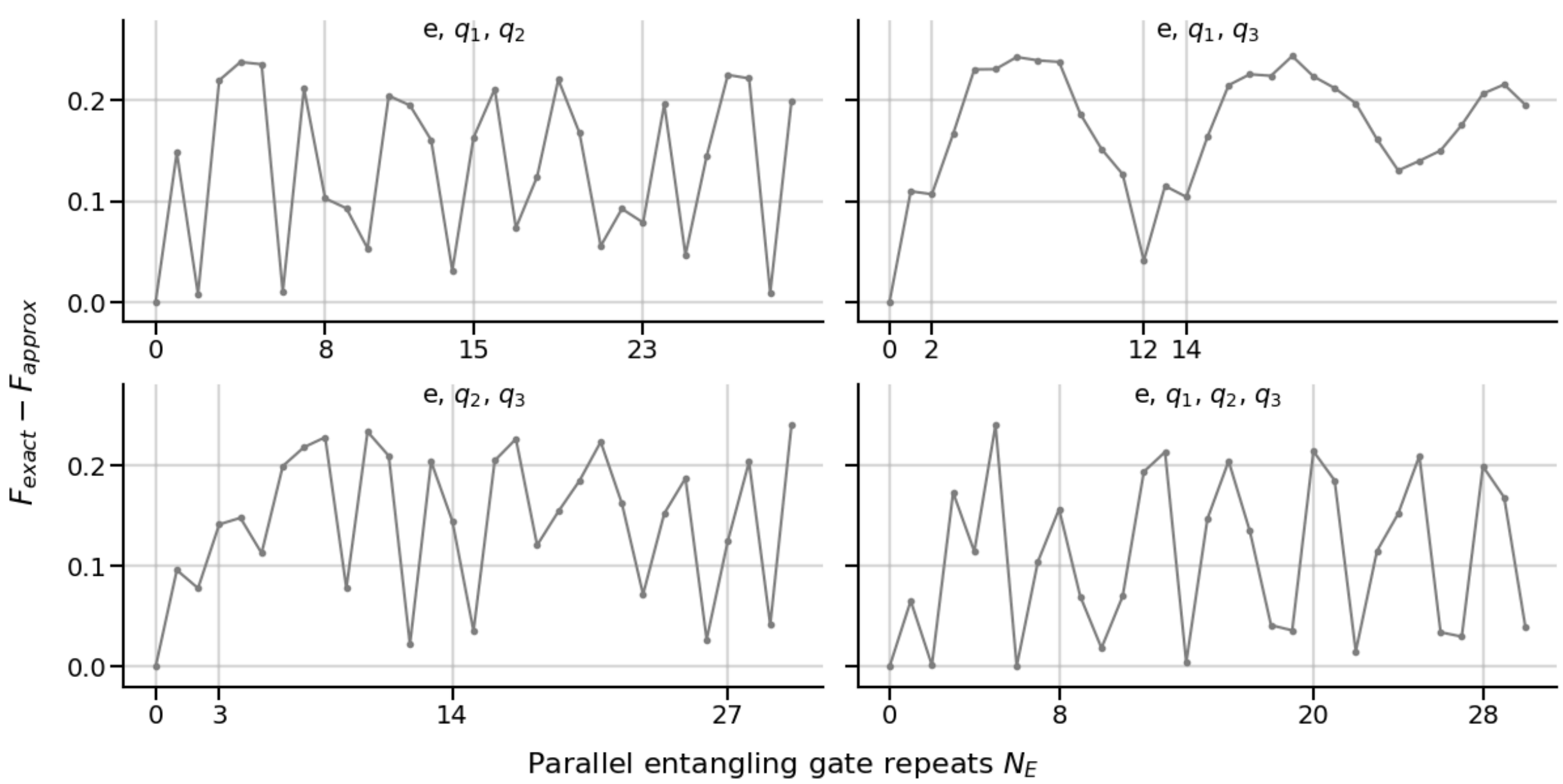

Supplementary Fig. 25 Difference between the exact and approximate state fidelities. F_{exact} is calculated based on equation equation (68) and F_{approx} is calculated based on equation (70).

Appropriate use of statistics

The data points have error bars, although it is missing how these error bars are calculated (probably from number of repetitions). On the other hand, the uncertainties from the fitted values are presented. Overall, additional explanations on how these statistics are calculated would be appreciated.

Response: We agree that additional details on data uncertainties and statistics would be helpful for readers.

Action taken: In all figure captions containing data points and fitting results, a description of the source of uncertainty is now included.

Conclusions, robustness, validity and reliability

For the current device, these results seem to be valid, and in good agreement with the simulations. However, there are a few key points that the authors should address to strengthen the impact of this work.

1. As mentioned previously, the main concern is up to which extent this method is applicable to future devices. In other words, are there any analytical conditions on relevant parameters such as hyperfine coupling strengths, t, N, number of nearby nuclei that these gates perform at high fidelity?

Response: We trust that our responses to earlier comments on this issue and especially as described in <u>Major action 1</u> addresses the reviewer's question regarding the distribution of hyperfine coupling strengths. Regarding the other parameters, a smaller N is advantageous for less error induced when controlling the electron (discussed in the Gate fidelity section of main text), however this needs to be balanced against the parameters of the desired nuclear operation. The unit pulse time t similarly can be calibrated using the analytical expressions described in our manuscript. Note that t must be chosen to lie outside the spin bath region. Otherwise, residual entanglement with bath spins will fundamentally limit its fidelity.

Action taken: The new section "Generality and extensions" demonstrates the straightforward design of gates for randomly chosen nuclear arrangements. To ensure unit pulse times in the bath region were not used for the parallel entangling gates set in the random registers sample in "Generality and extensions", we excluded them from the parameter search. The following text is from the Supplementary Information Sec. VI "Generality and extensions".

> Due to the presence of spin bath experimentally, additional care needs to be taken when sweeping potential unit pulse times. Unit pulse times the lie within the spin bath region will lead to significant residual entanglement with bath spins that are not being included in this model. To account for this, unit pulse times corresponding to the bath region were removed from the calibration algorithm. This avoided region was set based on the resonance times of the lower bound hyperfine coupling used.

2. It appears that uniform separations (i.e. near-equal spacings of hyperfine couplings at 40 kHz) simplifies finding a single t and N that addresses all targets. If hyperfine couplings are asymmetrically spaced and/or separated larger in their spacing, how does this affect the implementation of the DD gate?

Response: The approximately uniform separations of the parallel hyperfine parameters of our register are merely a coincidence and in fact do not simplify finding a single t and N that address all nuclear qubits. Rather, the crucial condition is that $(n_0 \cdot n_1) < 0$ for the targeted nuclear qubits. This condition accommodates nonuniform separations just as well. The extensive simulations of random registers in "Generality and extensions" (Major action 1) thoroughly highlights this fact.

3. Similarly, as shown in Fig 1c, if L=4, is it still possible to apply this method?

Response: Absolutely, it is possible for arbitrarily large numbers of nuclear qubits that satisfy the condition that $(n_0 \cdot n_1) < 0$. The new section "Generality and extensions" sections (see Major action 1) shows examples of L=4 and even L=5 cases. Takou et al, 2024 conducted simulations of even larger entangled states that can be created in parallel in different parameter regimes.

4. Since a requirement for this method is to have (n0 · n1) < 0, how does the gate fidelity change if (n0 · n1) becomes close to zero? Does this limit the observed gate fidelity?

Response: Based on the theoretical work of Takou et al, 2023, if $(n_0 \cdot n_1) < 0$, then there exists a N such that the first Mahklin invariant is zero: $G_1 = 0$, indicating maximal entanglement (perfectly locally equivalent to a CNOT). In practice, the question is whether one can identify a reasonably small N that minimizes G_1, and this limits the maximality of the entanglement. Nonetheless, we have shown that high-fidelity, practical gates are available even when $(n_0 \cdot n_1)$ only barely satisfies the condition. For example, $(n_0 \cdot n_1) = -0.11$ and -0.024 for qubits 1 and 3 in our work, and yet we can achieve a 4-qubit entangling power 0.993 with only N=6.

References: appropriate to credit existing work

Overall, the paper by Minnella et al is well-referenced, with relevant existing work credited where appropriate. However, when reviewing the previous work performed in solid-state spin registers, we believe Minnella et al omitted important work that utilises geometric phase gates to efficiently create multi-qubit entangling gates. This work was originally performed in nitrogen vacancy centres (Waldherr et al, Nature 506, 204-207 (2014)), and has been leveraged extensively in recent work in phosphorus atom qubits in silicon (see M. T. Mądzik et al, Nature 601, 348-353 (2022); H. Edlbauer et al., arXiv:2506.03567v1 (2025); and I. Thorvaldson et al., Nature Nanotechnology 20, 472-477 (2025)). Despite these operations being performed with different techniques to those explored by Minnella et al, we believe that this past work should be referenced in their manuscript.

Response: We thank the reviewer for pointing out these important and relevant works.

Action taken: Citations for all works mentioned have been added in the introduction, as well as a discussion of their differences to our work.

Clarity and context

In the main text, the "order" k was somewhat unclear on first reading (specifically in main text section I and the caption of Fig. 1). Can the authors please add a definition (or reference) to the main text to define this variable? If it is the same k appearing in methods section B, then an explicit reference to this would make it much clearer.

Response: Thank you for highlighting this point of confusion. Our revised manuscript clarifies the meaning of the variable k.

Action taken: The text in the introduction has now been expanded to include a definition, citation and functional description of the DD order k.

> Conceptually, t sets the period of electron state flips; when chosen to be in resonance with a target nuclear qubit, the DD sequence creates X-axis nuclear rotations. The resonance order, k, corresponds to the number of nuclear precession rotations in each period. As k increases, resonances separate due to differences in their hyperfine coupling [28].

Additionally, a note has been added to methods section B to explicitly declare k the same variable used in the introduction: "Here, the integer k specifies the DD order as discussed in the introduction".

In addition, we believe that the caption of Fig. 2a would be made clearer if the authors explicitly stated that the circuit in 2a was not used for the analysis performed in the rest of the paper – instead, the Z rotation of the electron was omitted. This would avoid confusion as 2a suggests oscillations should appear at a frequency given by M, in contrast to the experimental results which oscillate at a frequency of L.

Response: Thank you for pointing out this confusion. Section IIA does mention the omission of the electron Z gate in relation to the expected frequency: "Additionally, we only apply the variable phase gate Zφ to the nuclear qubits. As a result, when the electron is entangled with L nuclear qubits, we expect to measure a signal of the form:

$$P(|0\rangle_e) = 1/2(1 + cos(L\phi - \delta_L))"$$

We also think it is important and helpful to include Fig. 2a as the basic MQC method, however, we agree it was not clear which circuit was used in our experiments, and we have clarified this in the caption.

Action taken: The caption of Fig 2b now reads: "DD implementation of the MQC method used in this work, in which only the nuclear qubits are subject to Zφ gates, for bipartite entanglement with a single nuclear qubit", explicitly declaring this is the circuit used in this work and the change in phase gates applied.

Reviewer #2 (Comments for the Author):

In Minnella et al, the authors demonstrate a scheme to perform multipartite entanglement between a nitrogen vacancy center electron spin and three nearby 13C nuclear spins using a single entangling gate at room temperature. The multipartite entanglement uses a scheme that was theoretically developed by Takou et al. (refs 40, 41) that leverages the inherent crosstalk present in the always-on hyperfine interactions between electron and nuclear spins to generate maximally entangled states. The authors then generate three- and four-qubit GHZ states and adapt a method (Wei et al. PRA 2020) to verify the number of entangled spins by measuring multiple quantum coherences. They then estimate the fidelity of the four-qubit GHZ state (using a method adapted from Evered et al. Nature 2023), and show that the GHZ state preparation enabled by the single gate is faster and higher fidelity than the state preparation via serial single gates.

Prior work in the field has relied on such serial single gates with a central electron spin to enable generation of multiparticle entangled states, such as a seven-qubit GHZ state in Bradley & Randall et al. PRX (2019). In such work, care had to be taken to mitigate the effects of crosstalk between various electron-nuclear spin gates. Other solid-state defects such as rare-earth ions enable electron-nuclear and electron-electron selective couplings via frequency selectivity (see https://arxiv.org/abs/2508.09122), omitting the need for a central electron spin to act as mediator between all spins and potentially enabling the formation of maximally entangled states comprising multiple electron spins.

By aggregating technical advances from several other papers, this work advances the toolkit for generating, verifying and estimating the fidelity of multiparticle entangled states in solid-state spin registers. In general, I believe this work is technically robust, the results are interesting and that the paper will be useful for a broader audience aiming to prepare and apply multipartite entangled states.

Response: We thank the reviewer for taking the time to carefully review our paper and for the positive feedback.

However, the foundational ability to generate such a GHZ state with a central NV center electron spin has been present for some time. Given this, and the lack of substantial movement in this direction for several years, the paper would be strengthened if the utility of the faster, higher fidelity single gate scheme developed in this work was more fleshed out. If the authors can quantitatively substantiate the utility of this work and address the minor comments below, I would support publication in Nature Nanotechnology.

Response: We thank the reviewer for supporting publication with revisions to address this point. While the ability to generate GHZ states in NV-nuclear registers has indeed existed for some time, those earlier examples all relied on serial gate applications that operate far from the limit given by the hyperfine coupling strength, making them susceptible to decoherence and crosstalk errors. Moreover, they generally relied on specific arrangements of strongly-coupled nuclei, strong magnetic fields, cryogenic temperatures, or all of these conditions. (See Major action 1 regarding the difference between the strong- and weak-coupling regimes, and our

response to Reviewer 1 regarding the limits on gate speed.) That said, we certainly agree that it is helpful to highlight how the parallel entangling approach can be applied to practical tasks in quantum information science. To that end, we have added a new section to the Supplemental Information, "Applications to quantum error correction (QEC)", to quantitatively substantiate its utility.

Action Taken: See Major action 2 at the beginning of this document regarding revisions and additions to the text to discuss the implications for quantum error correction

Specifically, the authors claim that the multipartite entangled states can be useful for Heisenberg sensitivity scaling. To use this local register for quantum enhanced sensing, how would it work, and what is the sensitivity enhancement relative to serial single gates?

Response: The boost in sensitivity when leveraging entanglement (i.e., Heisenberg sensitivity scaling) is based on multiple factors and is dependent on the specific sensing scheme employed. In general, the sensitivity improves with larger, higher fidelity entangled states that are able to interrogate the source for longer, but it also depends on the overhead associated with creating and measuring such states. Thus, if the entangled states are created faster (less overhead) and with higher fidelity (more signal), the sensitivity will improve. The use of parallel entangling gates over sequences of two-qubit gates offers all of these advantages. Here, we have further demonstrated the control of such entangled states at room temperature and low magnetic fields, conditions amenable to nanoscale and biological/biochemical sensing schemes.

It's additionally unclear how far this scheme can be scaled in terms of number of qubits, and how a large quantum register of this type will be useful for any quantum computing applications.

Response: We appreciate this practical question. To address it, we added an additional section "Generality and extensions" to the main text, showing that registers supporting parallel entanglement of up to four or five ^{13}C qubits (in addition to ^{14}N) are readily available at room-temperature for NV centers in natural isotopic-abundance diamond. The register size can be further increased by lowering the temperature, which increases the electron T_1 and the corresponding spectral resolution of spins from the bath. Isotopic engineering can also serve to optimize the statistical arrangement of ^{13}C atoms.

Regarding the use in applications, numerous studies have shown how nuclear registers can be used to improve quantum sensors, quantum network nodes, and to implement fault-tolerant quantum information processing. In all these applications, both the size and quality of the register determines its utility.

Action taken: See Major action 1 at the beginning of this document for a discussion of scaling, and Major action 2 for a discussion of applications (in this case, QEC).

In principle, these gates could help advance quantum networking by enabling different quantum codes at individual network nodes, but some numbers would be useful here. How much better could a certain quantum code be, given this increase in multi-qubit gate fidelity and speed?

Response: As part of the new “Applications to quantum error correction” section in the Supplementary Information, we simulated the three-qubit bit flip quantum code, highlighting the improvement from the parallel entangling gate. And we agree with the reviewer’s assessment of the potential for such operations to help advance quantum networking; in fact, such a code was recently experimentally explored in generating hybrid entanglement with photons for quantum networking applications (see Chang et al. *Nature Physics* 2025).

Small comments:
1. How many qubits can this scheme scale to?

Response: Searching for parallel entangling gates similar to the ones used in this work, under similar conditions (i.e., room temperature and natural ^{13}C isotopic abundance) six-qubit (L=5) cases are possible in about 1.4% of weakly coupled registers. This scale is particularly important for fault-tolerant applications. Even larger cases are possible under different conditions (see simulations of Takou et al, 2024) .

Action taken: Please refer to Major action 1, regarding the generality and scaling potential of the method.

If the diamond is isotopically enriched with 13C, how many spins would be addressable?

Response: This is an interesting question and a possible direction of future research. We took initial steps to address it using the simulation framework from the “Generality and extensions” section with variable concentrations of nuclear qubits. As the nuclear concentration increases, the total nuclear qubit register size increases as well (see Supplementary Fig. 12 below). However, this does not ensure that larger entangled states are possible, since there is also more opportunity for residual entanglement to hinder parallel gates. Overall, L=5 parallel entangling gates are still the largest we observe.

Action taken: The results of the simulations using different concentrations of nuclear qubits were added to the Supplementary Information Sec. VI along with the following discussion.

> One can also consider diamond samples that have different concentrations of ^{13}C nuclei using this framework. On one side, with higher concentrations, there are more weakly coupled nuclear qubits in the register to participate in the parallel entanglement (Fig. 12a), but on the other side, this may lead to increased residual entanglement and in turn lower probabilities of such a parallel gate existing. Similarly, with lower concentrations, the register size decreases but so does the potential residual entanglement. Owing to the computational complexity associated with including more nuclear qubits in a register, smaller sample sizes (200 configurations) and unit pulse time steps (10ns) were used

when considering different concentrations. A primary bottleneck in running the parallel entanglement algorithm with a large number configurations, each with a large number of nuclei in the system, is the evaluation of every possible subset of nuclear qubits to target entanglement with. This power set of Nqubits nuclear qubits has an exponentially large size of 2Nqubits so for very large system sizes nearing ≈ 20 nuclear qubits, there are ≈ 1 million subsets to check over all gate parameters. In this case, pre-filtering for nuclear qubits that have an intersection of $(n_0 \cdot n_1)^{(l)} < 0$ before iterating though all gate parameters would be essential for large scale calculations. At lower concentrations, there tend to be lower likelihoods to form larger parallel entangled states (Fig. 12b), which can be attributed to the smaller register sizes. Similarly, as concentration increases, there tends to be a loss of likelihood. However, in these cases, fewer configurations pass the pre-filtering process for containing a strongly coupled nuclear qubit. If one conditions the likelihood on the register being only weakly coupled (Fig. 12c), then at increased concentrations there is a similar, if not slightly larger likelihood to form larger entangled states in parallel. Note, a finer-grained parameter space of DD gates may further accentuate these changes. Future research taking into these computational complexities can be dedicated to finding an optimal concentration, where optimality can be defined according to likelihood of parallel entanglement, size of the entangled state, duration or fidelity of the gate.

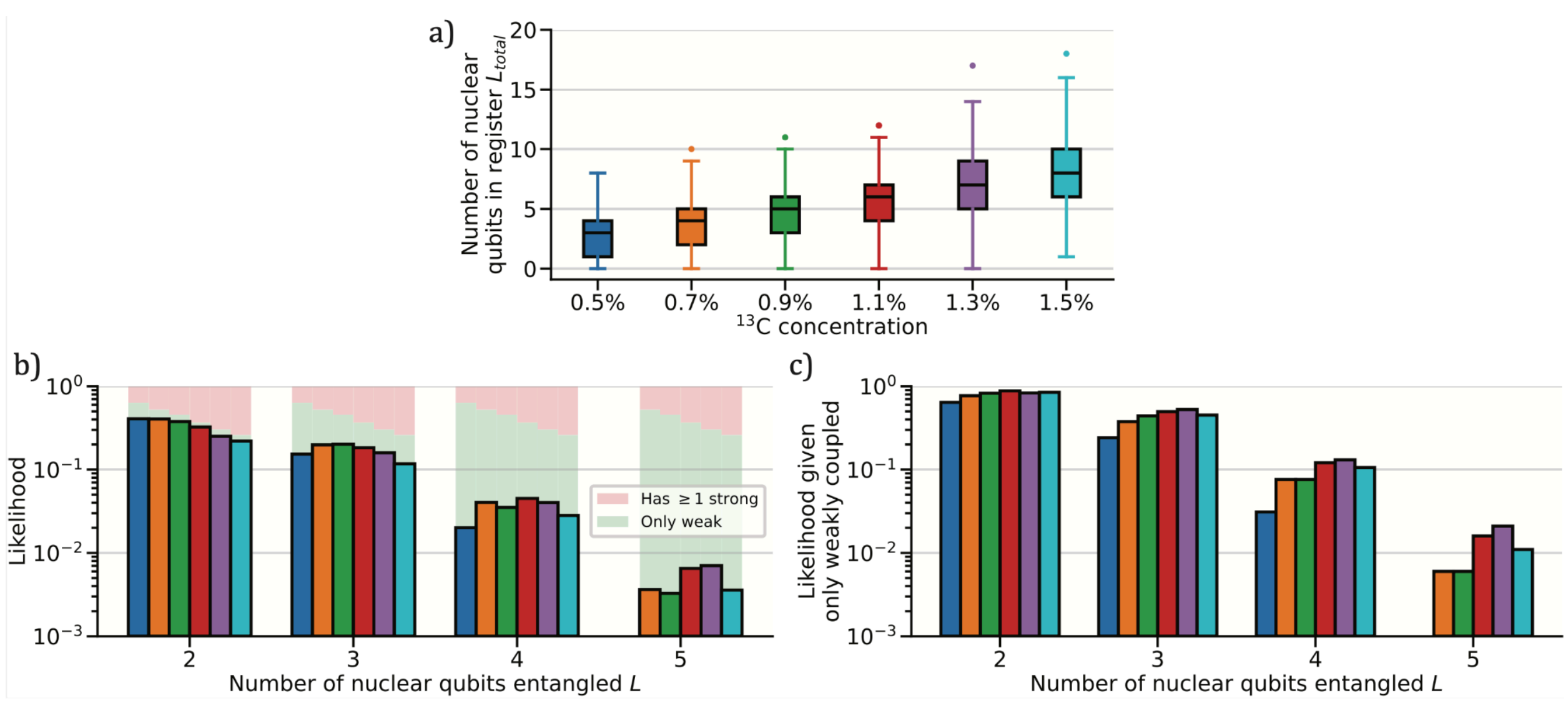

Supplementary Fig. 12 Nuclear qubit concentration effects on parallel entanglement (a) Total number of weakly coupled nuclear qubits in register. (b) Probability of any configuration hosting parallel entanglement of different sizes L. The red shaded regions denote the portion of configurations that have at least one strongly coupled nuclear qubit and are not considered for parallel entanglement. The green shaded regions denote the rest of the configurations that are considered for parallel entanglement. As concentration increases, the probability of having at least one strongly coupled nuclear qubit increases. Bar colors correspond to the concentrations in (a). (c) Probability of a weakly coupled configuration hosting parallel entanglement.

Additionally, what changes if trying to address multiple electron spins?

Response: If the electron spins are not interacting, then each sub-register of nuclear spins will need to be addressed individually and entanglement between the two registers cannot form without some mediating quantum bus (e.g., photons). When addressing one sub-register with DD, assuming the electrons are proximal such that they cannot be individually addressed with microwaves, crosstalk on the other register will be induced. The nature of the crosstalk (conditional vs unconditional) will depend on the other spectator register interactions.

Another interesting case is when the electron spins are interacting and thus entanglement between the two sub-registers can form. Then, the collection of all nuclear qubits in both registers can be considered for parallel entanglement. This would take more work to develop fully, but it could lead to interesting results similar to Edlbauer et al, *Nature* (2025).

2. The use of k to index dynamical decoupling "orders" with different unit pulse time resonances in the main text was slightly opaque in regard to how exactly it was indexing these resonance times. Perhaps it is a matter of familiarity with the notation, but I believe it would make the actual DD sequences used here easier to understand if it were expanded upon at its introduction and not just in the methods.

Response: Thank you for highlighting this point of confusion.

Action taken: The text in the introduction has now been expanded to include a definition and functional description of the DD order k:

> Conceptually, t sets the period of electron state flips; when chosen to be in resonance with a target nuclear qubit, the DD sequence creates X-axis nuclear rotations. The resonance order, k, corresponds to the number of nuclear precession rotations in each period. As k increases, resonances separate due to differences in their hyperfine coupling [28].

3. Given the residual entanglement on the third nuclear spin in the L=2 GHZ states, is there any path forward to a protocol achieving isolated parallel entanglement on a subset of the nuclear spins addressable in the parallel order while maintaining superiority over sequential gates? That is, is there an N such that the residual entanglement is minimal while keeping gate times short and state fidelity high, and how does it scale with L? If not, how does that affect computational ability for the applications described in the paper, if at all?

Response: We appreciate this question, since we overlooked the details of optimizing L=2 gates in our original submission. Indeed, we can design gates that selectively address subsets of the register by optimizing N. We calibrated the L=2 parallel entangling gates using the non-unitary entangling power of Takou et al, 2024, rather than the unitary entangling power used in the original submission. See Major action 3 for further details.

The parallel L=2 gates are still superior to sequential gates. All L=2 parallel gates are faster, ranging from 1.5 to 2.7 times faster. The speed up is reduced compared to the L=3 parallel gate since a larger N was needed to minimize residual entanglement, and since the L=2

sequential gate requires fewer operations. The performance of these gates, unsurprisingly, is strongly dependent on the number of operations required. For example, the L=2 parallel gate for q1,q2 required N=29, or 58 electron operations. Meanwhile, the crosstalk between q1 and q2 for the sequential gates was rather small (see before and after MQC optimization Supplementary Information Sec IX.B Fig. 18). For the pair, the parallel gate has a slightly worse fidelity than the sequential approach. In the other two cases, the parallel gates showed an improved fidelity over the sequential approach, even though they required more electron operations than the sequential approach. In these cases, as in general, crosstalk is a significant limitation to the sequential entangling gate fidelity, and this problem is mitigated in the parallel approach.

As for scaling to larger system sizes, Fig 5b now quantifies how many subsets of size L are expected to be entangled in parallel. This simulation takes into account residual entanglement and the bound used on the non-unitary entangling power of 0.8 ensures comparable fidelities to that observed in our cases. Parallel gates that require a large N (max of N=50 was used in the search) may have reduced fidelity, but this can be compensated for with improved electron gates (e.g. pulse shaping, operation with ^{14}N initialized into the m_s=0 state) in future work.

Action taken: See <u>Major action 3</u> at the beginning of this document.

Additional revisions to the manuscript:

In addition to the revisions described above, we made minor revisions to the manuscript text in order to adhere to the journal's limits on words and references.

All changes to the main text are marked in the "diff" version of the manuscript. Primarily, we edited the Introduction, Section I, and Conclusions for conciseness.

The following references were removed:

- T. Xie, Z. Zhao, S. Xu, X. Kong, Z. Yang, M. Wang, Y. Wang, F. Shi, and J. Du, 99.92%-fidelity CNOT gates in solids by noise filtering, Physical Review Letters 130, 030601 (2023).
- J. R. Maze, P. L. Stanwix, J. S. Hodges, S. Hong, J. M. Taylor, P. Cappellaro, L. Jiang, M. G. Dutt, E. Togan, A. Zibrov, et al., Nanoscale magnetic sensing with an individual electronic spin in diamond, Nature 455, 644 (2008).
- I. Lovchinsky, A. Sushkov, E. Urbach, N. P. de Leon, S. Choi, K. De Greve, R. Evans, R. Gertner, E. Bersin, C. Müller, et al., Nuclear magnetic resonance detection and spectroscopy of single proteins using quantum logic, Science 351, 836 (2016).

And the following references were added:

- G. Waldherr, Y. Wang, S. Zaiser, M. Jamali, T. Schulte-Herbrüggen, H. Abe, T. Ohshima, J. Isoya, J. Du, P. Neumann, et al., Quantum error correction in a solid-state hybrid spin register, Nature 506, 204 (2014).
- I. Thorvaldson, D. Poulos, C. M. Moehle, S. H. Misha, H. Edlbauer, J. Reiner, H. Geng, B. Voisin, M. T. Jones, M. B. Donnelly, et al., Grover's algorithm in a four-qubit silicon processor above the fault-tolerant threshold, Nature Nanotechnology , 1 (2025).
- H. Edlbauer, J. Wang, A. S.-E. Huq, I. Thorvaldson, M. T. Jones, S. H. Misha, W. J. Pappas, C. M. Moehle, Y.-L. Hsueh, H. Bornemann, et al., An 11-qubit atom processor in silicon, Nature 648, 569 (2025).

Revisions and Responses to May 29 Reviewer Comments

In the following, Reviewers' comments are formatted in italic font. Responses to each comment and actions taken are formatted in normal font, with revisions to the text formatted in blue font.

Reviewer #2 (Comments for the Author):

The authors have addressed my questions and I am happy to support publication in Nature Nano as-is.

Response: We thank the reviewer for evaluating our revisions and for their support of our work.

Reviewer #3 (Comments for the Author):

I am a new reviewer assigned in this review round. In addition to evaluating the work itself, I was asked to assess whether the concerns raised by Reviewer #1 have been adequately addressed. I have read the reviewer comments from the previous round, the authors' responses, and the revised manuscript. The main concerns raised by Reviewer #1 were the general applicability of the parallel entangling gate technique to future work in the field and its advancement beyond previous studies. These concerns have been addressed through revisions to the Introduction, the addition of a new section IV, and a brief discussion of applications to quantum error correction. In addition, I believe that all other technical concerns have been adequately addressed.

In the revised manuscript, the advantages of the parallel entangling gate and its general applicability are presented clearly. This work represents the first experimental demonstration of a parallel entangling gate between a central electron spin and weakly coupled multiple nuclear spins. The realization of a fast four-qubit entangling gate with moderately high fidelity is impressive. Therefore, I recommend publication of this work in Nature Nanotechnology after the authors address the following points.

Response: We thank the reviewer for their thorough evaluation of our revised paper and for evaluating our responses to the concerns originally raised by Reviewer #1.

In Table XI of the Supplementary Information, the parallel gate fidelity for q1 and q2 is lower than that of the sequential gate. On the other hand, QEC implemented with the parallel gate exhibits a much higher process fidelity than the sequential gate case, as shown in Fig. 26. Could the authors clarify why a higher QEC process fidelity is obtained despite the lower fidelity of the parallel gate?

Response: We thank the reviewer for highlighting this point. The main factor limiting the parallel three-qubit gates is electron gate error. For instance, the parallel three-qubit gate for nuclear qubits q1 and q2 required 58 electron operations, thus resulting in a lower gate fidelity than the sequential counterpart, which required 28 operations. The QEC simulations in the Supplementary Information do not include electron gate error. This choice was made to highlight the dramatic impact that crosstalk (either intentional in the parallel gate, or inherent in the sequential gate) alone has on the ability to perform QEC.

Action taken: A note has been added in Sec. XI. of the Supplementary Information to denote the absence of electron gate error while executing a DD operation: "Similarly, in both the parallel and sequential cases, register initialization is idealized and electron gate errors associated with DD sequences are ignored."

In addition, achieving very high-fidelity parallel entangling gates appears to be inherently challenging, as a parallel gate necessarily deviates slightly from the ideal conditional rotations. I agree that the parallel

gate can improve gate fidelity and reduce gate time, but is a moderately high-fidelity gate of this kind truly useful for quantum error correction, which typically requires operation above a stringent error correction threshold?

Response: The interpretation of "deviates slightly from ideal conditional rotations" can be taken two ways. On one hand, this can be taken to mean the error in the nuclear rotation angle through the discretization of the unit pulse repeats *N*. Such an error is captured by the entangling power metric, which is near unity (0.993) for the system studied in this work. Equivalently, the gate fidelity is likely even larger than this (see Takou et. al. 2023 for further details). Therefore, this discretization is a very small inherent error of the protocol. On the other hand, the reviewer may be concerned that each nuclear qubit is not rotated according to a $C_eX_{\pi/2}$ operation. This consequence of the parallel gates does not inherently limit the fidelity, as the entanglement is near a maximum, just in a different basis set by the gate geometry. In QEC use cases, additional local gates can be used to compensate for this effect.

In either case, it is true that QEC itself requires operations above a threshold to be beyond break-even (able to correct errors more than create new ones in the process). The QEC simulations show such behaviour, with the "QEC on" curves improving process fidelity compared to the "QEC off" curves. The execution of the parallel gates in these simulations take into account the two interpretations discussed above, indicating that their fidelity is sufficiently high to improve operation (correct errors). Separately, one can assess whether the improved operation is sufficiently high fidelity for fault-tolerant quantum computation. This is a more complex question depending on the use case. Nonetheless, the fidelities demonstrated are competitive with other leading platforms.

If I understand correctly, in the MQC protocol, any unconditional rotations of spectator qubits caused by crosstalk do not affect the measured result. Are these rotations corrected, or are they simply ignored? Furthermore, do unconditional rotations of spectator qubits have any impact on the measured entangling gate fidelities?

Response: This understanding is correct, unconditional rotations of spectator qubits do not affect the MQC signal or the measured entangling gate fidelities. Spectator qubits are not initialized and thus remain maximally mixed. Unconditional rotations of maximally mixed states trivially transform the state and thus do not affect other qubits in the register.

In Fig. 3e, CX_pi/2 should be CR_psi2.

Response: This is a subtle point, but $CX_{\pi/2}$ is the correct gate. This is because the unit pulse time used for the parallel 4-qubit gate is effectively on resonance for q2. This sets the rotation axes to the *X* axis. Similarly, the number of unit pulse repeats creates a $\pi/2$ rotation.

Action taken: To highlight this point, text has been added to the caption of Fig. 3: "The parallel gate unit pulse time is effectively on resonance for q_2, creating a $CX_{\pi/2}$ rotation."

In Fig. 3c–f, the red (blue) curves correspond to the parallel (sequential) gate, respectively. However, the opposite color is used in Fig. 4d,e. In addition, the labels for the parallel and sequential gates in Fig. 4f,g appear to be incorrect.

Response: We thank the reviewer for pointing out this mistake.

Action taken: The parallel and sequential colors in Fig. 3 and 4 are now consistent with one another and the legend in Fig. 4 has been corrected.
